# Efficacy of home-based inspiratory muscle training in patients post-covid-19: Protocol for a randomized clinical trial

**DOI:** 10.1371/journal.pone.0279310

**Published:** 2023-05-04

**Authors:** Gabriely Azevêdo Gonçalo Silva, Ivan Daniel Bezerra Nogueira, Gaspar Rogério da Silva Chiappa, Patrícia Angélica de Miranda Silva Nogueira

**Affiliations:** 1 Postgraduate Program in Physiotherapy (PPGFIS), Laboratory of Measures and Evaluation in Health, Federal University of Rio Grande do Norte (UFRN), Natal, Rio Grande do Norte, Brazil; 2 Department of Physical Therapy, Federal University of Rio Grande do Norte (UFRN), Natal, Rio Grande do Norte, Brazil; 3 University Center of Anápolis (Evangelical University of Goiás), Human Movement and Rehabilitation Graduate Program, Anápolis, Goiás, Brazil; Universidade Federal da Bahia, BRAZIL

## Abstract

**Introduction:**

Current evidence suggests the emergence of a novel syndrome (long COVID syndrome) due to sequels and persistent COVID-19 symptoms. Respiratory muscle training improves respiratory muscle strength, exercise capacity, diaphragm thickness, and dyspnea, especially in patients with decreased respiratory muscle strength. This study aims to evaluate the effectiveness of a protocol for home-based inspiratory muscle training to improve respiratory muscle strength, dyspnea, and quality of life of patients post-COVID-19.

**Methods and analyses:**

This randomized, controlled, double-blind clinical trial will be conducted at the Instituto de Medicina Tropical of Universidade Federal do Rio Grande do Norte (Brazil). Sample size will be determined using maximal inspiratory pressure after a pilot study with five patients per group (total of 10 patients). Patients included in the study will be evaluated in three moments: pre-training (initial), post-training (three weeks), and retention (24 weeks). The sample will be randomized in two groups: active (IMT using 30% of IMT and load increase of 10% of initial IMT every week. Patients will perform 30 repetitions, twice a day (morning and afternoon), for seven consecutive days, and six weeks) and SHAM (IMT without load). The following measurements will be assessed: anthropometry, respiratory muscle strength, pulmonary volume and capacity, dyspnea, perception of effort and lower limb fatigue, handgrip strength, functional capacity, anxiety, depression, and functional status. After initial evaluation, all patients will receive a POWERbreathe® (POWERbreathe®, HaB Ltd, Southam, UK) device to perform the training. Normality will be verified using Shapiro-Wilk or Kolmogorov-Smirnov, according to the number of patients included. Variables presenting nonparametric distribution will be compared using Wilcoxon (intragroup analysis) and Mann-Whitney test (intergroup analysis), whereas repeated measures two-way ANOVA will be performed in case of parametric distribution. Dunn’s post hoc test will be used to identify significant differences in the two-way ANOVA test.

**Primary outcomes:**

Respiratory muscle strength, dyspnea, and quality of life of post-COVID-19 patients.

**Second outcomes:**

Pulmonary function, dyspnea, exercise tolerance, handgrip strength, anxiety, depression, and functional status.

**Trial registration:**

Trial register number NCT05077241.

## Introduction

Researchers estimated that 81% of confirmed COVID-19 cases are mild, with mean recovery time of two weeks; 14% progress to severe pneumonia and 5% to severe acute respiratory syndrome, sepsis, and multiple organ failure [1]. Older adults are more affected and present 8% to 15% lethality [2].

Lungs are the most affected organ. Cough, sputum production, and shortness of breath are the most common symptoms after fever [3]. Extrapulmonary manifestations (e.g., cardiovascular, gastrointestinal, neurological, hepatic, renal, cutaneous, and hematological) also contribute to complications after the acute phase of the disease [4].

Evaluation of patients 60 days after onset of first symptoms showed that only 12.6% were free of COVID-19 symptoms, 32% presented one or two symptoms, and 55% three or more symptoms. Worse quality of life was observed in 44.1% of patients. Fatigue (53.1%), dyspnea (43.4%), joint pain (27.3%), and chest pain (21.7%) were also reported. Carfi et al. (2020) [5] highlighted that 87.4% of patients post-COVID-19 reported persistence of at least one symptom, mainly fatigue and dyspnea.

Six to twenty percent of post-COVID-19 patients present mild to moderate muscle weakness after 6 to 8 weeks of hospital discharge. Approximately one-third presented persistent pulmonary function impairment after one year of follow-up. Health status of COVID-19 survivors was worse than the non-infected population. Abnormal chest radiography, persistent reduction of exercise capacity, and impaired musculoskeletal performance and quality of life were also reported [6].

Dyspnea is one of the main respiratory symptoms after COVID-19 and may be associated with reduced respiratory muscle strength. Performance of respiratory muscles might be affected by several factors: aging, obesity, sedentary behavior, smoking, and chronic diseases. The increased load to respiratory muscles in patients with chronic pulmonary disease is due to reduced respiratory muscle strength and changes in airway resistance and chest wall mechanics. Long-term pulmonary impairments (e.g., fibrotic interstitial lung disease) might also be developed after the disease and may be associated with chronic inflammation caused by COVID-19 [6]. Thus, patients infected may present imbalance between respiratory muscle strength and respiratory demand, increasing the risk of respiratory failure [7].

Viral respiratory diseases are also associated with acute and long-term psychopathological consequences [8]. Patients post-COVID-19 may present delirium, anxiety, depression, and insomnia. These symptoms are induced by sequels of viral infection in the central nervous system or indirectly through immune response [9]. Mazza et al. (2020) [10] investigated psychiatric symptoms of 402 post-COVID-19 patients and demonstrated signs of post-traumatic stress disorder (28%), depression (31%), anxiety (42%), obsessive-compulsive disorder (20%), and insomnia (40%). This study also observed that systemic immune-inflammation index of patients was positively associated with depression and anxiety. Therefore, psychopathology of patients who survived COVID-19 must be assessed to diagnose and treat emergent psychiatric conditions [11].

A pilot study carried out with 42 participants recovered from covid-19 after mechanical ventilation, with a two-week IMT protocol (2x a day, 5x a week, 6 inspiratory cycles with each cycle for 5 minutes interspersed with 60 seconds of rest) improves function pulmonary function (FVC and FEV1), dyspnea, functional performance (6MWT) and QoL [12].

Another study involving 281 participants in the post-covid period, with an 8-week IMT program (3x week, up to 6 times of 6 inspirations with the rest periods interspersed with each inspiration progressively decreasing from 40 to 10 s with each block, producing durations 20-min session maximums) showed significant improvements in the intervention group for the “shortness of breath and activity” and “psychological” subdomains of the KBILD quality of life questionnaire. Greater reduction in dyspnea was also observed, improvement in inspiratory muscle strength, increase in functional capacity and levels of physical activity [13].

## Objectives

### Primary objective

This study aimed to evaluate the effectiveness of a home-based inspiratory muscle training (IMT) protocol compared to an IMT SHAM group for improving respiratory muscle strength, dyspnea, and quality of life of post-COVID-19 patients.

### Secondary objective

To evaluate the influence of IMT on pulmonary function, perception of effort, dyspnea, exercise tolerance, handgrip strength, anxiety, depression, and functional status.

## Material and methods

### Type of study and location of research

This randomized, controlled, double-blind, parallel, two arm clinical trial will be conducted at the Instituto de Medicina Tropical and of Universidade Federal do Rio Grande do Norte, referred from the ambulatory of infectology of Hospital Giselda Trigueiro (Rio Grande do Norte, Brazil) will be recruited.

Sample size will be determined using maximal inspiratory pressure (MIP) (GPower Software version 3.1.9.2, Kiel, Germany) after a pilot study with five patients in each group (total of 10 patients). Mean and standard deviation will be used to estimate effect size, and a bilateral distribution with alpha error of 0.05 and 80% power will be adopted. Sample size will be adjusted assuming a dropout rate of 20%.

### Inclusion criteria

Sedentary patients of both genders, diagnosed with COVID-19 using reverse transcription polymerase chain reaction, aged above 18 years, without other respiratory diseases associated or cardiac disease, with adequate cognitive capacity (Mini-Mental State Examination [MMSE]), and reduced respiratory muscle strength will be included. Reduced inspiratory muscle strength will be defined using MIP [14].

### Exclusion criteria

Patients with conditions impairing assessments, health complications that justify interrupting data collection (e.g., syncope, intense chest pain, or cough with blood-tinged sputum), hospitalized due to exacerbation of clinical conditions, or requested to leave the study will be excluded.

Regarding recruitment, researchers will contact the Hospital Giselda Trigueiro and inform patients about the study.

The sample will be randomized (www.randomization.com) in two groups: active (AG, IMT with load) and SHAM (SG, placebo IMT without load). Groups will be coded, and allocation will be included in sealed and opaque envelopes numbered consecutively.

This study will include three investigators: the first will be responsible for assessments; the second, adjustment of training load; and the third, randomization. This is a double-blinded study because the first researcher and patients will be blinded to allocation of patients and intervention effects.

This protocol was approved by the research ethics committee of Universidade Federal do Rio Grande do Norte (Brazil) (CAAE**:** 45575421.7.0000.5537) on May 1, 2021 and submitted to Clinical Trials (NCT05077241). Autonomy and anonymity of patients will be respected, ensuring personal data privacy according to resolution nº 510/16 of the National Health Council and Declaration of Helsinki. Before entering the study, all patients will sign the informed consent form.

Our study was written in accordance with Standard Protocol Items: Recommendations for Interventional Trials (SPIRIT), which was aim to improve the quality of clinical trial (S1 Checklist).

### Interventions

Patients included will be evaluated in three moments: pre-training (initial), post-training (six weeks after pre-training), and retention (24 weeks after pre-training). After recruitment, one researcher previously trained and blinded to allocation will evaluate vital signs, anthropometric measurements, pulmonary volume, respiratory muscle strength, handgrip strength, quality of life, anxiety, depression, functional status, and functional capacity.

After initial evaluation, all patients will receive a POWERbreathe® (POWERbreathe®, HaB Ltd, Southam, UK) device and be individually oriented on how to perform the IMT protocol. An experimental session will be conducted for familiarization with the device. Patients will receive a phone call every three days from the second researcher (who will not participate in the evaluation) to confirm if IMT is being performed correctly and resolve doubts regarding the protocol. At the end of each week, patients from EG will receive a video call from the second researcher to adjust load of the device.

### Data collection

**Fig 1:**
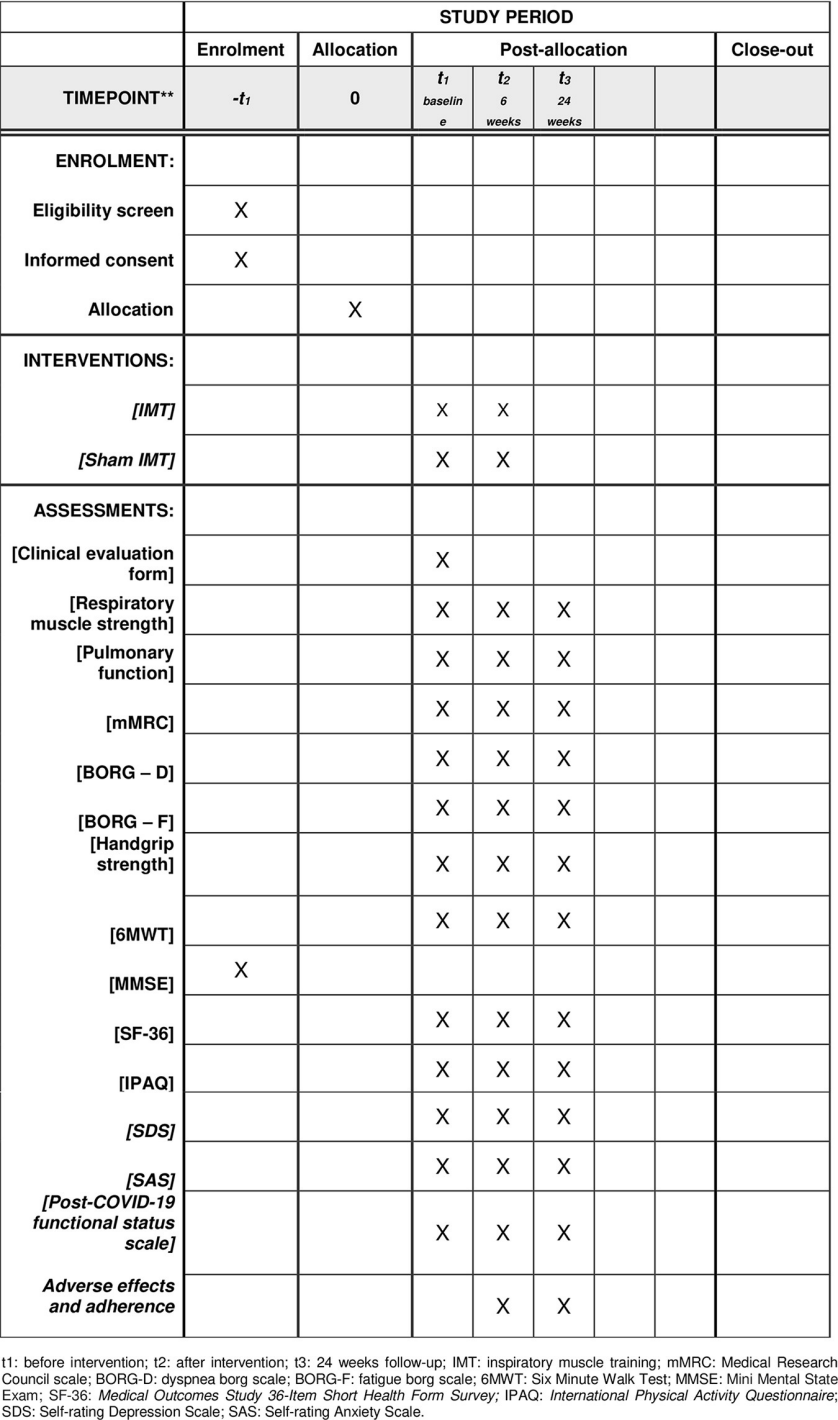
Template of recommended content for the schedule of enrolment, interventions and assessments. t1: before intervention; t2: after intervention; t3: 24 weeks follow-up; IMT: inspiratory muscle training; mMRC: Medical Research Council scale; BORG-D: dyspnea borg scale; BORG-F: fatigue borg scale; 6MWT: Six Minute Walk Test; MMSE: Mini Mental State Exam; SF-36: *Medical Outcomes Study 36-Item Short Health Form Survey;* IPAQ: *International Physical Activity Questionnaire*; SDS: Self-rating Depression Scale; SAS: Self-rating Anxiety Scale.

The tests and questionnaires used for these assessments are listed along with their outcome variables in Fig 1. (The recommendations for Interventional Trials (SPIRIT) schedule of enrollment, interventions, and assessments).

#### Clinical evaluation form

After signing the informed consent form, patients will be assessed using an evaluation form. This form will assist collection of personal data, vital signs, anthropometric measurements, personal and pathological background, lifestyle habits, physical examination (inspection and palpation), and complementary exams.

#### Respiratory muscle strength

Respiratory muscle strength will be assessed by measuring MIP and maximal expiratory pressure (MEP) using an analog manovacuometer (GERAR^®^, São Paulo, Brazil).

Patients will use a nasal clip and remain seated comfortably in a chair with back support, hip angle of 90°, upper limbs resting on legs, and feet on floor. To measure MIP, patients will perform maximal expiration until residual volume, place the mouthpiece, and perform a maximal inspiratory effort against an occluded airway, and sustain for a minimum of one second. To measure MEP, the patient will inspire close to total lung capacity, place the mouthpiece, and perform a maximal expiratory effort against the occluded airway, maintaining for at least one second [14]. Patients will be constantly encouraged by the researcher during the test.

For data analysis, at least three reproducible maneuvers will be performed with variability lower than 20%; the highest value will be registered. References values for the Brazilian population will be calculated according to age and gender [14].

#### Pulmonary function

Pulmonary volume and capacity will be evaluated using a calibrated spirometer (Koko Digidoser model, Spide, Longmont, USA) in an acclimatized room. Patients will be advised to avoid large meals one hour before the test, food and drinks with caffeine for at least six hours before the test, and alcoholic drinks on the day of the test [15].

The test will consist in performing an inspiratory maneuver until total lung capacity, followed by maximal and forced expiration to residual volume using the spirometer. Tests will be performed with patients seated and hips and ankles flexed at 90º [13]. A minimum of three tests will be conducted with variation less than 5%; the highest forced expiratory volume in the first second and forced vital capacity obtained will be compared with predicted values for the Brazilian population [16].

#### Dyspnea

Dyspnea will be assessed using the Medical Research Council scale adapted to Portuguese. Patients must indicate the impact of dyspnea on mobility during daily activities. Scores range between 1 and 5, and highest values indicate greater dyspnea [17].

#### Perception of effort and fatigue

Patients will be questioned about subjective perception of respiratory effort and lower limb fatigue before and after training using the modified Borg rating of perceived exertion (10-Borg) scale. Higher values correspond to greater sensation of dyspnea.

#### Handgrip strength

Handgrip strength will be measured using a hydraulic handheld dynamometer (Saehan®). Maximal isometric strength of the dominant hand will be assessed with patients seated comfortably in a chair, with knee and ankle joints at 90º, shoulder of dominant arm forward and in neutral rotation, elbow flexed at 90º near upper body, forearm in neutral position, and wrist between 0º to 30º of extension and 0º to 15º of ulnar deviation. Participants will be requested to perform maximal isometric strength for five seconds without moving other body parts [18]. Three measurements will be performed with one-minute interval in between, and the average will be considered for analysis. Patients will be considered fragile if the average of three measurements is between 20% of lower distribution values, according to the World Health Organization [19, 20].

#### Six-minute walking test

The 6MWT evaluates submaximal functional capacity by measuring the distance walked on a 30-meter flat surface for six minutes. Patients will be instructed to walk as fast as possible without running and stop to rest when needed. The researcher will monitor heart rate, oxygen saturation, dyspnea, and lower limb fatigue at each lap. Standardized verbal stimulus will be given every minute, as recommended (ATS, 2002). The distance walked after six minutes and predicted values based on gender, weight, age, and height will be included for analysis [21].

Arterial blood pressure, heart rate, oxygen saturation, and Borg will also be assessed before and after the test. The test will be interrupted if patients present chest pain, intolerable dyspnea, oxygen saturation < 85%, cramps, sweating, paleness, or vertigo [21].

#### Cognitive evaluation

The MMSE will be used to screen cognitive function. The test consists of two sections that assess orientation to time, orientation to place, registration, calculation, repetition, 3-step command, reading and obeying, recall, naming, writing, and copying. Total score ranges from 0 to 30, and higher scores indicate better cognitive performance [22]. Cut-off points will be used to minimize the influence of educational level in total scores [23].

#### Medical outcomes study 36-item short health form survey

The Medical Outcomes Study 36-Item Short Health Form Survey (SF-36) is a multidimensional questionnaire translated and validated to the Brazilian population [24, 25]. SF-36 evaluates health-related quality of life using 36 items distributed into eight domains. These domains encompass two components: physical (physical aspects, bodily pain, general health status, and physical functioning) and mental (emotional status, social functioning, mental health, and vitality). Subscale score ranges from 0 to 100, and higher scores indicate better health-related quality of life [26].

#### International physical activity questionnaire

The extended version of the International Physical Activity Questionnaire (IPAQ), adapted [25] and validated to the Brazilian population [28], will be used to assess duration and frequency of moderate or vigorous physical activities in the last seven days. It comprises 15 questions divided into five domains: job-related, transportation, household, recreation, and leisure.

Trained evaluators will apply the IPAQ as an interview, and older people must answer the questionnaire considering the actual week. Energy expenditure for each activity will be estimated in metabolic equivalents considering minutes of physical activity performed per week [27–29]. The people will be classified as very active, active, irregularly active, or sedentary [30].

#### Depression and anxiety scale

The self-rating depression scale (SDS) and the self-rating anxiety scale (SAS) will be used to screen patients for depression and anxiety, respectively. SDS and SAS have 20 items with scores ranging from 1 to 4; higher scores indicate higher severity of depression and anxiety [31].

#### Post-COVID-19 functional status scale

The post-COVID-19 functional status scale analyzes relevant aspects of daily life after the infection. The scale assists users in recognizing current functional limitations post-COVID-19 and determines the degree of functional limitations. It contains six items ranging from 0 to 5 that cover functional outcomes focusing on limitations in activities of daily living (e.g., household activities, professional/study activities, and lifestyle changes). Higher scores indicate worst functional status [32].

#### Adverse effects and adherence

Adverse effects and adherence will be assessed using a training diary will be given to all patients to assess adverse effects and adherence. After all training sessions, they will be encouraged to fill the diary, registering positive and negative observations and complications during and after training. Adherence will be assessed considering every time patients register the realization of the session. Fulfilled sessions will be added and then divided by the total number of sessions patients could perform.

Adherence to training will be considered by verifying the number of days that patients performed the sessions. The sum of all sessions performed will be divided by the total number of sessions that patients must perform. Adverse effects will consider all complications registered in the diary or mentioned during final evaluations.

#### Intervention protocol

The following training protocols will be applied.

Active group protocol: IMT using 30% of MIP and load increase of 10% of initial MIP every week. Patients will perform 30 repetitions, twice a day (morning and afternoon), for seven consecutive days, and six weeks. Patients will be instructed to perform a fast inspiratory muscle contraction, sustain it for two seconds, and expire. Thirty seconds of rest will be allowed every three IMT repetitions to avoid muscle fatigue or complications.

Sham group protocol: patients will use an IMT device without load and receive the same instructions as EG. If the treatment applied to EG be effective, we will invite patients from CG at the end of the study to perform the IMT protocol with load.

### Statistical analysis

SPSS software version 22.0 (IBM Corp. USA) will be used for data analysis. Normality will be verified using Shapiro-Wilk or Kolmogorov-Smirnov, according to the number of patients included. Variables presenting nonparametric distribution will be compared using Wilcoxon (intragroup analysis) and Mann-Whitney test (intergroup analysis), whereas repeated measures two-way ANOVA will be performed in case of parametric distribution. Dunn’s post hoc test will be used to identify significant differences in the two-way ANOVA test.

Descriptive analyses will be conducted using mean and standard deviation or median and interquartile range (25% - 75%), according to data distribution. A significance level of 5% will be applied to minimize type I error. The power of the study and effect size will also be performed for the main results of the study.

## Supporting information

S1 Checklist SPIRIT 2013 checklist: Recommended items to address in a clinical trial protocol and related documents*. (DOC)

S1 File Opinion of the research ethics committee (Portuguese). (PDF)

S2 File Opinion of the research ethics committee (English). (PDF)

S3 File Research project submitted to the ethics committee (Portuguese). (PDF)

S4 File Research project submitted to the ethics committee (English). (DOCX)
