# Supplementary material for: Efficacy of home-based inspiratory muscle training in patients post-covid-19: Protocol for a randomized clinical trial
Source: PLoS One. 2023 May 4;18(5):e0279310. doi: 10.1371/journal.pone.0279310 (PMC10159136; doi:10.1371/journal.pone.0279310)
Supplement: S1 File — (PDF) [file pone.0279310.s002.pdf]

PARECER CONSUBSTANCIADO DO CEP

DADOS DO PROJETO DE PESQUISA

Título da Pesquisa: Eficácia do treinamento muscular inspiratório domiciliar em pacientes pós-covid-19: ensaio clínico randomizado.

Pesquisador: Patrícia Angélica de Miranda Silva Nogueira

Área Temática:

Versão: 4

CAAE: 45575421.7.0000.5537

Instituição Proponente: Departamento de Fisioterapia

Patrocinador Principal: Financiamento Próprio

DADOS DO PARECER

Número do Parecer: 4.719.458

Apresentação do Projeto:

Projeto de doutorado do programa de pós-graduação em Fisioterapia, não prevê instituição coparticipante. Tem como objetivo "Avaliar a eficácia de um protocolo de treinamento muscular inspiratório domiciliar na melhora da força muscular respiratória, dispneia e qualidade de vida de pacientes pós-Covid-19". Realização da pesquisa de abril de 2021-2023. Número amostral a definir. Recrutará os participantes a partir do encaminhamento dos mesmos pelo Ambulatório de Infectologia do Hospital Giselda Trigueiro. Trata-se de um estudo de intervenção, experimental onde os sujeitos incluídos na pesquisa passarão por três momentos de avaliações: Pré-treinamento (Inicial), Pós-Treinamento (6 semanas) e Teste de Retenção (24 semanas).Após o recrutamento, os participantes serão convidados a comparecer ao Instituto de Medicina Tropical para realizar uma avaliação inicial por um avaliador previamente treinado e cego para o grupo de alocação da intervenção e incluirá anamnese e exame físico, com aferição dos sinais vitais, medidas antropométricas, avaliação dos volumes pulmonares, força muscular respiratória, força muscular periférica, qualidade de vida, ansiedade e depressão, status funcional e teste da caminhada de 6 minutos. Após a avaliação inicial, todos os voluntários receberão um aparelho POWERbreathe® (POWERbreathe®, HaB Ltd, Southam, UK), para realização do treinamento, e serão orientados individualmente sobre como utilizá-lo e sobre a realização do protocolo. Eles realizarão uma sessão experimental para familiarização com o dispositivo que não será considerada para

análise. A cada três dias os voluntários receberão uma ligação telefônica do pesquisador 2 que não participará da avaliação para confirmar se o exercício com o POWERbreathe® estava sendo realizado adequadamente na frequência e carga orientadas e se havia alguma dúvida em relação ao protocolo. Ao final de cada semana os participantes receberão uma ligação por vídeo do pesquisador 2 para realizar o ajuste no aparelho de acordo com a progressão de carga semanal do G1. Todos os momentos de avaliações (pré-treinamento, pós-treinamento e teste de retenção) serão realizados por um único avaliador (Avaliador 1) – o qual não saberá em qual grupo o sujeito será alocado – e registrados na ficha de avaliação desenvolvida para o projeto. Um segundo pesquisador (Avaliador 2) será o responsável por aplicar os protocolos de treinamento nos sujeitos. Critério de Inclusão: Serão considerados como critérios de inclusão sujeitos de ambos os sexos, sedentários, com diagnóstico de COVID-19 confirmado pelo RT-PCR, idade acima de 18 anos e sem qualquer doença respiratória de base, com estado cognitivo adequado definido através do Mini Exame do Estado Mental (MEEM) (ANEXO 1) e redução da força muscular respiratória, definida através da avaliação da Pimáx., de acordo com as seguintes equações de predição: homens (153,3 - 0,8 x idade) e mulheres (110,4 – 0,49 x idade) e considerando o limiar inferior de normalidade com erro padrão de estimativa (EPE) para homens de 17,3 e mulheres de 9,1 (NEDER et al., 1999). Critério de Exclusão: Serão excluídos do estudo sujeitos que apresentem alguma condição que impossibilite a realização das avaliações e dos protocolos, intercorrências que justifiquem a interrupção da coleta de dados, como síncope, dor torácica intensa, tosse com sangue, aqueles que solicitarem saída do estudo e que apresentem efeitos adversos como o internamento por agudização do quadro clínico. Metodologia de Análise de Dados: Para análise dos dados será utilizado o software SPSS (Statistical Package for the Social Sciences) versão 22.0 para Windows. O teste de normalidade será realizado de acordo com a quantidade de participantes voluntários da pesquisa. Portanto, poderá ser utilizado o teste de normalidade de ShapiroWilk ou o teste de normalidade de Kolmorov-Smirnov para as variáveis desejadas. As variáveis que apresentarem distribuição não paramétricas serão comparadas através dos testes Wilcoxon (análise intragrupos) e Mann-Whitney (análise intergrupos) e quando a distribuição ocorrer de forma paramétrica será utilizado o teste ANOVA two-way. Quando houver diferença significativa, o teste post hoc de Dunn's será aplicado a fim de se localizar as diferenças. Na análise descritiva, será realizada uma caracterização da população estudada, através da obtenção das médias e desvios-padrão para variáveis de distribuição normal ou mediana e intervalo interquartil (25%-75%) para variáveis de distribuição assimétrica. Para minimizar um eventual erro do tipo I será estabelecido o nível de significância de 5%. O poder do estudo e o tamanho do efeito serão

Endereço: Universidade Federal do Rio Grande do Norte ¿ Campus Central, s/n.
Bairro: Lagoa Nova **CEP:** 59.078-970
UF: RN **Município:** NATAL
Telefone: (84)3215-3135 **Fax:** (84)99193-6266 **E-mail:** cepufrn@reitoria.ufrn.br

expostos nos principais resultados do estudo.

Objetivo da Pesquisa:
Objetivo Primário:

Avaliar a eficácia de um protocolo de treinamento muscular inspiratório domiciliar na melhora da força muscular respiratória, dispneia e qualidade de vida de pacientes pós-Covid-19.

Objetivo Secundário:

Avaliar após TMI a influência da:

• Função pulmonar;

• Percepção de esforço e dispneia;

• Tolerância ao exercício;

• Qualidade de vida;

• Força muscular periférica;

• Ansiedade e depressão;

• Estado funcional.

Avaliação dos Riscos e Benefícios:
Os riscos e benefícios foram assim estimados no projeto:

Riscos:

Esta pesquisa apresenta riscos mínimos aos participantes. Estes podem se sentir constrangidos

durante a entrevista, tendo em vista a natureza do conteúdo de alguns questionários, que ele poderá recusar-se a responder, ou pela dificuldade em realizar alguma avaliação específica. Poderão apresentar alguns desconfortos como cefaleia, vertigens, náuseas, oscilação da pressão arterial e até um pequeno desequilíbrio devido a exigência de determinados testes físicos e treinamento, no entanto os avaliadores usarão critérios de contraindicações ou de interrupções dos testes quando necessário, para minimizar os riscos à saúde dos participantes. O participante poderá recusar-se a realizar qualquer procedimento a qualquer momento, sem que haja prejuízo para ele. Além disso, também podem apresentar preocupações quanto ao sigilo dos dados coletados e a assinatura que deverão realizar no TCLE. O pesquisador irá auxiliá -los esclarecendo todas as dúvidas existentes e informar aos participantes que os dados coletados serão utilizados apenas para fins acadêmicos, e que não haverá divulgação deles.

Benefícios:

Os resultados obtidos com o desenvolvimento desta pesquisa podem garantir a possibilidade de ampliar as intervenções relacionadas à reabilitação pulmonar em pacientes pós-covid-19, através de um treinamento efetivo, acessível, seguro e de baixo custo. As informações levantadas também poderão contribuir para o rastreamento de pacientes após o período de infecção aguda, contribuindo para o entendimento a cerca dessa nova temática, o desenvolvimento e avaliação da efetividade de intervenções direcionadas a melhorar a saúde dessa população. Porém, estas mesmas informações não constam no TCLE. Lá há uma subestimativa desses riscos.

Comentários e Considerações sobre a Pesquisa:

O projeto de pesquisa encontra-se bem estruturado, apresentando os elementos necessários a um trabalho de doutorado. Pretende contribuir com a reabilitação de pacientes que tiveram covid-19, através do treinamento da musculatura respiratória. A proposta, no entanto, necessita de alguns ajustes para atender as normativas éticas que tratam da pesquisa envolvendo seres humanos e que seguem descritas no item “Conclusões ou pendências e Lista de inadequações”.

Considerações sobre os Termos de apresentação obrigatória:

1) Folha de rosto.
2) Declaração de compromisso ético de não início da pesquisa.

3) Termo de confidencialidade. Está assinado apenas pelo pesquisador responsável e pela doutoranda. Como no desenho do estudo prevê a participação de 3 pesquisadores, deve-se esclarecer quem são esses membros da equipe de pesquisa e todos devem assinar o presente documento, bem como estarem incluídos no Projeto cadastrado na Plataforma Brasil.
4) Cronograma.
5) Orçamento – não informa sobre o dispositivo que ficará com os 10 participantes para realizar a intervenção aparelho POWERbreathe®. Quem irá custear a compra e disponibilização deste aparelho aos participantes? Não informa os diversos outros equipamentos e materiais que serão utilizados para avaliação dos participantes de pesquisa. Todos os equipamentos e materiais que serão necessários a realização da pesquisa deverá ser informados, mesmo que eles não sejam custeados pela pesquisadora responsável, mas pela proponente ou mesmo pela instituição coparticipante.
6) Projeto cadastrado na Plataforma Brasil. (Projeto PB).
7) Folha de identificação do pesquisador. Precisa identificar a instituição coparticipante – Hospital Giselda Trigueiro. Também cadastrar na Plataforma Brasil.
8) Carta de anuência do Instituto de Medicina Tropical.
9) TCLE não descreve a contento os riscos estimados para a intervenção. Não descreve todas as avaliações que os participantes irão passar. São em torno de 14 avaliações, e o TCLE descreve apenas algumas destas. Deve-se incluir também a aplicação da escala de depressão e ansiedade, escala de estado funcional pós-covid-19, entre outros também não descritos.

Recomendações:

Caro(a) pesquisador(a), se, em decorrência da pandemia do Corona vírus (COVID-19) o cronograma apresentado sofrer alteração, recomenda-se, ao se estabelecerem datas atualizadas, o envio de novo cronograma ao CEP Central/UFRN, sob a forma de notificação do tipo "Comunicação de Início do Projeto".

Conclusões ou Pendências e Lista de Inadequações:

De acordo com análise ética realizada considerando o disposto na resolução 466/2012 - CNS que trata da pesquisa envolvendo seres humanos, foram observadas as seguintes pendências:

PENDÊNCIA 01 – Instituições coparticipantes. Documentos: Projeto PB (cadastrado na Plataforma

Brasil) + folha de identificação do pesquisador. Devem ser informados como instituição coparticipante “aquela na qual haverá o desenvolvimento de alguma etapa da pesquisa. Esta é, portanto, uma instituição que participará do projeto, tal qual a proponente, apesar de não o ter proposto”. Nesse sentido, considerando que o recrutamento dos participantes ocorrerá a partir do ambulatório de infectologia do Hospital Giselda Trigueiro a mesma deve ser informada como instituição coparticipante nos documentos e deve apresentar carta de anuência. Da mesma forma, o Instituto de Medicina Tropical deverá ser informado como instituição coparticipante nos documentos citados anteriormente.
PENDÊNCIA 02 – Termo de confidencialidade + Projeto cadastrado na Plataforma Brasil. A pesquisa prevê a participação de 3 pesquisadores, no entanto o documento só apresenta a assinatura da pesquisadora responsável e sua orientanda. Nesse sentindo TODOS os membros da pesquisa deverão ser devidamente cadastrados no projeto da Plataforma Brasil bem como devem assinar conjuntamente o termo de confidencialidade assegurando conhecimento e responsabilidade para com o compromisso ali assumido.
PENDÊNCIA 03 – TCLE – Os riscos estimados ao longo do projeto e na plataforma Brasil não aparecem para o participante de pesquisa no TCLE. Nesse sentido, cabe reforçar que para o consentimento esclarecido do participante, a descrição na íntegra destes aspectos é essencial. Da mesma forma devem ser descritas todas as etapas de avaliação a que o participante será submetido.
PENDÊNCIA 04 – Cronograma. O cronograma de pesquisa prevê coleta de dados em 19 de abril. Considerando que o projeto foi submetido em 15/04 deve-se rever esta proposta de início do projeto, uma vez que a estimativa para início não contemplava o processo de análise do CEP.
PENDÊNCIA 05 – Orçamento – O orçamento não prevê os gastos com equipamentos e materiais previstos na pesquisa. Não informa sobre o dispositivo que ficará com os 10 participantes para realizar a intervenção aparelho POWERbreathe®. Quem irá custear a compra e disponibilização deste aparelho aos participantes? Não informa os diversos outros equipamentos e materiais que serão utilizados para avaliação dos participantes de pesquisa. Todos os equipamentos e materiais que serão necessários a realização da pesquisa deverá ser informados, mesmo que eles não sejam custeados pela pesquisadora responsável, mas pela proponente ou mesmo pela instituição coparticipante.

Em análise ética realizada no dia 05 de maio de 2021, considerando os novos documentos incluídos e a carta de resposta as pendências foram feitas as seguintes avaliações:

Endereço: Universidade Federal do Rio Grande do Norte ¿ Campus Central, s/n.
Bairro: Lagoa Nova **CEP:** 59.078-970
UF: RN **Município:** NATAL
Telefone: (84)3215-3135 **Fax:** (84)99193-6266 **E-mail:** cepufrn@reitoria.ufrn.br

Pendência nº 01 - Instituições coparticipantes. Documentos: Projeto PB (cadastrado na Plataforma Brasil) + folha de identificação do pesquisador. Devem ser informados como instituição coparticipante “aquela na qual haverá o desenvolvimento de alguma etapa da pesquisa. Esta é, portanto, uma instituição que participará do projeto, tal qual a proponente, apesar de não o ter proposto”. Nesse sentido, considerando que o recrutamento dos participantes ocorrerá a partir do ambulatório de infectologia do Hospital Giselda Trigueiro a mesma deve ser informada como instituição coparticipante nos documentos e deve apresentar carta de anuência. Da mesma forma, o Instituto de Medicina Tropical deverá ser informado como instituição coparticipante nos documentos citados anteriormente.
Resposta à pendência: Anexado documento de Folha de Identificação do pesquisador com as informações solicitadas e cadastro realizado na plataforma Brasil.
Análise da pendência: PENDÊNCIA PARCIALMENTE ATENDIDA. Inseridos dados das instituições coparticipantes na Plataforma Brasil e na folha de identificação do pesquisador, porém a carta de anuência apresentada não contempla as informações exigidas para este tipo de documento, a exemplo do título da pesquisa para o qual se está dando anuência, entre outros. Assim sugere-se utilizar o modelo proposto pelo CEP e que se encontra na página do CEP Central no endereço: http://www.cep.propesq.ufrn.br/ Observar que deve constar na folha de identificação do pesquisador o link para acesso ao currículo do pesquisador responsável. Preencher todos os campos do documento.
Pendência nº 02 - Termo de confidencialidade + Projeto cadastrado na Plataforma Brasil. A pesquisa prevê a participação de 3 pesquisadores, no entanto o documento só apresenta a assinatura da pesquisadora responsável e sua orientanda. Nesse sentindo TODOS os membros da pesquisa deverão ser devidamente cadastrados no projeto da Plataforma Brasil bem como devem assinar conjuntamente o termo de confidencialidade assegurando conhecimento e responsabilidade para com o compromisso ali assumido.
Resposta à pendência: As assinaturas foram encaminhadas no Termo de confidencialidade anexado, conforme solicitado. A outra pesquisadora também foi cadastrada na plataforma Brasil.
Análise da pendência: PENDÊNCIA ATENDIDA.
Pendência nº 03 - TCLE – Os riscos estimados ao longo do projeto e na plataforma Brasil não aparecem para o participante de pesquisa no TCLE. Nesse sentido, cabe reforçar que para o consentimento esclarecido do participante, a descrição na íntegra destes aspectos é essencial. Da mesma forma, devem ser descritas todas as etapas de avaliação a que o participante será submetido.

Resposta à pendência - As mudanças foram inseridas no TCLE.
Análise da pendência: PENDÊNCIA ATENDIDA.
Recomendação: verificar a formatação do documento. O espaço de rubrica encontra-se no meio da página.
Pendência nº 04 - Cronograma. O cronograma de pesquisa prevê coleta de dados em 19 de abril. Considerando que o projeto foi submetido em 15/04 deve-se rever esta proposta de início do projeto, uma vez que a estimativa para início não contemplava o processo de análise do CEP.
Resposta à pendência: A nova data prevê o início de coleta de dados em 1º de junho de 2021. A mudança foi realizada no cronograma do projeto.
Análise da pendência: PENDÊNCIA PARCIALMENTE ATENDIDA. Foi inserido novo cronograma, porém genérico, não há datas precisas para início do processo de coleta de dados junto aos participantes de pesquisa. Na Plataforma Brasil o período de início da pesquisa ainda se encontra em 19 de abril de 2021.
Pendência nº 05 - Orçamento – O orçamento não prevê os gastos com equipamentos e materiais previstos na pesquisa. Não informa sobre o dispositivo que ficará com os 10 participantes para realizar a intervenção aparelho POWERbreathe®. Quem irá custear a compra e disponibilização deste aparelho aos participantes? Não informa os diversos outros equipamentos e materiais que serão utilizados para avaliação dos participantes de pesquisa. Todos os equipamentos e materiais que serão necessários a realização da pesquisa deverá ser informada, mesmo que eles não sejam custeados pela pesquisadora responsável, mas pela proponente ou mesmo pela instituição coparticipante.
Resposta à pendência: As mudanças solicitadas foram realizadas no documento orçamento.
Análise da pendência: PENDÊNCIA PARCIALMENTE ATENDIDA. Foi inserido novo documento de orçamento, porém os dados cadastrados na Plataforma Brasil não foram modificados. Deve-se corrigir a informação em todos os documentos. Assim como deve deixar claro no projeto, na Plataforma Brasil que a pesquisa será custeada pelos pesquisadores e que os aparelhos serão fornecidos de forma gratuita para os participantes do estudo. Esta última informação deverá estar presente no TCLE.

Em análise ética realizada em 14 de maio de 2021 foram feitas as seguintes observações:
Pendência nº 01 - Inseridos dados das instituições coparticipantes na Plataforma Brasil e na folha de identificação do pesquisador, porém a carta de anuência apresentada não contempla as informações exigidas para este tipo de documento, a exemplo do título da pesquisa para o qual se

está dando anuência, entre outros. Assim sugere-se utilizar o modelo proposto pelo CEP e que se encontra na página do CEP Central no endereço:
http://www.cep.propesq.ufrn.br/. Observar que deve constar na folha de identificação do pesquisador o link para acesso ao currículo do pesquisador responsável. Preencher todos os campos do documento.
Resposta à pendência: Carta de anuência e folha de identificação do pesquisador anexadas.
Análise da pendência: PENDÊNCIA PARCIALMENTE ATENDIDA. A carta de anuência não informa qual a instituição está anuindo essa pesquisa. Uma vez que há a assinatura do responsável, mas não há informações sobre a instituição/empresa. Também faz referência a resolução 510/16, porém como trata-se de uma pesquisa na área biomédica deve-se fazer referência a resolução 466/12.
Folha de identificação do pesquisador OK.
Pendência nº 04 - Foi inserido novo cronograma, porém genérico, não há datas precisas para início do processo de coleta de dados junto aos participantes de pesquisa. Na Plataforma Brasil o período de início da pesquisa ainda se encontra em 19 de abril de 2021.
Resposta à pendência: Novo cronograma com mudanças solicitadas anexado e mudança de data realizada na Plataforma Brasil.
Análise da pendência: PENDÊNCIA ATENDIDA.
Pendência nº 05 - Foi inserido novo documento de orçamento, porém os dados cadastrados na Plataforma Brasil não foram modificados. Deve-se corrigir a informação em todos os documentos. Assim como deve deixar claro no projeto, na Plataforma Brasil que a pesquisa será custeada pelos pesquisadores e que os aparelhos serão fornecidos de forma gratuita para os participantes do estudo. Esta última informação deverá estar presente no TCLE.
Resposta à pendência: Mudanças realizadas. Novo projeto e TCLE anexados.
Análise da pendência: PENDÊNCIA NÃO ATENDIDA. NÃO LOCALIZADO NOVO TCLE ANEXADO. O último documento anexado data de 27 de abril na resposta à pendência anterior. Atentar para informar que a resolução é a 466/12 e não a 510/2016. Esta última é para pesquisas na área das ciências sociais e humanas, o que não é a característica desse trabalho. Deverá fazer essa correção no TCLE, na carta de anuência e projeto.
Sendo assim, observarmos a necessidade destes pequenos ajustes para que o projeto possa ser aprovado. São eles: 1. carta de anuência com os dados da instituição preenchidos e referência a resolução 466/12 - CNS e não a 510/16 - CNS; 2. TCLE modificado com informações sobre o acesso

gratuito ao aparelho que será utilizado pelos participantes e informando também a resolução 466/12 - CNS. Observar também a formatação.

Em análise ética realizada em 17 de maio de 2021, considerando a carta de resposta as pendências apresentadas, assim como o TCLE e carta de anuência modificados, o presente protocolo de pesquisa não apresenta óbices éticos a sua realização, estando, portanto, aprovado.

ORIENTAÇÕES PARA CONDUÇÃO DE PESQUISAS DURANTE A PANDEMIA DO SARS-COV2

A Conep, Comissão Nacional de Ética em Pesquisa, por meio do documento ORIENTAÇÕES PARA CONDUÇÃO DE PESQUISAS E ATIVIDADE DOS CEP DURANTE A PANDEMIA PROVOCADA PELO CORONAVÍRUS SARS-COV-2 (COVID-19), de 09 de maio de 2020, na página 02, orienta que:

"3.1. Aconselha-se a adoção de medidas para a prevenção e gerenciamento de todas as atividades de pesquisa, garantindo-se as ações primordiais à saúde, minimizando prejuízos e potenciais riscos, além de prover cuidado e preservar a integridade e assistência dos participantes e da equipe de pesquisa.

3.2. Em observância às dificuldades operacionais decorrentes de todas as medidas impostas pela pandemia do SARS-CoV-2 (Covid-19), é necessário zelar pelo melhor interesse do participante da pesquisa, mantendo -o informado sobre as modificações do protocolo de pesquisa que possam afetá-lo, principalmente se houver ajuste na condução do estudo, cronograma ou plano de trabalho.

3.3. Em virtude disso, enquanto perdurar o estado de emergência de saúde pública decorrente da Covid-19, recomenda-se que os CEP e toda a comunidade científica adotem, para a condução dos protocolos de pesquisa envolvendo seres humanos, as orientações da Conep, observando, ainda, no que couber, as diretrizes adotadas pela Agência Nacional de Vigilância Sanitária (Anvisa).

(...)

3.6. Caso sejam necessários a suspensão, interrupção ou o cancelamento da pesquisa, em decorrência dos riscos imprevisíveis aos participantes da pesquisa, por causas diretas ou indiretas,

caberá aos investigadores a submissão de notificação para apreciação do Sistema CEP/CONEP.”

Vale mencionar que, diante das recomendações governamentais e da Organização Mundial de Saúde, a UFRN, através da Portaria N° 452/2020-R, em 17 de março de 2020, no Art. 2º, “autoriza as atividades de extensão e de pesquisa que, por sua natureza, não impliquem aglomeração de pessoas, cabendo aos responsáveis avaliar as condições para cumprimento das recomendações das autoridades sanitárias.”

Por fim, recomendam-se ações que visem salvaguardar os envolvidos, participantes e pesquisadores, nas atividades de pesquisa, como, por exemplo, a aplicação de instrumentos (questionários, entrevistas, entre outros) de forma on-line e mais outras medidas que se julguem necessárias. Quaisquer dúvidas podem ser direcionadas aos nossos canais de comunicação: número móvel (84) 9.9193-6266, endereço de e-mail cepufrn@reitoria.ufrn.br ou formulário de contato do site <www.cep.ufrn.br>.

Considerações Finais a critério do CEP:
Em conformidade com a Resolução 466/12 e ou a Resolução 510/16 do Conselho Nacional de Saúde - CNS e Manual Operacional para Comitês de Ética - CONEP é da responsabilidade do pesquisador responsável:

1. Elaborar o Termo de Consentimento Livre e Esclarecido - TCLE em duas vias, rubricadas em todas as suas páginas e assinadas, ao seu término, pelo convidado a participar da pesquisa, ou por seu representante legal, assim como pelo pesquisador responsável, ou pela (s) pessoa (s) por ele delegada(s), devendo as páginas de assinatura estar na mesma folha (Res. 466/12 - CNS, item IV.5d);
2. Desenvolver o projeto conforme o delineado (Res. 466/12 - CNS, item XI.2c);
3. Apresentar ao CEP eventuais emendas ou extensões com justificativa (Manual Operacional para Comitês de Ética - CONEP, Brasília - 2007, p. 41);
4. Descontinuar o estudo somente após análise e manifestação, por parte do Sistema CEP/CONEP/CNS/MS que o aprovou, das razões dessa descontinuidade, a não ser em casos de justificada urgência em benefício de seus participantes (Res. 446/12 - CNS, item III.2u);
5. Elaborar e apresentar os relatórios parciais e finais (Res. 446/12 - CNS, item XI.2d);

6. Manter os dados da pesquisa em arquivo, físico ou digital, sob sua guarda e responsabilidade, por um período de 5 anos após o término da pesquisa (Res. 446/12 - CNS, item XI.2f);
7. Encaminhar os resultados da pesquisa para publicação, com os devidos créditos aos pesquisadores associados e ao pessoal técnico integrante do projeto (Res. 446/12 - CNS, item XI.2g) e,
8. Justificar fundamentadamente, perante o CEP ou a CONEP, interrupção do projeto ou não publicação dos resultados (Res. 446/12 - CNS, item XI.2h).

Este parecer foi elaborado baseado nos documentos abaixo relacionados:

Tipo Documento	Arquivo	Postagem	Autor	Situação
Informações Básicas do Projeto	PB_INFORMAÇÕES_BÁSICAS_DO_PROJETO_1719549.pdf	17/05/2021 11:02:13		Aceito
Outros	Carta_de_Respostas_s_Pendncias.docx	17/05/2021 11:01:53	Patrícia Angélica de Miranda Silva Nogueira	Aceito
Outros	Carta_de_Anuencia_modificado.pdf	16/05/2021 23:43:28	Patrícia Angélica de Miranda Silva Nogueira	Aceito
TCLE / Termos de Assentimento / Justificativa de Ausência	TCLE_modificado.pdf	16/05/2021 23:42:52	Patrícia Angélica de Miranda Silva Nogueira	Aceito
Projeto Detalhado / Brochura Investigador	Projeto_Covid19_Completo_modificado.docx	12/05/2021 15:54:11	Patrícia Angélica de Miranda Silva Nogueira	Aceito
Outros	Folha_de_identificao_do_pesquisador_modificado.pdf	12/05/2021 15:49:40	Patrícia Angélica de Miranda Silva Nogueira	Aceito
Cronograma	Cronograma_modificado.pdf	12/05/2021 15:47:11	Patrícia Angélica de Miranda Silva Nogueira	Aceito
Orçamento	Orcamento_modificado.pdf	28/04/2021 09:16:33	Patrícia Angélica de Miranda Silva Nogueira	Aceito
Declaração de Pesquisadores	Termo_de_confidencialidade_modificado.pdf	27/04/2021 21:26:05	Patrícia Angélica de Miranda Silva Nogueira	Aceito
Declaração de concordância	Declarao_de_Compromisso.pdf	17/03/2021 18:58:03	Patrícia Angélica de Miranda Silva Nogueira	Aceito
Outros	Carta_anuencia_mag.pdf	17/03/2021	Patrícia Angélica de	Aceito

Outros	Carta_anuencia_mag.pdf	18:57:06	Miranda Silva Nogueira	Aceito
Folha de Rosto	Folha_Rosto_assinada.pdf	17/03/2021 18:52:52	Patrícia Angélica de Miranda Silva Nogueira	Aceito

Situação do Parecer:
Aprovado

Necessita Apreciação da CONEP:
Não

NATAL, 18 de Maio de 2021

Assinado por:
PAULA FERNANDA BRANDÃO BATISTA DOS SANTOS (Coordenador(a))

Endereço: Universidade Federal do Rio Grande do Norte ¿ Campus Central, s/n.
Bairro: Lagoa Nova **CEP:** 59.078-970
UF: RN **Município:** NATAL
Telefone: (84)3215-3135 **Fax:** (84)99193-6266 **E-mail:** cepufrn@reitoria.ufrn.br
