# Supplementary material for: Efficacy of home-based inspiratory muscle training in patients post-covid-19: Protocol for a randomized clinical trial
Source: PLoS One. 2023 May 4;18(5):e0279310. doi: 10.1371/journal.pone.0279310 (PMC10159136; doi:10.1371/journal.pone.0279310)
Supplement: S2 File — (PDF) [file pone.0279310.s003.pdf]

OPINION SUBSTANTIATED CEP

RESEARCH PROJECT DATA

Research Title: Efficacy of home inspiratory muscle training in post-covid-19 patients: randomized clinical trial.

Researcher: Patrícia Angélica de Miranda Silva Nogueira

Thematic Area:

Version: 4

CAAE: 45575421.7.0000.5537

Institution Proponent: Department of Physiotherapy

Main Sponsor: Own Financing

OPINION DATA

Opinion Number: 4,719,458

Project Presentation:

PhD project of the graduate program in Physiotherapy, does not provide for a co-participant institution. It aims to "Evaluate the effectiveness of a home inspiratory muscle training protocol in improving respiratory muscle strength, dyspnea and quality of life of post-Covid-19 patients". Conducting the research from April 2021-2023. Sample number to be defined. It will recruit participants from their referral by the Infectious Diseases Outpatient Clinic of the Giselda Trigueiro Hospital. This is an experimental intervention study where the subjects included in the research will go through three moments of evaluations: Pre-training (Initial), Post-Training (6 weeks) and Retention Test (24 weeks). After recruitment, participants will be invited to attend the Institute of Tropical Medicine to perform an initial evaluation by a previously trained and blind evaluator for the intervention allocation group and will include anamnesis and physical examination, with measurement of vital signs, measurements anthropometric, evaluation of lung volumes, respiratory muscle strength, peripheral muscle strength, quality of life, anxiety and depression, functional status and 6-minute walk test. After the initial evaluation, all volunteers will receive a POWERbreathe device® (POWERbreathe®, HaB Ltd, Southam, UK) for training, and will be guided individually on how to use it and how to carry out the protocol. They will hold an experimental session for familiarization with the device that will not be considered for

analysis. Every three days volunteers will receive a telephone call from researcher 2 who will not participate in the evaluation to confirm whether the exercise with POWERbreathe® was being performed adequately in the frequency and load oriented and if there was any doubt regarding the protocol. At the end of each week participants will receive a video connection from researcher 2 to perform the adjustment on the device according to the weekly load progression of G1. All evaluation moments (pre-training, post-training and retention test) will be performed by a single evaluator (Evaluator 1) – who will not know in which group the subject will be allocated – and recorded in the evaluation form developed for the project. A second pesquisador (Evaluator 2) will be responsible for applying the training protocols to the subjects. Inclusion Criteria: Subjects of both sexes, sedentary, diagnosed with COVID-19 confirmed by RT-PCR, aged over 18 years and without any basic respiratory disease , with adequate cognitive status defined through the Mini Mental State Examination (MMSE) (ANNEX) will be considered as inclusion criteria. 1) and reduction of respiratory muscle strength, defined through the evaluation of MIP., of a relationship with the following prediction equations: men (153.3
- 0.8 x age) and women (110.4 - 0.49 x age) and considering the lower threshold of normality with error estimation standard (EPE) for men of 17.3 and women of 9.1 (NEDER et al., 1999). Exclusion Criteria: Subjects with some condition that makes it impossible to perform evaluations and protocols, complications that justify the interruption of data collection, such as syncope, severe chest pain, cough with those who request exit from the study and who have adverse effects such as hospitalization for sharpening the clinical picture. Data Analysis Methodology: For data analysis will be used the software SPSS (Statistical Package for the Social Sciences) version 22.0 for Windows. The normality test will be performed according to the number of volunteer participants of the research. Therefore, the ShapiroWilk normality test or the Kolmorov-Smirnov normality test for the desired variables may be used. The variables that present non-parametric distribution will be compared through the Wilcoxon (intragroup analysis) and Mann-Whitney (intergroup analysis) tests and when the distribution occurs of parametric forma the anova two-way test will be used. When there is significant difference, Dunn's post hoc test will be applied in order to locate the differences. In the descriptive analysis, a characterization of the studied population will be performed, through the obtaining of means and standard deviations for variables of normal or median distribution and interquartile interval (25%-75%) for variables of asymmetric distribution. To minimize a possible type I error, the significance level of 5% will be established. The power of the study and the size of the effect will be

exposed in the main results of the study.

Research Objective:

Primary Objective:

Evaluate the efficacy of a home inspiratory muscle training protocol in improving respiratory muscle strength, dyspnea and quality of life of post-Covid-19 patients.

Secondary Objective:

Evaluate after IMT the influence of:

- Pulmonary function;
- Perception of exertion and dyspnea;
- Exercise tolerance;
- Quality of life;
- Peripheral muscle strength;
- Anxiety and depression;
- Functional status.

Risk and Benefit Assessment:

The risks and benefits were thus estimated in the project:

Risks:

This research presents minimal risks to participants. These may feel constrained

during the interview, considering the nature of the content of some questionnaires, which he may refuse to answer, or because of the difficulty in performing any specific evaluation. They may present some discomforts such as headache, vertigo, nausea, blood pressure oscillation and even a small imbalance due to the requirement of certain physical tests and training, however the evaluators will use contraindication criteria or interruptions of the tests when necessary, to minimize the risks to the health of the participants. The participant may refuse to perform any procedure at any time, without prejudice to him. In addition, they may also present concerns about the confidentiality of the data collected and the signature they must make in the TCLE. The researcher will assist
them clarifying all existing doubts and informing participants that the data collected will be used only for academic purposes, and that there will be no disclosure of them.

Benefits:

The results obtained with the development of this research can guarantee the possibility of expanding interventions related to pulmonary rehabilitation in post-covid-19 patients, through effective, accessible, safe and low-cost training. The information raised may also contribute to the screening of patients after the period of acute infection, contributing to the understanding of about this new theme, the development and evaluation of the effectiveness of interventions aimed at improving the health of this population. However, this same information is not included in the ETS. There is an underestimation of these risks.

Comments and Considerations about Research:

The research project is well structured, presenting the necessary elements for a doctoral work. It intends to contribute to the rehabilitation of patients who had covid-19, through respiratory muscle training. The proposal, however, requires some adjustments to meet the ethical regulations that deal with research involving human beings and that are described in the item "Conclusions or pending and List of inadequacies".

Considerations on the Mandatory Submission Terms:

1) Cover sheet.
2) Statement of ethical commitment not to start the research.

3) Confidentiality agreement. It is signed only by the researcher in charge and the doctoral program. As in the study design, 3 researchers should be involved, it should be clarified who these members of the research team are and all should sign this gift, as well as be included in the Project registered in the Brazil Platform.

4) Schedule.

5) Budget – does not inform about the device that will stay with the 10 participants to perform the intervention POWERbreathe device®. Who will pay for the purchase and availability of this device to the participants? It does not inform the various other equipment and materials that will be used for evaluation of research participants. All teams and materials that will be necessary to carry out the research must be informed, even if they are not funded by the researcher responsible, but by the proponent or even the co-participant institution.

6) Project registered in Plataforma Brasil. (PB Project).

7) Researcher identification sheet. You need to identify the co-participant institution - Giselda Trigueiro Hospital. Also register on the Brazil Platform.

8) Letter of withdrawal from the Institute of Tropical Medicine.

9) does not describe to satisfaction the estimated risks to the intervention. It does not describe all the assessments that participants will pass. There are around 14 evaluations, and the TCLE describes only a few of these. It should also include the application of depression and anxiety scala, post-covid-19 functional status scale, among others also not described.

Recommendations:

Dear researcher, if, due to the pandemic corona virus (COVID-19) the schedule presented undergoing change, it is recommended, when establishing updated dates, the sending of a new schedule to the Central CEP / UFRN, in the form of notification of the type "Communication of Project Beginning".

Conclusions or Pending and List of Inadequacies:

According to ethical analysis carried out considering the provisions of resolution 466/2012 - CNS that deals with research involving human beings, the following pending issues were observed:

PENDING 01 - Co-participating institutions. Documents: PB Project (registered in the Platform

Brazil) + identification sheet of the researcher. They should be informed as a co-participant institution "one in which there will be the development of some stage of the research. This is, therefore, an institution that will participate in the project, just like the proponent, even though it has not proposed it." In this sense, considering that the recruitment of participants will take place from the infectious diseases outpatient clinic of the Giselda Trigueiro Hospital, it should be informed as a co-participant institution in the documents and must present a letter of withdrawal. Similarly, the Institute of Tropical Medicine should be informed as a co-participant institution in the documents mentioned above.

PENDING 02 - Confidentiality agreement + Project registered in the Brazil Platform. The research foresees the participation of 3 researchers, however the document only presents the signature of the responsible researcher and her guidance. In this sense ALL research members should be duly registered in the Project of the Brazil Platform as well as must jointly sign the confidentiality agreement ensuring knowledge and responsibility for the commitment made there. PENDING 03 - TCLE - The risks estimated throughout the project and on the Brazil platform do not appear for the research participant in the TCLE. In this sense, it should be reinforced that for the informed consent of the participant, the full description of these aspects is essential. In the same way, all the evaluation steps to which the participant will be submitted should be described.

PENDING 04 - Schedule. The research schedule foresees data collection on April 19. Considering that the project was submitted on 15/04, this proposal should be reviewed at the beginning of the project, since the initial estimate did not include the cep analysis process.

PENDING 05 - Budget - The budget does not provide for expenditure on equipment and materials foreseen in the research. It does not inform about the dispositivo that will stay with the 10 participants to perform the intervention POWERbreathe apparatus®. Who will pay for the purchase and availability of this device to the participants? It does not inform the various other equipment and materials that will be used for evaluation of research participants. All equipment and materials that will be necessary to carry out the research must be informed, even if they are not funded by the researcher responsible, but by the proponent or even by the coparticipante institution.

In an ethical analysis carried out on May 5, 2021, considering the new documents included and the letter of reply the pending evaluations were made:

Pending no. 01 - Co-participating institutions. Documents: PB Project (registered in the Brazil Platform) + data sheet de identification of the researcher. They should be informed as a co-participant institution "one in which there will be the development of some stage of the research. This is, therefore, an institution that will participate in the project, just like the proponent, even though it has not proposed it." In this sense, considering that the recruitment of participants will take place from the infectious diseases outpatient clinic of the Giselda Trigueiro Hospital, it should be informed as a co-participant institution in the documents and must present a letter of withdrawal. Similarly, the Institute of Tropical Medicine should be informed as a co-participant institution in the documents mentioned above.
Response to the pending: Attached document of Identification Sheet of the researcher with the information solicitadas and registration made on the Platform Brazil.
Pending analysis: PENDING PARTIALLY MET. Inserted data from the institutions co-participating in the Brazil Platform and in the researcher's identification sheet, but the letter of agreement presented does not include the information required for this type of document, such as the title of the research for which the agreement is being given, among others. Thus, it is suggested to use the model proposed by the CEP and which is on the Central ZIP Code page at the endereço: http://www.cep.propesq.ufrn.br/ Note that the link to access the curriculum of the responsible researcher should be included in the researcher's identification sheet. Fill in all the fields of the document.
Pending no. 02 - Confidentiality agreement + Project registered in the Brazil Platform. The research foresees the participation of 3 researchers , however the document only presents the signature of the responsible researcher and her guidance. In this sense ALL research members should be duly registered in the Project of the Brazil Platform as well as must jointly sign the confidentiality agreement ensuring knowledge and responsibility for the commitment made there. Response to the pending: Signatures were forwarded in the attached Confidentiality Agreement as requested. The other researcher was also registered on the Brazil platform.
Pending analysis: PENDING SERVED.
Pending No. 03 - TCLE - The risks estimated throughout the project and on the Brazil platform do not appear for the research participant in the TCLE. In this sense, it should be reinforced that for the informed consent of the participant, the full description of these expectations is essential. Similarly, all the evaluation steps to which the participant will be submitted should be described.

Response to the pending - The changes were inserted in the
TCLE. Pending analysis: PENDING SERVED.
Recommendation: Check the formatting of the document. The rubric space is in the middle of the page.
Pending no. 04 - Schedule. The pesquisa schedule provides for data collection on April 19. Considering that the project was submitted on 15/04, this proposal should be reviewed at the beginning of the project, since the initial estimate did not include the cep analysis process.
Response to the pending: The new data foresees the start of data collection on June 1, 2021. The change was made in the project schedule.
Pending analysis: PENDING PARTIALLY MET. A new schedule was inserted, but generic, there are no precise dates for the beginning of the data collection process with the research participants. In Plataforma Brasil the period of beginning of the research is still on April 19, 2021. Pending No. 05 - Budget - The budget does not provide for expenditure on equipment and materials foreseen in the research. It does not inform about the device that will stay with the 10 participants to perform the intervention POWERbreathe device®. Who will pay for the purchase and availability of this device to the participants? It does not inform the various other equipment and materials that will be used for evaluation of research participants. All equipment and materials that will be necessary to carry out the research must be informed, even if they are not funded by the researcher responsible, but by the proponent or even by the co-participant institution.
Response to the pending: The requested changes were made in the budget document.
Pending analysis: PENDING PARTIALLY MET. A new budget document was inserted, but the data signed in the Brazil Platform were not modified. The information must be corrected in all documents. As you should make clear in the project, in plataforma Brasil that the research will be funded by the researchers and that the devices will be provided free of charge for the study participants. This last information should be present in the ETS.

In an ethical analysis conducted on May 14, 2021, the following observations were made:
Pending No. 01 - Data from the co-participating institutions inserted in the Brazil Platform and in the researcher's identification sheet, but the letter of approval presented does not include the information required for this type of document, such as the title of the research for which the

is giving an agreement, among others. Thus it is suggested to use the model proposed by the CEP and which is on the Central ZIP Code page at:
http://www.cep.propesq.ufrn.br/. Note that the researcher's identification sheet should be linked to access to the curriculum of the responsible researcher. Fill in all the fields of the document.
Response to the pending: Letter of withdrawal and identification sheet of the researcher attached.
Pending analysis: PENDING PARTIALLY MET. The letter of withdrawal does not inform which institution is analysing this research. Since there is the signature of the responsible, but there is no information about the institution /company. It also refers to the 510/16 resolution, but as it is a research in the biomedical area, it is appropriate to refer to resolution 466/12.
Researcher ID sheet OK.
Pending no. 04 - A new schedule was inserted, but generic, there are no precise dates to start the data collection process with the research participants. In Plataforma Brasil the period of beginning of the research is still on April 19 , 2021.
Response to the pending: New schedule with requested changes attached and change of date realizada in the Brazil Platform.
Pending analysis: PENDING SERVED.
Pending no. 05 - A new budget document was inserted, but the data registered in the Brazil Platform were not modified. The information must be corrected in all documents. As you should make clear in the project, in plataforma Brasil that the research will be funded by the researchers and that the devices will be provided free of charge to the study participants. This last information should be present in the ETS.
Pending: Changes made. New design and TCLE attached.
Pending analysis: PENDING NOT MET. NOT LOCATED NEW TCLE ATTACHED. Or
last attached document dated April 27 in response to the previous pending. Be very sure that the resolution is 466/12 and not 510/2016. The latter is for research in the area of social sciences and humanities, which is not the characteristic of this work. It shall make this correction in the ETS, in the letter of consent and project.
Therefore, we observe the need for these small adjustments so that the project can be approved. They are:
1. letter of withdrawal with the data of the institution filled out and reference to resolution 466/12 - CNS and not to 510/16 - CNS; 2. Modified TCLE with access information

device that will be used by the participants and also informing the resolution 466/12 - CNS. Also note the formatting.

In an ethical analysis carried out on May 17, 2021, considering the letter of response presented, as well as the modified TCLE and letter of consent, this research protocol does not present ethical obstacles to its realization, being, portanto, approved.

GUIDELINES FOR CONDUCTING RESEARCH DURING THE PANDEMIC OF SARS-COV2

Conep, National Research Ethics Commission, through the document GUIDELINES FOR CONDUCTING RESEARCH AND ACTIVITY OF CEP DURING THE PANDEMIC CAUSED BY THE CORONAVIRUS SARS-COV-2 (COVID-19), of May 9, 2020, on page 02, advises that:

"3.1. It is advisable to adopt measures for the prevention and management of all research activities, ensuring the primary actions to health, minimizing losses and potential risks, in addition to providing care and preserving the integrity and assistance of participants and the research team.

3.2. In compliance with the operational difficulties arising from all the measures imposed by the SARS-CoV-2 pandemic (Covid-19), it is necessary to ensure the best interest of the research participant, maintaining -informed him about the modifications of the pesquisa protocol that may affect him, especially if there is adjustment in the conduct of the study, schedule or work plan.

3.3. As a result, as long as the state of public health emergency resulting from Covid-19 persists, it is recommended that the CEP and the entire scientific community take, in order to conduct research protocols involving human beings, conep's guidelines, also observing in what guidelines adopted by the National Health Surveillance Agency (Anvisa).

(...)

3.6. If the suspension, interruption or cancellation of the research is necessary, due to the risks unpredictable to the participantes of the research, for direct or indirect causes,

Address: Federal University of Rio Grande do Norte ¿ Central Campus, s/n.
Neighborhood: New Lagoon **POC** 59.078-970
Municipality:
Telephon (84)3215-3135 **Fax:** (84)99193-6266 **Email:** cepufrn@reitoria.ufrn.br

researchers will be responsible for the submission of notification for consideration of the CEP/CONEP System."

It is worth mentioning that, in view of the recommendations of the government and the World Health Organization, UFRN, through Ordinance No. 452/2020-R, on March 17, 2020, in Art. 2, "authorizes the extension and research activities that, by their nature, do not imply agglomeration of people, and it is up to the responsible to evaluate the conditions for compliance with the recommendations of the health authorities."

Finally, actions are recommended to safeguard those involved, participants and researchers, in research activities, such as the application of instruments (questionnaires, interviews, among others) online and other mediated that are deemed necessary. Any questions may be directed to our communication channels: mobile number (84) 9.9193-6266, cepufrn@reitoria.ufrn.br email address or contact form of the <www.cep.ufrn.br>.

Final Considerations at cep's discretion:

In accordance with Resolution 466/12 and or Resolution 510/16 of the National Health Council - CNS and Operational Manual for Ethics Committees - CONEP is the responsibility of the responsible researcher:

1. Prepare the Free and Informed Consent Form - Informed Consent in two ways, initialed on all its pages and signed, at its end, by the invitee to participate in the research, or by his legal representative, as well as by the responsible researcher, or by the person(s) delegated by him, and the pages signature be on the same sheet (Res. 466/12 - CNS, item IV.5d);
2. Develop the project as outlined (Res. 466/12 - CNS, item XI.2c);
3. Submit to the CEP any amendments or extensions with justification (Operational Manual for Ethics Committees - CONEP, Brasília - 2007, p. 41);
4. Discontinue the study only after analysis and manifestation by the CEP/CONEP/CNS/MS System that approved it, the reasons for this discontinuity, unless in cases of justified urgency for the benefit of its participants (Res. 446/12 - CNS, item III.2u);
5. Prepare and present partial and final reports (Res. 446/12 - CNS, item XI.2d);

6. Keep the research data on file, physical or digital, under your custody and responsibility, for a period of 5 years after the end of the research (Res. 446/12 - CNS, item XI.2f);
7. Forward the results of the research for publication, with due credit to the associated researchers and technical staff integral to the project (Res. 446/12 - CNS, item XI.2g) and,
8. Justify reasoned, before the CEP or CONEP, interruption of the project or non-publication of the results (Res. 446/12 - CNS, item XI.2h).

This opinion was prepared based on the documents listed below:

Document Type	Archive	Post	Author	Situation
Basic Information of the Project	PB_INFORMAÇÕES_BÁSICAS_DO_PROJETO_1719549.pdf	17/05/2021 11:02:13		Accepted
Other	Carta_de_Respostas_s_Pendncias.docx	17/05/2021 11:01:53	Patricia Angelica of Miranda Silva Walnut	Accepted
Other	Carta_de_Anuencia_modificado.pdf	16/05/2021 23:43:28	Patricia Angelica of Miranda Silva Walnut	Accepted
TCLE / Terms of Nod / Justification of Absence	TCLE_modificado.pdf	16/05/2021 23:42:52	Patricia Angelica of Miranda Silva Walnut	Accepted
Detailed Project / Brochure Researcher	Projeto_Covid19_Completo_modificado.docx	12/05/2021 15:54:11	Patricia Angelica of Miranda Silva Walnut	Accepted
Other	Folha_de_identificao_do_pesquisador_modified.pdf	12/05/2021 15:49:40	Patricia Angelica of Miranda Silva Walnut	Accepted
Schedule	Cronograma_modificado.pdf	12/05/2021 15:47:11	Patricia Angelica of Miranda Silva Walnut	Accepted
Budget	Orcamento_modificado.pdf	28/04/2021 09:16:33	Patricia Angelica of Miranda Silva Walnut	Accepted
Declaration of Researchers	Termo_de_confidencialidade_modificado.pdf	27/04/2021 21:26:05	Patricia Angelica of Miranda Silva	Accepted

			Walnut	
Declaration of agreement	Declarao_de_Compromisso.pdf	17/03/2021 18:58:03	Patricia Angelica of Miranda Silva Walnut	Accepted
Other	Carta_anuencia_mag.pdf	17/03/2021	Patricia Angelica of	Accepted

Address: Federal University of Rio Grande do Norte ¿ Central Campus, s/n.
Neighborhood: New Lagoon **POC** 59.078-970
Municipality:
Telephon (84)3215-3135 **Fax:** (84)99193-6266 **Email:** cepufrn@reitoria.ufrn.br

Other	Carta_anuencia_mag.pdf	18:57:06	Miranda Silva Walnut	Accepted
Cover Sheet	Folha_Rosto_assinada.pdf	17/03/2021 18:52:52	Patricia Angelica of Miranda Silva Walnut	Accepted

Status of the Opinion:
Approved

ConeP Needs Appreciation:
No

NATAL, 18 May 2021

Signed by:
PAULA FERNANDA BRANDÃO BATISTA DOS SANTOS
(Coordinator)

Address: Federal University of Rio Grande do Norte ¿ Central Campus, s/n.
Neighborhood: New Lagoon **POC** 59.078-970
Municipality:
Telephon (84)3215-3135 **Fax:** (84)99193-6266 **Email:** cepufrn@reitoria.ufrn.br
