# Supplementary material for: Efficacy of home-based inspiratory muscle training in patients post-covid-19: Protocol for a randomized clinical trial
Source: PLoS One. 2023 May 4;18(5):e0279310. doi: 10.1371/journal.pone.0279310 (PMC10159136; doi:10.1371/journal.pone.0279310)
Supplement: S3 File — (PDF) [file pone.0279310.s004.pdf]

UNIVERSIDADE FEDERAL DO RIO GRANDE DO NORTE
CENTRO DE CIÊNCIAS DA SAÚDE
PROGRAMA DE PÓS GRADUAÇÃO EM FISIOTERAPIA

EFICÁCIA DO TREINAMENTO MUSCULAR INSPIRATÓRIO DOMICILIAR EM PACIENTES PÓS-COVID-19: ENSAIO CLÍNICO RANDOMIZADO.

GABRIELY AZEVÊDO GONÇALO SILVA

NATAL/RN
2022

GABRIELY AZEVÊDO GONÇALO SILVA

EFICÁCIA DO TREINAMENTO MUSCULAR INSPIRATÓRIO DOMICILIAR EM PACIENTES PÓS-COVID-19: ENSAIO CLÍNICO RANDOMIZADO.

Projeto de Pesquisa apresentado à Pós-Graduação em Fisioterapia da Universidade Federal do Rio Grande do Norte para apreciação do Comitê de Ética em pesquisa com seres humanos.

Área de concentração: Avaliação e intervenção em Fisioterapia nos sistemas cardiovascular e respiratório.

Orientadora: Profa. Dra. Patrícia Angélica de Miranda Silva Nogueira.

NATAL/RN

2022

RESUMO

INTRODUÇÃO: A doença por coronavírus 2019 (COVID-19), causada pela Síndrome Respiratória Aguda Grave (SRAG) do coronavírus 2 (SARS-CoV-2) ultrapassou o número global de 119.603.761 casos, com mais de 2.649.722 mortes relatadas. Existem evidências suficientes para uma possível Síndrome pós-covid-19, designando sequelas com sintomas persistentes. O treinamento muscular respiratório melhora a força muscular respiratória, a capacidade de exercício, a espessura do músculo diafragma e a dispneia em várias populações de paciente, especialmente naqueles com maior redução de força muscular respiratória basal. **OBJETIVO:** Avaliar a eficácia de um protocolo de treinamento muscular inspiratório domiciliar na melhora da força muscular respiratória, dispneia e qualidade de vida de pacientes pós-Covid-19. **MATERIAS E MÉTODOS:** Trata-se de um ensaio clínico, controlado, randomizado e cego, que será realizado no Instituto de Medicina Tropical da Universidade Federal do Rio Grande do Norte. O tamanho amostral será realizado através do software GPower versão 3.1.9.2 (Kiel, Alemanha) para Windows e será estabelecida após a realização de estudo piloto com 5 participantes em cada grupo (total de 10 sujeitos) para um teste ANOVA two-way hipotético, utilizando-se a variável principal a Pressão Inspiratória Máxima (PImáx.). Os sujeitos incluídos na pesquisa passarão por três momentos de avaliações: Pré-treinamento (Inicial), Pós-Treinamento (6 semanas) e Teste de Retenção (24 semanas) para ficha de avaliação clínica, medidas antropométricas, força muscular respiratória, volumes e capacidades pulmonares, dispneia, percepção de esforço e fadiga, força de preensão manual, teste de caminhada de seis minutos, ansiedade e depressão, estado funcional pós-covid. Após a avaliação inicial, todos os voluntários receberão um aparelho POWERbreathe® (POWERbreathe®, HaB Ltd, Southam, UK), para realização do treinamento.

Palavras-chave: espirometria, covid-19, força muscular respiratória.

SUMÁRIO

1 INTRODUÇÃO 5
1.1 JUSTIFICATIVA...8
1.2 OBJETIVOS 9
1.2.1 Objetivo primário..9
1.2.2 Objetivos secundários 9
1.3 HIPÓTESE..10
2 MATERIAIS E MÉTODOS 10
2.1 TIPO DE PESQUISA 10
2.2 POPULAÇÃO DA PESQUISA..10
2.3 TAMANHO DA AMOSTRA...10
2.4 CRITÉRIOS DE INCLUSÃO E EXCLUSÃO 10
2.5 PROCEDIMENTOS PARA SELEÇÃO DOS SUJEITOS.................................11
2.6 ASPECTOS ÉTICOS 11
2.7 DESENHO DO ESTUDO...11
2.8 PROCEDIMENTO DE OBTENÇÃO DOS DADOS **.......................................12**
2.8.1 Ficha de avaliação clínica 12
2.8.2 Medidas antropométricas 13
2.8.3 Força muscular respiratória..13
2.8.4 Volumes e capacidades pulmonares..13
2.8.5 Avaliação da dispneia..15
2.8.6 Percepção de esforço e fadiga 15
2.8.7 Força de preensão manual..15
2.8.9 Teste da caminhada de seis minutos 16
2.8.10 Medical Outcomes Study 36-Item Short Health Form Survey (SF-36) 17
2.8.11 International Physical Activity Questionnaire 17
2.8.12 Escala de Ansiedade e Depressão..18
2.8.13 Escala de estado funcional pós-COVID-19 18
2.8.14 Avaliação dos efeitos adversos e adesão 18
2.9 PROTOCOLO DE INTERVENÇÃO DA PESQUISA.. 19
2.10 RISCOS...19
2.11 BENEFÍCIOS................................. 20

2.12 ANÁLISE DOS DADOS 20
3 DESFECHOS 20
3.1 DESFECHO PRIMÁRIO 20
3.2 DESFECHOS SECUNDÁRIOS 20
4 CRONOGRAMA..21
5 ORÇAMENTO 22
REFERÊNCIAS 23
ANEXOS..28
APÊNDICES 45

1 INTRODUÇÃO

A doença por coronavírus 2019 (COVID-19), causada pela Síndrome Respiratória Aguda Grave (SRAG) do coronavírus 2 (SARS-CoV-2) ultrapassou o número global de 394.381.395 casos confirmados até 7 de fevereiro de 2022, com mais de 5.735.170 mortes relatadas (OMS, 2022). Pesquisadores estimaram que cerca de 81% dos casos confirmados de COVID-19 são leves, com um período de recuperação usual de 2 semanas, 14% progredindo para pneumonia grave e 5% desenvolvendo SRAG, sepse e / ou falência de órgãos multissistêmicos (WU; MCGOOGAN, 2020), sendo os idosos mais gravemente afetados e apresentando uma letalidade de 8-15% (ZHOU et al., 2020).

Os pulmões de uma pessoa infectada são os órgãos mais afetados porque o vírus acessa as células hospedeiras por meio da Enzima Conversora de Angiotensina 2 (ACE2), que é mais abundante nas células alveolares do tipo II. Assim, as manifestações respiratórias como tosse, produção de expectoração e falta de ar continuam sendo os sintomas mais comuns, após a febre (HUANG et al., 2020). Apesar disso, manifestações extra respiratórias como as cardiovasculares, gastrointestinais, neurológicas, hepáticas, renais, cutâneas e hematológicas também podem ser observadas nos pacientes, contribuindo para complicações e repercussões diversas após a fase aguda da doença (LAI et al., 2020).

A avaliação de alguns pacientes com uma média de 60 dias após o início de primeiros sintomas mostrou que, apenas 12,6% estavam completamente livres de qualquer sintoma relacionado ao COVID-19, enquanto 32% tinham 1 ou 2 sintomas e 55% tinham 3 ou mais. Piora na qualidade de vida foi observada em 44,1% dos pacientes. Grande parte dos indivíduos ainda relatou fadiga (53,1%), dispneia (43,4%), dores nas articulações (27,3%) e dores no peito (21,7%). Este estudo evidenciou que dos pacientes que se recuperaram do COVID-19, 87,4% relataram persistência de pelo menos 1 sintoma, sendo a fadiga e dispneia os principais (CARFI et al., 2020).

Uma revisão sistemática que buscou comparar a função física e os resultados de aptidão em pessoas infectadas com Coronavírus relacionado à SARS-CoV observou que a função física e a força física são prejudicadas após a infecção, e as deficiências podem persistir até 1 a 2 anos. Considerando as semelhanças na patologia e apresentação clínica da SARS-CoV e COVID-19, é provável que os pacientes com COVID-19 apresentem deficiências semelhantes na função física (ROONEY; WEBSTER; PAUL, 2020). Essas

descobertas podem servir para compreender as deficiências potenciais e as necessidades de reabilitação de pessoas em recuperação do surto atual de COVID-19.

Estudo de acompanhamento de 1 ano de 96 pacientes pós-COVID-19 que tiveram atendimento ambulatorial ou internação hospitalar mostrou que aos 12 meses os sintomas mais frequentemente relatados foram capacidade de exercício reduzida (56,3%), fadiga (53,1%), dispneia (37,5%), dificuldades de concentração (39,6%), problemas de memória (32,3%) e de sono (26%) (SEEBLE et al., 2021).

A síndrome pós-COVID-19 pode estar associada à inflamação sistêmica subclínica crônica, como pode ser observado no processo de envelhecimento. Essa “inflamação” pode ter o potencial de piorar as comorbidades existentes e exacerbar os problemas relacionados à idade. Estudos que avaliaram o acompanhamento tomográfico de pacientes que sobreviveram a SARS e MERS mostram a persistência de alterações sequelares pulmonares em uma grande proporção dos casos. Uma análise com o seguimento de pacientes por 15 anos, mostrou que 38% dos pacientes persistiam com opacidades em vidro fosco ou consolidações lineares no acompanhamento (ZHANG et al., 2020). Na avaliação de pacientes um ano após infecção pelo MERS-CoV, alterações tomográficas sequelares foram observadas em 63% dos casos de pneumonia leve e 95% dos casos de pneumonia grave (PARK et al., 2018). No caso de SARS, consequências duradouras na forma de distúrbio ventilatório restritivo ou doença pulmonar devem ser esperadas em pelo menos 25% dos sobreviventes (BURNHAM et al., 2013).

Pacientes com SARs-COV apresentaram um padrão restritivo leve ou moderado consistente com fraqueza muscular em 6% a 20% dos indivíduos no teste de função pulmonar realizados 6 a 8 semanas após a alta hospitalar, com prejuízo persistente da função pulmonar em cerca de um terço dos pacientes em 1 ano de acompanhamento. O estado de saúde desses sobreviventes da SARS também foi significativamente pior em comparação com a população saudável, achados anormais na radiografia de tórax, bem como reduções persistentes na capacidade de exercício em 12 meses e déficit no desempenho musculoesquelético e QV. Considerando essas informações, as complicações respiratórias devem ser consideradas em pacientes pós-COVID-19, pois os pacientes podem apresentar algum grau de comprometimento e limitação funcional, devido à diminuição da função respiratória (BARKER-DAVIES et al., 2020).

Dados iniciais de um acompanhamento tomográfico de 59 pacientes pós-COVID-19 um mês após a alta hospitalar, mostraram que 39% apresentava sinais de fibrose residual, definida como bandas parenquimatosas, interfaces irregulares e bronquiectasias.

Estes tinham idade mais avançada, maior extensão de acometimento na tomografia inicial, maior tempo de internação e maior proporção de internação em unidade de terapia intensiva (WEI et al., 2020).

Um estudo recente de acompanhamento de pacientes sobreviventes da SARS mostrou que, três anos após a infecção, 21,74% dos pacientes avaliados exibiam distúrbio ventilatório restritivo e 34,78% redução da capacidade de difusão ao monóxido de carbono (DLCO). Quinze anos após, nenhum apresentava distúrbio restritivo, mas 38,36% tinham redução da DLCO15. Dados semelhantes são observados em pacientes que foram acometidos pelo MERS-CoV; um ano após a infecção, 37% dos pacientes apresentavam redução na DLCO e 8% redução na capacidade vital forçada (CVF). A avaliação da função pulmonar em pacientes com COVID-19 no momento da alta revelou que 47,2% dos pacientes apresentavam redução na DLCO, 25% redução da capacidade pulmonar total (CPT), 13,6% redução do volume expirado no primeiro segundo (VEF1) e 9,1% na CVF. A redução da DCLO foi mais comum em pacientes que apresentaram pneumonia grave (MO et al., 2020).

Um dos principais sintomas respiratórios apresentados no pós-COVID-19 é a dispneia que pode ser relacionada a redução da força muscular respiratória. O desempenho dos músculos respiratórios pode ser afetado por vários fatores, como o envelhecimento, obesidade, sedentarismo, tabagismo e doenças crônicas. Em pacientes com doenças pulmonares crônicas, além da redução da força muscular respiratória, a demanda imposta ao músculo respiratório também aumenta em decorrência de alterações na resistência das vias aéreas e na mecânica da parede torácica. Uma análise de pacientes pós-COVI-19 na alta hospitalar, revelou que mais de um terço dos pacientes recuperados desenvolvem anormalidades fibróticas, o que aumenta a pressão necessária para respirar. O comprometimento pulmonar de longo prazo pode se desenvolver após a eliminação do vírus e, em particular, a doença pulmonar intersticial fibrótica, que pode estar relacionada, além de outras causas, com a inflamação crônica provocada pela COVID-19 (VASARMIDI et al., 2020). Portanto, pacientes que sofrem de infecção viral aguda podem apresentar desequilíbrios adicionais entre a capacidade de geração de força dos músculos respiratórios e as demandas impostas para respirar, aumentando o risco de insuficiência respiratória (SEVERIN et al., 2020).

As doenças respiratórias virais também estão associadas a consequências psicopatológicas agudas e de longa duração nos sobreviventes (BOHMWALD et al., 2018). Pacientes com COVID-19 podem apresentar delírio, ansiedade, depressão e

insônia, induzido por sequelas psicopatológicas por meio de infecção viral direta do sistema nervoso central (SNC) pelo coronavírus ou indiretamente por meio de uma resposta imune (WU et al., 2020).

Uma pesquisa que rastreou sintomas psiquiátricos em 402 adultos sobreviventes de COVID-19 mostrou que 28% dos pacientes se autoavaliaram para Transtorno do Estresse Pós-Traumático, 31% para depressão, 42% ansiedade, 20% para sintomas de Transtorno Obsessivo Compulsivo e 40% para insônia. Além disso, foi observado o Índice de Inflamação Imune Sistêmica Basal positivamente associado a escores de depressão e ansiedade no acompanhamento desses pacientes (MAZZA et al., 2020). Por isso, recomenda-se a avaliar a psicopatologia dos pacientes sobreviventes de COVID-19 para encaminhar quando necessário para diagnóstico e tratamento de condições psiquiátricas emergentes. O Consenso de Stanfort recomenda o acompanhamento desses pacientes para identificar efeitos psicológicos adversos, como resultado do COVID-19 e encaminhamento para serviços psicológicos (BARKER-DAVIES et al., 2020).

1.1.JUSTIFICATIVA

Existem evidências suficientes para uma possível Síndrome pós-COVID-19, designando sequelas com sintomas persistentes. A ATS recomenda que os pacientes pós-COVID-19 devem passar por uma avaliação de função física e emocional, função respiratória, capacidade de exercício em 6 a 8 semanas após a alta hospitalar e aqueles com necessidades de intervenções devem receber um programa de reabilitação abrangente de acordo com sua avaliação inicial (SPRUIT et al., 2020).

O treinamento muscular respiratório melhora a força muscular respiratória, a capacidade de exercício, a espessura do músculo diafragma e a dispneia em várias populações de paciente, especialmente naqueles com maior redução de força muscular respiratória basal. Os efeitos do treinamento muscular respiratório foram observados em protocolos com duração de apenas 4 semanas, facilitando a adesão ao treinamento (SEVERIN et al., 2020).

Diante dessas informações podemos constatar que pesquisas desse âmbito são necessárias para medir a extensão dos comprometimentos funcionais em pacientes pós-COVID-19. Além disso, as pesquisas devem avaliar se as intervenções de reabilitação, podem promover a melhora dos sintomas pós-infecção (SPRUIT et al., 2020).

Como um componente essencial do cuidado pós-agudo, a reabilitação visa reduzir a incapacidade de longo prazo e permite que os pacientes vivam na comunidade e retornem ao seu nível anterior de participação social. Ter um programa de reabilitação pós- COVID-19 bem projetado para responder à necessidade de cuidados e garantir a eficácia, eficiência e adequação do tratamento de reabilitação representa uma estratégia adequada (VOURGANAS; STANKOVIC; 2021).

As razões socioeconômicas e de saúde no mundo pós-COVID-19 requerem reabilitação domiciliar sem assistência direta de um profissional (VOURGANAS; STANKOVIC, 2021). A necessidade do distanciamento social, a vulnerabilidade de alguns grupos e a reestruturação das atividades presenciais exigiram uma readaptação rápida no âmbito da assistência para pacientes com necessidade de reabilitação, como o atendimento domiciliar não supervisionado e a tele reabilitação. Além disso, fácil acessibilidade ao tratamento em qualquer local e os baixos custos de um programa de reabilitação domiciliar são fatores que podem se tornar uma estratégia interessante e viável para acompanhar pacientes na fase pós-COVID-19.

Estudos anteriores realizados com pacientes em treinamento domiciliar produziram resultados clínicos de curto prazo que foram equivalentes a reabilitação pulmonar ambulatorial DPOC, mostrando também eficácia na redução do risco de exacerbação aguda da DPOC e hospitalizações (VASILOPOULOU et al., 2017), além de ser um modelo alternativo de baixo custo para os pacientes (BURGE et al., 2020).

1.2 OBJETIVOS

1.2.1 Objetivo primário

Avaliar a eficácia de um protocolo de treinamento muscular inspiratório domiciliar na melhora da força muscular respiratória, dispneia e qualidade de vida de pacientes pós-COVID-19.

1.2.2 Objetivos secundários

Avaliar após TMI a influência da:

- Função pulmonar;
- Percepção de esforço e dispneia;

- Tolerância ao exercício;
- Qualidade de vida;
- Força muscular periférica;
- Ansiedade e depressão;
- Estado funcional.

1.3 HIPÓTESE

Levanta-se a hipótese de que o protocolo de treinamento muscular inspiratório domiciliar pode ser efetivo e seguro para melhora da força muscular respiratória, dispneia, qualidade de vida, função pulmonar, percepção de esforço, tolerância ao exercício, força muscular periférica, ansiedade e depressão e estado funcional de pacientes pós-covid-19, gerando uma boa adesão dos participantes ao tratamento.

2 MATERIAIS E MÉTODOS

2.1 Tipo de Pesquisa

Trata-se de um estudo experimental do tipo ensaio clínico, randomizado, controlado e cego que será realizada no Instituto de Medicina Tropical da Universidade Federal do Rio Grande do Norte (UFRN).

2.2 População da pesquisa

Serão recrutados sujeitos de ambos os gêneros, na faixa etária acima de 18 anos, com diagnóstico confirmado de COVID-19, encaminhadas pelo ambulatório de Infectologia do Hospital Giselda Trigueiro da Secretaria Estadual do Município de Natal-Rio Grande do Norte (HGT/SESAP-RN).

2.3 Tamanho da amostra

O tamanho amostral será realizado através do software GPower versão 3.1.9.2 (Kiel, Alemanha) para Windows e será estabelecida após a realização de estudo piloto com 5 participantes em cada grupo (total de 10 sujeitos) para um teste ANOVA two-way hipotético, utilizando-se a variável principal a Pressão Inspiratória Máxima (PImáx.), em que a média e desvio padrão será utilizado para estimativa do tamanho do efeito, sendo

adotado um erro alfa de 0.05 com distribuição bilateral e um poder de teste de 80%. Será estabelecido um N amostral maior considerando uma perda de 20%.

2.4 Critérios de inclusão e exclusão

Serão considerados como critérios de inclusão sujeitos de ambos os sexos, sedentários, com diagnóstico de COVID-19 confirmado pelo RT-PCR, idade acima de 18 anos e sem qualquer doença respiratória de base, com estado cognitivo adequado definido através do Mini Exame do Estado Mental (MEEM) (ANEXO 1) e redução da força muscular respiratória, definida através da avaliação da Pimáx., de acordo com as seguintes equações de predição: homens (153,3 - 0,8 x idade) e mulheres (110,4 – 0,49 x idade) e considerando o limiar inferior de normalidade com erro padrão de estimativa (EPE) para homens de 17,3 e mulheres de 9,1 (NEDER et al., 1999).

Serão excluídos do estudo sujeitos que apresentem alguma condição que impossibilite a realização das avaliações e dos protocolos, intercorrências que justifiquem a interrupção da coleta de dados, como síncope, dor torácica intensa, tosse com sangue, aqueles que solicitarem saída do estudo e que apresentem efeitos adversos como o internamento por agudização do quadro clínico.

2.5 Procedimentos para seleção dos sujeitos

Para o recrutamento dos pacientes, os pesquisadores entrarão em contato com a equipe do HGT. Todos os pacientes contactados serão informados sobre a pesquisa e a sua importância. A participação na pesquisa será condicionada a assinatura do Termo de Consentimento Livre e Esclarecido (TCLE) (APÊNDICE A).

A amostra será randomizada automaticamente e alocada em dois grupos distintos através do site www.randomization.com, determinados como Grupo Experimental (GE): treino muscular inspiratório e Grupo Controle (G2): TMI placebo sem nenhuma carga. Os grupos serão codificados e a alocação será transferida para uma série de envelopes opacos selados e numerados sequencialmente.

Três avaliadores participarão da pesquisa: o pesquisador 1 ficará responsável apenas pelas avaliações, o pesquisador 2, pelo reajuste de carga e o pesquisador 3, pela randomização. O estudo será duplo-cego, pois o pesquisador 1 e os participantes não terão conhecimento da alocação dos sujeitos nos grupos, nem dos efeitos da intervenção.

2.6 Aspectos Éticos

Para sua execução, este projeto foi submetido à apreciação pelo Comitê de Ética em Pesquisa (CEP) da Universidade Federal do Rio Grande do Norte (UFRN) (CAAE: 45575421.7.0000.5537) e submetido ao Clinical Trials (NCT05077241). Será respeitada a autonomia e a garantia do anonimato das participantes, assegurando sua privacidade quanto a dados confidenciais, como rege a Resolução 510/16 do Conselho Nacional de Saúde e a declaração de Helsinki para pesquisa com seres humanos. Antes de admitidos no estudo, todos os voluntários assinarão o TCLE.

2.7 Desenho do Estudo

Os sujeitos incluídos na pesquisa passarão por três momentos de avaliações: Pré-treinamento (Inicial), Pós-Treinamento (6 semanas) e Teste de Retenção (24 semanas).

Após o recrutamento, os participantes serão convidados a comparecer ao Instituto de Medicina Tropical para realizar uma avaliação inicial por um avaliador previamente treinado e cego para o grupo de alocação da intervenção e incluirá anamnese e exame físico, com aferição dos sinais vitais, medidas antropométricas, avaliação dos volumes pulmonares, força muscular respiratória, força muscular periférica, qualidade de vida, ansiedade e depressão, status funcional e teste da caminhada de 6 minutos.

Após a avaliação inicial, todos os voluntários receberão um aparelho POWERbreathe® (POWERbreathe®, HaB Ltd, Southam, UK), para realização do treinamento, e serão orientados individualmente sobre como utilizá-lo e sobre a realização do protocolo. Eles realizarão uma sessão experimental para familiarização com o dispositivo que não será considerada para análise. A cada três dias os voluntários receberão uma ligação telefônica do pesquisador 2 que não participará da avaliação para confirmar se o exercício com o POWERbreathe® estava sendo realizado adequadamente na frequência e carga orientadas e se havia alguma dúvida em relação ao protocolo. Ao final de cada semana os participantes receberão uma ligação por vídeo do pesquisador 2 para realizar o ajuste no aparelho de acordo com a progressão de carga semanal do G1.

Todos os momentos de avaliações (pré-treinamento, pós-treinamento e teste de retenção) serão realizados por um único avaliador (Avaliador 1) – o qual não saberá em qual grupo o sujeito será alocado – e registrados na ficha de avaliação desenvolvida para o projeto. Um segundo pesquisador (Avaliador 2) será o responsável por aplicar os protocolos de treinamento nos sujeitos.

2.8 Procedimento de obtenção dos dados

2.8.1 Ficha de Avaliação Clínica

Após assinatura do TCLE, será realizada uma avaliação dos voluntários da pesquisa, por meio de uma ficha de avaliação (APÊNDICE B). Essa ficha terá a finalidade de direcionar a coleta de dados pessoais, sinais vitais, medidas antropométricas (peso, altura, índice de massa corpórea), antecedentes pessoais e patológicos, hábitos de vida, exame físico (inspeção e palpação) e exames complementares.

2.8.2 Medidas antropométricas

Para a avaliação do perfil antropométrico serão consideradas as seguintes variáveis: Massa Corporal (MC), estatura, Índice de Massa Corporal (IMC) e Perímetro da Panturrilha (PP).

Os participantes serão pesados em uma balança digital *FILIZOLA®* modelo 31 (Filizola®, São Paulo – SP, Brasil), com precisão de 100 gramas, aferida e certificada pelo Instituto Nacional de Metrologia, Normalização e Qualidade Industrial (INMETRO), com roupas leves, descalços, eretos, com os pés juntos, os braços estendidos ao longo do corpo, posicionados de costas para a medida da balança, permanecendo imóvel com o olhar fixo à frente até a realização da leitura (BRASIL, 2004).

Terão sua estatura mensurada por um estadiômetro integrado a balança, graduado em centímetro e precisão de 1 milímetro, durante a inspiração máxima e deverão estar em ortostatismo, com braços estendidos ao longo do corpo, coluna cervical alinhada, olhando para um ponto fixo na altura dos olhos, com os calcanhares, ombros e nádegas em contato com a parede, os maléolos internos se tocando, bem como a parte interna de ambos os joelhos (BRASIL, 2004).

O Índice de Massa Corporal (IMC) será calculado considerando-se a razão entre a massa corporal e o quadrado da estatura (kg/m²), sendo classificados por um gráfico de acordo com a idade em baixo peso (IMC $\leqq$ 22); eutrófico (IMC > 22 e < 27), sobrepeso (IMC $\geqq$ 27), segundo o Ministério da Saúde (2017).

2.8.3 Força muscular respiratória

A avaliação da força dos músculos respiratórios será realizada a partir da mensuração das pressões respiratórias máximas (Pressão Inspiratória Máxima: PI_{max} e Pressão Expiratória Máxima: PE_{max}) através da utilização do manovacuômetro analógico

(GERAR®, São Paulo, Brasil) com intervalo operacional de ± 300 cmH_2O e equipado com adaptador de bucais, contendo uma válvula de escape através de um orifício de aproximadamente 2mm de diâmetro para evitar o aumento da pressão intraoral.

Os participantes deverão utilizar um clipe nasal para evitar a saída de ar, permanecendo sentados em uma cadeira, com o dorso apoiado, flexão de quadril a 90 graus, membros superiores apoiados sobre as coxas e pés apoiados no chão, de modo a se sentirem confortáveis e relaxados.

Para a mensuração da PI_{max} o participante deverá realizar a expiração até alcançar seu Volume Residual (VR), conectando imediatamente a peça bucal na via oral e efetuando um esforço inspiratório máximo contra a via aérea ocluída, sustentando por no mínimo um segundo. Para a mensuração da PE_{max} o participante deverá inspirar até alcançar sua Capacidade Pulmonar Total (CPT) e em seguida, inserindo a peça bucal na cavidade oral, realizará um esforço expiratório máximo contra a via aérea ocluída, devendo ser mantido por no mínimo um segundo (ATS, 2002). Durante o esforço expiratório do paciente, o avaliador manterá uma pressão digital ao redor dos lábios, com o objetivo de prevenir o escape aéreo. Os participantes receberão incentivo constante por parte do avaliador durante a realização do teste.

Para análise dos dados serão utilizadas ao menos três manobras reprodutíveis, com variabilidade menor que 20%, e o valor mais alto será registrado. As referências de normalidade para o cálculo das pressões respiratórias máximas em função da idade e gênero respeitarão a descrição do método utilizado por Neder et al. (1999) para a população brasileira.

2.8.4 Volumes e Capacidades Pulmonares

Todos os participantes serão submetidos a uma avaliação por um espirômetro modelo *Koko Digidoser* (*Spide*, *Longmont*, USA) para verificar volumes e capacidades pulmonares. Todos os testes serão realizados por um mesmo avaliador por meio do espirômetro citado anteriormente, previamente calibrado e em ambiente climatizado.

Os indivíduos foram orientados a evitar refeições volumosas uma hora antes do exame, não consumir alimentos ou bebidas que contenham cafeína por pelo menos seis horas antes e não ingerir bebidas alcoólicas no dia do teste (ATS, 2019).

O teste consistia em uma manobra de inspiração até a capacidade pulmonar total (CPT), seguida de uma expiração máxima forçada até o volume residual (VR), realizadas

através do aparelho. As provas foram executadas na posição sentada com flexão de quadris e joelhos a 90°, de acordo com os critérios previamente estabelecidos pela *American Thoracic Society* (ATS, 2019). Serão realizados pelo menos três testes, com variação inferior a 5% e o maior valor obtido em um dos testes será comparado com os valores preditos dos parâmetros de função pulmonar para população brasileira. Serão avaliados o VEF_1 e a CVF. Os valores previstos serão calculados mediante os valores de referência (PEREIRA et al., 2007).

2.8.5 Avaliação da Dispneia

A dispneia será avaliada através da escala adaptada ao português do Medical Research Council (ANEXO 2). A escala permitirá ao voluntário indicar até que ponto sua falta de ar afetava sua mobilidade nas atividades diárias. Os escores variam de 1 a 5, cujos valores maiores referem maior dispneia, e será eleito pelo participante (BESTALL et al., 1999).

2.8.6 Avaliação da Percepção de esforço e fadiga

Antes e após o treinamento os indivíduos serão questionados separadamente quanto a sua percepção subjetiva de esforço durante a respiração e fadiga em membros inferiores. Para quantificar foi será a escala de Borg modificada (ANEXO 3). A escala apresentava valores numéricos de 0 a 10 e expressões que classificam grau de dificuldade, cujos valores maiores refletem maior sensação de falta de ar. Essa escala é comumente usada para medir desconforto, esforço, fadiga no repouso e durante o exercício, uma vez que a medição é feita de forma direta no momento em que o indivíduo está experimentando a sensação (BORG, 2007).

2.8.7 Avaliação da força de preensão manual

A força de preensão manual será medida com o dinamômetro hidráulico manual (Saehan®). Será quantificada medindo a quantidade de força isométrica máxima que a mão dominante pode apertar o dinamômetro. O participante será confortavelmente sentado em uma cadeira, joelhos fletidos a 90º ombro do braço dominante aduzido e neutralmente rodado, cotovelo fletido a 90º e junto ao tronco, antebraço em posição neutra

e punho entre 0º e 30º de extensão e 0º a 15º de desvio ulnar. Os participantes serão solicitados a apertar o dinamômetro com a máxima força isométrica, sem qualquer outro movimento do corpo, durante 5 segundos (NOVAES et al., 2009). Essa medida será realizada três vezes, com um minuto de intervalo entre elas, considerando-se a média para análise (PEREIRA et al., 2009). Serão considerados frágeis aqueles cuja média das três medidas estiver entre os 20% menores valores da distribuição, com ajustamento por gênero e índice de massa corporal (IMC - peso/altura^2), conforme as faixas sugeridas pela OMS (homens: $0 < IMC \leq 23$, ponto de corte (PC) $\leq$ 27,00kgf; $23 < IMC < 28$, PC $\leq$ 28,67kgf, $28 \leq IMC < 30$, PC $\leq$ 29,50; IMC $\geq$ 30, PC $\leq$ 28,67; mulheres: $0 < IMC \leq 23$, PC $\leq$ 16,33; $23 < IMC < 28$, PC $\leq$ 16,67; $28 \leq IMC < 30$, PC $\leq$ 17,33; IMC $\geq$ 30, PC $\leq$ 16,67) (MARUCCI; BARBOSA, 2003).

2.8.8 Teste da Caminhada de Seis Minutos (TC6')

O TC6' avalia nível submáximo de capacidade funcional durante o exercício, mensurando a distância que um paciente pode caminhar sobre uma superfície plana de 30 metros durante seis minutos. Para a realização, o participante será instruído a caminhar em velocidade máxima sustentada, mas sem correr, sendo possível parar para descansar e voltar ao teste, quando necessário. O avaliador irá monitorar a FC, SpO_2, percepção de dispneia (Borg-D) e fadiga (Borg-F) de membros inferiores (BORG, 1982) a cada volta e os participantes serão estimulados verbalmente a cada minuto de acordo com as frases padronizadas pelas recomendações do teste. Ao final dos seis minutos o número de voltas será registrado e um marcador colocado no chão ao lado do sujeito (ATS, 2002).

A distância percorrida na última volta será medida por uma fita métrica e a distância total obtida multiplicando-se o número de voltas pela medida do espaço e adicionando a distância percorrida da última volta (ATS, 2002). O método para avaliar a distância prevista seguirá as equações propostas na literatura, com base no gênero, peso, idade e altura dos participantes (ENRIGHT; SHERRILL, 1998).

A PA, FC, $SatO_2$, Borg-D e Borg-F serão avaliadas também no início e ao final do teste. Se os participantes apresentarem dor torácica, dispneia intolerável, queda da SpO2 inferior a 85%, câimbras musculares, sudorese, palidez e/ou vertigem, o teste será interrompido (ATS, 2002).

2.8.9 Avaliação da cognição

O Mini Exame do Estado Mental (MEEM) será utilizado para rastreamento cognitivo nos idosos. É composto por duas seções que avaliam funções cognitivas. A primeira seção contém itens que avaliam orientação, memória e atenção, totalizando 21 pontos; a segunda mede a capacidade de nomeação, de obediência a um comando verbal e a um escrito, de redação livre de uma sentença e de cópia de um desenho complexo (polígonos), totalizando 9 pontos. O escore final é de 30 pontos. Os valores mais altos do escore indicam maior desempenho cognitivo (FOLSTEIN et al., 1975). Serão utilizadas as notas de corte proposta para minimizar a influência do nível de escolaridade sobre os escores totais (BERTOLUCCI et al., 1994).

O participante deverá ser deixado à vontade, e não deverá se sentir julgado. Os eventuais erros cometidos por ele durante a aplicação não deverão ser corrigidos, para não os inibir. Será acrescido um ponto para cada resposta correta e zero para as respostas erradas ou não respondidas.

2.8.10 Medical Outcomes Study 36-Item Short Health Form Survey (SF-36)

O *Medical Outcomes Study 36-Item Short Health Form Survey (SF-36) (ANEXO 4)* é um questionário multidimensional traduzido e validado para a realidade brasileira (CICONELLI et al., 1999; LAGUARDIA et al., 2011) que foi desenvolvido para avaliar qualidade de vida relacionada à saúde. O instrumento é composto por 36 itens, distribuídos em 8 domínios que englobam dois grandes componentes: o físico (aspectos físicos, dor corporal, estado geral de saúde e função física) e o mental (aspectos emocionais, função social, aspectos mentais e vitalidade). As pontuações variam de 0 a 100 para cada subescala, sendo as pontuações mais altas indicativos de uma melhor qualidade de vida relacionada à saúde (WARE; SHERBOURNE, 1992).

2.8.11 International Physical Activity Questionnaire (IPAQ)

A versão longa do IPAQ (ANEXO 5), adaptada (MAZO; BENEDETTI, 2001) e validada para a população brasileira (BENEDETTI et al., 2008) é composta por 5 domínios e 15 questões e foi desenvolvida para avaliar o gasto energético semanal de atividades físicas relacionadas com o trabalho, transporte, tarefas domésticas e lazer. O

instrumento considera as atividades praticadas por pelo menos 10 minutos contínuos, com intensidade moderada e vigorosa, realizadas na semana anterior.

Será aplicado sob forma de entrevista por avaliadores previamente treinados e os idosos serão orientados a responder o questionário com base em uma semana habitual. Ao final, o entrevistador deverá somar os tempos (minutos e horas/dia) e anotar em cada questão o valor total em minutos e horas e dos dias da semana. Em seguida deverá somar os totais de cada domínio e assim calcular o total de toda a atividade física em minutos por semana. O gasto energético deverá ser calculado considerando os minutos por semana para cada atividade estimado em METs (BENEDETTI et al., 2008), utilizando o compêndio de Ainsworth et al. (2000). Os participantes serão classificados em muito ativo, ativo, irregularmente ativo e sedentário (MATSUDO et al., 2002).

2.8.12 Escala de depressão e ansiedade

As escalas de autoavaliação de depressão (SDS) e escala de autoavaliação de ansiedade (SAS) (ANEXO 6) serão exploradas para avaliar depressão e ansiedade nos grupos. Tanto o SDS quanto o SAS têm 20 itens, cada um dos quais será pontuado em uma escala de 1–4, e quanto maior a pontuação, mais grave o grau de depressão e ansiedade (ZUNG, 1991).

2.8.13 Escala de Estado Funcional Pos-Covid-19

A escala de estado funcional pós-covid-19 (ANEXO 7) avalia aspectos relevantes da vida diária durante o seguimento após a infecção. A escala destina-se a auxiliar os usuários a tornarem-se conscientes das limitações funcionais atuais em pacientes com Covid-19 e a determinar objetivamente os graus de limitações funcionais. A escala contém seis itens que variam de zero a cinco e compreende toda a gama de desfechos funcionais com foco nas limitações de tarefas e atividades de vida diária (domiciliares, laborais/de estudo e mudanças de estilo de vida). A classificação geral corresponde ao pior estado funcional indicado pelas respostas dos pacientes (o grau mais alto correspondente às maiores limitações) (KLOK et al.,2020).

2.8.14 Avaliação dos efeitos adversos e adesão

Para a avaliação dos efeitos adversos será fornecido um diário de treinamento (APÊNDICE C) para todos os voluntários do estudo, no qual estarão dispostos todos os dias de treinamento que eles devem fazer e um espaço em branco para observações positivas e/ou negativas e para registrarem qualquer intercorrência durante e/ou após as sessões de treinamento. Para análise da adesão ao treinamento serão consideradas todas as vezes que eles marcarem nas tabelas sinalizando a realização da sessão. Será realizada a soma de todas as sessões feitas e estas serão divididas pelo número total de sessões que os participantes devem fazer.

A análise dos efeitos adversos será realizada considerando todas as intercorrências registradas no diário ou comentadas durante as avaliações finais.

2.9 PROTOCOLO DE INTERVENÇÃO DA PESQUISA

Ao serem randomizados os sujeitos serão alocados em dois grupos (GE e GC). Serão adotados os seguintes protocolos de treinamento para os respectivos grupos:

Protocolo GE: TMI com 30% da Pimáx. com incremento de carga semanal de 10% do valor da Pimáx. inicial. As sessões serão compostas de 30 repetições, 2 vezes ao dia, uma pela manhã e outra à tarde, 7 dias consecutivos na semana, durante 6 semanas. Os indivíduos serão orientados a realizar uma contração rápida dos músculos inspiratórios e sustentá-la durante 2 segundos, em cada manobra, e terão a possibilidade de descansar a cada 3 repetições do TMI, durante 30 segundos, para evitar fadiga muscular ou qualquer outra intercorrência.

Protocolo GC: Os indivíduos irão utilizar um dispositivo de TMI sem qualquer carga e receberão as mesmas orientações do G1. Ao final da pesquisa o grupo controle terá direito ao tratamento experimental com o protocolo de TMI, caso este apresente eficácia.

A pesquisa será custeada pelos pesquisadores e que os aparelhos serão fornecidos de forma gratuita para os participantes do estudo.

2.10 RISCOS

Esta pesquisa apresenta riscos mínimos aos participantes. Estes podem se sentir constrangidos durante a entrevista, tendo em vista a natureza do conteúdo de alguns

questionários, que ele poderá recusar-se a responder, ou pela dificuldade em realizar alguma avaliação específica. Poderão apresentar alguns desconfortos como cefaleia, vertigens, náuseas, oscilação da pressão arterial e até um pequeno desequilíbrio devido a exigência de determinados testes físicos e treinamento, no entanto os avaliadores usarão critérios de contraindicações ou de interrupções dos testes quando necessário, para minimizar os riscos à saúde dos participantes. O participante poderá recusar-se a realizar qualquer procedimento a qualquer momento, sem que haja prejuízo para ele.

Além disso, também podem apresentar preocupações quanto ao sigilo dos dados coletados e a assinatura que deverão realizar no TCLE. O pesquisador irá auxiliá-los esclarecendo todas as dúvidas existentes e informar aos participantes que os dados coletados serão utilizados apenas para fins acadêmicos, e que não haverá divulgação deles.

2.11 BENEFÍCIOS

Os resultados obtidos com o desenvolvimento desta pesquisa podem garantir a possibilidade de ampliar as intervenções relacionadas à reabilitação pulmonar em pacientes pós-covid-19, através de um treinamento efetivo, acessível, seguro e de baixo custo. As informações levantadas também poderão contribuir para o rastreamento de pacientes após o período de infecção aguda, contribuindo para o entendimento a cerca dessa nova temática, o desenvolvimento e avaliação da efetividade de intervenções direcionadas a melhorar a saúde dessa população.

2.12 ANÁLISE DOS DADOS

Para análise dos dados será utilizado o *software* SPSS (*Statistical Package for the Social Sciences)* versão 22.0 para *Windows*. O teste de normalidade será realizado de acordo com a quantidade de participantes voluntários da pesquisa. Portanto, poderá ser utilizado o teste de normalidade de Shapiro-Wilk ou o teste de normalidade de Kolmorov-Smirnov para as variáveis desejadas.

As variáveis que apresentarem distribuição não paramétricas serão comparadas através dos testes Wilcoxon (análise intragrupos) e Mann-Whitney (análise intergrupos) e quando a distribuição ocorrer de forma paramétrica será utilizado o

teste ANOVA two-way. Quando houver diferença significativa, o teste *post hoc* de Dunn's será aplicado a fim de se localizar as diferenças.

Na análise descritiva, será realizada uma caracterização da população estudada, através da obtenção das médias e desvios-padrão para variáveis de distribuição normal ou mediana e intervalo interquartil (25%-75%) para variáveis de distribuição assimétrica. Para minimizar um eventual erro do tipo I será estabelecido o nível de significância de 5%. O poder do estudo e o tamanho do efeito serão expostos nos principais resultados do estudo.

3 DESFECHOS

3.1 DESFECHOS PRIMÁRIOS

Serão considerados como desfechos primários a força muscular respiratória, dispneia e qualidade de vida.

3.2 DESFECHOS SECUNDÁRIOS

Serão considerados como desfechos secundários: função pulmonar, tolerância ao exercício, estado funcional, ansiedade e depressão, força muscular periférica, efeitos adversos e adesão.

4 CRONOGRAMA DE EXECUÇÃO DAS ATIVIDADES

Atividades 2021	Jan	Fev	Mar	Abr	Mai	Jun	Jul	Ago	Set	Out	Nov	Dez
Revisão bibliográfica												
Elaboração do projeto												
Submissão do projeto à Plataforma Brasil												
Coleta de dados												

Atividades 2022	Jan	Fev	Mar	Abr	Mai	Jun	Jul	Ago	Set	Out	Nov	Dez
Coleta de dados												

Atividades 2023	Jan	Fev	Mar	Abr	Mai	Jun	Jul	Ago	Set	Out	Nov	Dez
**Coleta de dados**												
**Análise dos dados**												
**Redação dos manuscritos**												
**Defesa**												

5 ORÇAMENTO

**Material de consumo (Custeio)**	**Valor (R$) Total**
Serviço de gráfica	500,00
Serviços de tradução	400,00
Transporte	500,00
Canetas	30,00
Espirômetro (1)	27.000,00
Manovacuômetro (1)	1.700,00
Dinamômetro (1)	5.000,00
Power Breath (10)	5.000,00
Balança (1)	50,00
**Total de Despesas**	**40.180,00**

REFERÊNCIAS

AINSWORTH, Barbara E. et al. Compendium of physical activities: an update of activity codes and MET intensities. **Medicine and science in sports and exercise**, v. 32, n. 9; SUPP/1, p. S498-S504, 2000.

BARKER-DAVIES, Robert M. et al. The Stanford Hall consensus statement for post-COVID-19 rehabilitation. **British journal of sports medicine**, v. 54, n. 16, p. 949-959, 2020.

BARKER-DAVIES, Robert M. et al. The Stanford Hall consensus statement for post-COVID-19 rehabilitation. **British journal of sports medicine**, v. 54, n. 16, p. 949-959, 2020.

BENEDETTI, Tania B.; MAZO, Giovana Z.; DE BARROS, Mauro VG. Aplicação do questionário internacional de atividades físicas para avaliação do nível de atividades física de mulheres idosas: Validade concorrente e reprodutibilidade teste-reteste. **Revista Brasileira de ciência e movimento**, v. 12, n. 1, p. 25-34, 2008.

BERTOLUCCI, Paulo HF et al. O mini-exame do estado mental em uma população geral: impacto da escolaridade. **Arquivos de Neuro-psiquiatria**, v. 52, n. 1, p. 01-07, 1994.

BESTALL, J. C. et al. Usefulness of the Medical Research Council (MRC) dyspnoea scale as a measure of disability in patients with chronic obstructive pulmonary disease. **Thorax**, v. 54, n. 7, p. 581-586, 1999.

BOHMWALD, Karen et al. Neurologic alterations due to respiratory virus infections. **Frontiers in cellular neuroscience**, v. 12, p. 386, 2018.

BORG E. On perceived exertion and its measurement. **Psychology**. 2007. 1927-34.

BURGE, Angela T. et al. Home-based pulmonary rehabilitation for COPD using minimal resources: An economic analysis. **Respirology**, v. 25, n. 2, p. 183-190, 2020.

BURNHAM, Ellen L. et al. Chest computed tomography features are associated with poorer quality of life in acute lung injury survivors. **Critical care medicine**, v. 41, n. 2, p. 445, 2013.

CARFÌ, Angelo et al. Persistent symptoms in patients after acute COVID-19. **Jama**, v. 324, n. 6, p. 603-605, 2020.

CICONELLI, Rozana Mesquita et al. Tradução para a língua portuguesa e validação do questionário genérico de avaliação de qualidade de vida SF-36 (Brasil SF-36). **Rev bras reumatol**, v. 39, n. 3, p. 143-50, 1999.

ENRIGHT, Paul L.; SHERRILL, Duane L. Reference equations for the six-minute walk in healthy adults. **American journal of respiratory and critical care medicine**, v. 158, n. 5, p. 1384-1387, 1998.

FOLSTEIN, Marshal F.; FOLSTEIN, Susan E.; MCHUGH, Paul R. “Mini-mental state”: a practical method for grading the cognitive state of patients for the clinician. **Journal of psychiatric research**, v. 12, n. 3, p. 189-198, 1975.

GRAHAM, Brian L. et al. Standardization of spirometry 2019 update. An official American thoracic society and European respiratory society technical statement. **American journal of respiratory and critical care medicine**, v. 200, n. 8, p. e70-e88, 2019.

HOLLAND, Anne E. et al. An official European Respiratory Society/American Thoracic Society technical standard: field walking tests in chronic respiratory disease. **European Respiratory Journal**, v. 44, n. 6, p. 1428-1446, 2014.

HUANG, Yhu-Chering; LEE, Ping-Ing; HSUEH, Po-Ren. Evolving reporting criteria of COVID-19 in Taiwan during the epidemic. **Journal of Microbiology, Immunology and Infection**, v. 53, n. 3, p. 413-418, 2020.

KLOK, Frederikus A. et al. The Post-COVID-19 Functional Status scale: a tool to measure functional status over time after COVID-19. **European Respiratory Journal**, v. 56, n. 1, 2020.

LAGUARDIA, Josué et al. Psychometric evaluation of the SF-36 (v. 2) questionnaire in a probability sample of Brazilian households: results of the survey Pesquisa Dimensões Sociais das Desigualdades (PDSD), Brazil, 2008. **Health and Quality of Life Outcomes**, v. 9, n. 1, p. 61, 2011.

LAI, Chih-Cheng et al. Extra-respiratory manifestations of COVID-19. International journal of antimicrobial agents, v. 56, n. 2, p. 106024, 2020.

MARUCCI, Maria de Fátima Nunes et al. Estado nutricional e capacidade física. **O Projeto SABE no Município de São Paulo: uma abordagem inicial. Brasília: OPAS/MS**, p. 95-117, 2003.

MATSUDO, Sandra Mahecha et al. Nível de atividade física da população do Estado de São Paulo: análise de acordo com o gênero, idade, nível socioeconômico, distribuição geográfica e de conhecimento. **Revista brasileira de ciência e movimento**, v. 10, n. 4, 2008.

MAZO, Giovana Zarpellon et al. Validade concorrente e reprodutibilidade: teste-reteste do Questionário de Baecke modificado para idosos. **Revista Brasileira de Atividade Física & Saúde**, v. 6, n. 1, p. 5-11, 2001.

MAZZA, Mario Gennaro et al. Anxiety and depression in COVID-19 survivors: Role of inflammatory and clinical predictors. **Brain, behavior, and immunity**, v. 89, p. 594-600, 2020.

MINISTÉRIO DA SAÚDE (Brasil). Sistema de Vigilância Alimentar e Nutricional. Operações básicas para a coleta, processamento, análise de dados e informação em serviços de saúde. Brasília, DF: Ministério da Saúde; 2004.

MINISTÉRIO DA SAÚDE. **Avaliação do peso IMC na terceira idade.** 30 de maio, 2017.

MO, Xiaoneng et al. Abnormal pulmonary function in COVID-19 patients at time of hospital discharge. **European Respiratory Journal**, v. 55, n. 6, 2020.

NEDER, Jose Alberto et al. Reference values for lung function tests: II. Maximal respiratory pressures and voluntary ventilation. **Brazilian journal of medical and biological research**, v. 32, n. 6, p. 719-727, 1999.

NOVAES, Rômulo Dias et al. Equações de referência para a predição da força de preensão manual em brasileiros de meia idade e idosos. **Fisioterapia e Pesquisa**, v. 16, n. 3, p. 217-222, 2009.

ORGANIZAÇÃO MUNDIAL DA SAÚDE. Painel do Coronavírus da OMS (COVID-19). 2022. Retirado de: https://covid19.who.int/.

PARK, Wan Beom et al. Correlation between pneumonia severity and pulmonary complications in Middle East respiratory syndrome. **Journal of Korean medical science**, v. 33, n. 24, 2018.

PEREIRA, Carlos Alberto de Castro; SATO, Taeko; RODRIGUES, Sílvia Carla. New reference values for forced spirometry in white adults in Brazil. **Jornal Brasileiro de Pneumologia**, v. 33, p. 397-406, 2007.

ROONEY, Scott; WEBSTER, Amy; PAUL, Lorna. Systematic Review of Changes and Recovery in Physical Function and Fitness After Severe Acute Respiratory Syndrome–Related Coronavirus Infection: Implications for COVID-19 Rehabilitation. **Physical Therapy**, v. 100, n. 10, p. 1717-1729, 2020.

SEEßLE, Jessica et al. Persistent Symptoms in adult patients 1 year after coronavirus disease 2019 (COVID-19): a prospective cohort study. **Clinical infectious diseases: an official publication of the Infectious Diseases Society of America**, 2021.

SEVERIN, Richard et al. Respiratory muscle performance screening for infectious disease management following COVID-19: a highly pressurized situation. **The American journal of medicine**, 2020.

SPRUIT, Martijn A. et al. COVID-19: interim guidance on rehabilitation in the hospital and post-hospital phase from a European Respiratory Society-and American Thoracic Society-coordinated international task force. **European respiratory journal**, v. 56, n. 6, 2020.

VASARMIDI, Eirini et al. Pulmonary fibrosis in the aftermath of the COVID-19 era. **Experimental and therapeutic medicine**, v. 20, n. 3, p. 2557-2560, 2020.

VASILOPOULOU, Maroula et al. Home-based maintenance tele-rehabilitation reduces the risk for acute exacerbations of COPD, hospitalisations and emergency department visits. **European Respiratory Journal**, v. 49, n. 5, 2017.

VOURGANAS, Ioannis; STANKOVIC, Vladimir; STANKOVIC, Lina. Individualised responsible Artificial Intelligence for home-based rehabilitation. **Sensors**, v. 21, n. 1, p. 2, 2021.

WARE JR, John E.; SHERBOURNE, Cathy Donald. The MOS 36-item short-form health survey (SF-36): I. Conceptual framework and item selection. **Medical care**, p. 473-483, 1992.

WEI, Jiangping et al. Analysis of thin-section CT in patients with coronavirus disease (COVID-19) after hospital discharge. **Journal of X-ray Science and Technology**, n. Preprint, p. 1-7, 2020.

WU, Yeshun et al. Nervous system involvement after infection with COVID-19 and other coronaviruses. **Brain, behavior, and immunity**, v. 87, p. 18-22, 2020.

WU, Zunyou; MCGOOGAN, Jennifer M. Characteristics of and important lessons from the coronavirus disease 2019 (COVID-19) outbreak in China: summary of a report of 72 314 cases from the Chinese Center for Disease Control and Prevention. **Jama**, v. 323, n. 13, p. 1239-1242, 2020.

ZHANG, Peixun et al. Long-term bone and lung consequences associated with hospital-acquired severe acute respiratory syndrome: a 15-year follow-up from a prospective cohort study. **Bone research**, v. 8, n. 1, p. 1-8, 2020.

ZHOU, Fei et al. Clinical course and risk factors for mortality of adult inpatients with COVID-19 in Wuhan, China: a retrospective cohort study. **The lancet**, v. 395, n. 10229, p. 1054-1062, 2020.

ZUNG, William W. A rating instrument for anxiety disorders. **Psychosomatics: Journal of Consultation and Liaison Psychiatry**, 1971.

ANEXOS

ANEXO 1 - Mini Exame do Estado Mental (MEEM)

MINI EXAME DO ESTADO MENTAL

1) Como o Sr(a) avalia sua memória atualmente?

(1) muito boa (2) boa (3) regular (4) ruim (5) péssima

(6) não sabe

Total de pontos: ☐

2) Comparando com um ano atrás, o Sr (a) diria que sua memória está:

(1) melhor (2) igual (3) pior (4) não sabe

Total de pontos: ☐

ORIENTAÇÃO TEMPORAL:

Anote um ponto para cada resposta certa:

3) Por favor, diga-me:

Dia da semana ( ) Dia do mês ( ) Mês ( ) Ano ( ) Hora aprox. ( )

Total de pontos: ☐

ORIENTAÇÃO ESPACIAL:

Anote um ponto para cada resposta certa

4) Responda:

Onde estamos: consultório, hospital, residência ( )

Em que lugar estamos: andar, sala, cozinha ( )

Em que bairro estamos: ( )

Em que cidade estamos ( )

Em que estado estamos ( )

Total de pontos: ☐

REGISTRO DA MEMÓRIA IMEDIATA:

5) Vou lhe dizer o nome de três objetos e quando terminar, pedirei para repeti-los, em qualquer ordem. Guarde-os que mais tarde voltarei a perguntar: Arvore, Mesa, Cachorro.

A ( ) M ( ) C ( )

Obs: Leia os nomes dos objetos devagar e de forma clara, somente um a vez e anote. Se o total for diferente de três: - repita todos os objetos até no máximo três repetições; - anote o número de repetições que fez _____; - nunca corrija a primeira parte; anote um ponto para cada objeto lembrado e zero para os que não foram lembrados.

Total de pontos: ☐

ATENÇÃO E CALCULO:

6) Vou lhe dizer alguns números e gostaria que realizasse os seguintes cálculos:

100-7; 93-7; 86-7; 79-7; 72-7;

____; ____; ____; ____; ____.

(93; 86; 79; 72; 65) **Total de pontos:** ☐

MEMÓRIA RECENTE:

7) Há alguns minutos, o Sr (a) repetiu uma série de três palavras. Por favor, diga-me agora quais ainda se lembra: A ( ) M ( ) C ( )

Obs: anote um ponto para cada resposta correta: Arvore, Mesa, Cachorro.

Total de pontos: ☐

LINGUAGEM:

Anote um ponto para cada resposta correta:

8) Aponte a caneta e o relógio e peça pra nomeá-los: C ( ) R ( )

(permita dez segundos para cada objeto)

Total de pontos: ☐

9) Repita a frase que eu vou lhe dizer (pronunciar em voz alta, bem articulada e lentamente)

"NEM AQUI, NEM ALÍ, NEM LÁ"

Total de pontos: ☐

10) Dê ao entrevistado uma folha de papel, na qual esteja escrito em letras grandes: "FECHE OS OLHOS". Diga-lhe : leia este papel e faça o que está escrito (permita dez segundos).

Total de pontos: ☐

11) Vou lhe dar um papel e quando eu o entregar, pegue com sua mão direita, dobre-o na metade com as duas mãos e coloque no chão.

P ( ) D ( ) C ( )

Total de pontos: ☐

12) Pedir ao entrevistado que escreva uma frase em um papel em branco.

O Sr (a) poderia escrever uma frase completa de sua escolha? (contar um ponto se a frase tem sujeito, verbo, predicado, sem levar em conta erros de ortografia ou de sintaxe). **Se o entrevistado não fizer corretamente, perguntar-lhe: "Isto é uma frase/ E permitir-lhe corrigir se tiver consciência de seu erro.** (máximo de trinta segundos).

Total de pontos: ☐

13) Por favor, copie este desenho. (entregue ao entrevistado o desenho e peça-o para copiar). **A ação está correta se o desenho tiver dois pentágonos com intersecção de um ângulo.** Anote um ponto se o desenho estiver correto.

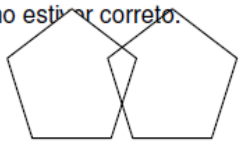

Total de pontos: ☐

Obs: Somente as respostas corretas anotadas nas perguntas de 03 a 13 e anote o total. A pontuação máxima é de trinta pontos.

TOTAL ☐

ANEXO 2 - Escala de Dispneia do MRC

**VERSÃO EM PORTUGUÊS DA ESCALA DE DISPNÉIA DO *MEDICAL RESEARCH COUNCIL* (MRC)**	
**Grau 1**	Só sofre falta de ar durante exercícios intensos.
**Grau 2**	Sofre de falta de ar quando anda apressadamente ou subindo uma rampa leve.
**Grau 3**	Anda mais devagar do que as pessoas da mesma idade por causa de falta de ar ou tem que parar para respirar mesmo quando andando devagar.
**Grau 4**	Para para respirar depois de andar menos de 100 metros ou após alguns minutos.
**Grau 5**	Sente tanta falta de ar que não sai mais de casa, ou quando está se vestindo.

ANEXO C – Escala modificada de Borg

**ESCALA MODIFICADA DE BORG**	
**0**	Nenhuma
**0,5**	Muito, muito leve
**1**	Muito leve
**2**	Leve
**3**	Moderada
**4**	Pouco intensa
**5**	Intensa
**6**	
**7**	Muito intensa
**8**	
**9**	Muito, muito intensa
**10**	Máxima

ANEXO 3 - Medical Outcomes Study 36-Item Short Health Form Survey (SF-36)

Versão Brasileira do Questionário de Qualidade de Vida -SF-36

1- Em geral você diria que sua saúde é:

Excelente	Muito Boa	Boa	Ruim	Muito Ruim
1	2	3	4	5

2- Comparada há um ano atrás, como você se classificaria sua idade em geral, agora?

Muito Melhor	Um Pouco Melhor	Quase a Mesma	Um Pouco Pior	Muito Pior
1	2	3	4	5

3- Os seguintes itens são sobre atividades que você poderia fazer atualmente durante um dia comum. Devido à sua saúde, você teria dificuldade para fazer estas atividades? Neste caso, quando?

Atividades	Sim, dificulta muito	Sim, dificulta um pouco	Não, não dificulta de modo algum
a) Atividades Rigorosas, que exigem muito esforço, tais como correr, levantar objetos pesados, participar em esportes árduos.	1	2	3
b) Atividades moderadas, tais como mover uma mesa, passar aspirador de pó, jogar bola, varrer a casa.	1	2	3
c) Levantar ou carregar mantimentos	1	2	3
d) Subir vários lances de escada	1	2	3
e) Subir um lance de escada	1	2	3
f) Curvar-se, ajoelhar-se ou dobrar-se	1	2	3
g) Andar mais de 1 quilômetro	1	2	3
h) Andar vários quarteirões	1	2	3
i) Andar um quarteirão	1	2	3
j) Tomar banho ou vestir-se	1	2	3

4- Durante as últimas 4 semanas, você teve algum dos seguintes problemas com seu trabalho ou com alguma atividade regular, como conseqüência de sua saúde física?

	Sim	Não
a) Você diminui a quantidade de tempo que se dedicava ao seu trabalho ou a outras atividades?	1	2
b) Realizou menos tarefas do que você gostaria?	1	2
c) Esteve limitado no seu tipo de trabalho ou a outras atividades.	1	2
d) Teve dificuldade de fazer seu trabalho ou outras atividades (p. ex. necessitou de um esforço extra).	1	2

5- Durante as últimas 4 semanas, você teve algum dos seguintes problemas com seu trabalho ou outra atividade regular diária, como conseqüência de algum problema emocional (como se sentir deprimido ou ansioso)?

	Sim	Não
a) Você diminui a quantidade de tempo que se dedicava ao seu trabalho ou a outras atividades?	1	2
b) Realizou menos tarefas do que você gostaria?	1	2
c) Não realizou ou fez qualquer das atividades com tanto cuidado como geralmente faz.	1	2

6- Durante as últimas 4 semanas, de que maneira sua saúde física ou problemas emocionais interferiram nas suas atividades sociais normais, em relação à família, amigos ou em grupo?

De forma nenhuma	Ligeiramente	Moderadamente	Bastante	Extremamente
1	2	3	4	5

7- Quanta dor no corpo você teve durante as últimas 4 semanas?

Nenhuma	Muito leve	Leve	Moderada	Grave	Muito grave
1	2	3	4	5	6

8- Durante as últimas 4 semanas, quanto a dor interferiu com seu trabalho normal (incluindo o trabalho dentro de casa)?

De maneira alguma	Um pouco	Moderadamente	Bastante	Extremamente
1	2	3	4	5

9- Estas questões são sobre como você se sente e como tudo tem acontecido com você durante as últimas 4 semanas. Para cada questão, por favor dê uma resposta que mais se aproxime de maneira como você se sente, em relação às últimas 4 semanas.

	Todo Tempo	A maior parte do tempo	Uma boa parte do tempo	Alguma parte do tempo	Uma pequena parte do tempo	Nunca
a) Quanto tempo você tem se sentindo cheio de vigor, de vontade, de força?	1	2	3	4	5	6
b) Quanto tempo você tem se sentido uma pessoa muito nervosa?	1	2	3	4	5	6
c) Quanto tempo você tem se sentido tão deprimido que nada pode anima-lo?	1	2	3	4	5	6
d) Quanto tempo você tem se sentido calmo ou tranqüilo?	1	2	3	4	5	6
e) Quanto tempo você tem se sentido com muita energia?	1	2	3	4	5	6
f) Quanto tempo você tem se sentido desanimado ou abatido?	1	2	3	4	5	6
g) Quanto tempo você tem se sentido esgotado?	1	2	3	4	5	6
h) Quanto tempo você tem se sentido uma pessoa feliz?	1	2	3	4	5	6
i) Quanto tempo você tem se sentido cansado?	1	2	3	4	5	6

10- Durante as últimas 4 semanas, quanto de seu tempo a sua saúde física ou problemas emocionais interferiram com as suas atividades sociais (como visitar amigos, parentes, etc)?

Todo Tempo	A maior parte do tempo	Alguma parte do tempo	Uma pequena parte do tempo	Nenhuma parte do tempo
1	2	3	4	5

11- O quanto verdadeiro ou falso é cada uma das afirmações para você?

	Definitivamente verdadeiro	A maioria das vezes verdadeiro	Não sei	A maioria das vezes falso	Definitiva-mente falso
a) Eu costumo obedecer um pouco mais facilmente que as outras pessoas	1	2	3	4	5
b) Eu sou tão saudável quanto qualquer pessoa que eu conheço	1	2	3	4	5
c) Eu acho que a minha saúde vai piorar	1	2	3	4	5
d) Minha saúde é excelente	1	2	3	4	5

CÁLCULO DOS ESCORES DO QUESTIONÁRIO DE QUALIDADE DE VIDA

Fase 1: Ponderação dos dados

Questão	Pontuação	
01	Se a resposta for<br>1<br>2<br>3<br>4<br>5	Pontuação<br>5,0<br>4,4<br>3,4<br>2,0<br>1,0
02	Manter o mesmo valor	
03	Soma de todos os valores	
04	Soma de todos os valores	
05	Soma de todos os valores	
06	Se a resposta for<br>1<br>2<br>3<br>4<br>5	Pontuação<br>5<br>4<br>3<br>2<br>1

<table>
<tr><td>07</td><td>Se a resposta for
1
2
3
4
5
6</td><td>Pontuação
6,0
5,4
4,2
3,1
2,0
1,0</td></tr>
<tr><td>08</td><td colspan="2">A resposta da questão 8 depende da nota da questão 7
Se 7 = 1 e s [illegible] alor da questão é (6)
Se 7 = 2 à 6 [illegible] valor da questão é (5)
Se 7 = 2 à 6 e se 8 = 2, o valor da questão é (4)
Se 7 = 2 à 6 e se 8 = 3, o valor da questão é (3)
Se 7 = 2 à 6 e se 8 = 4, o valor da questão é (2)
Se 7 = 2 à 6 e se 8 = 3, o valor da questão é (1)

Se a questão 7 não for respondida, o escorre da questão 8 passa a ser o seguinte:
Se a resposta for (1), a pontuação será (6)
Se a resposta for (2), a pontuação será (4,75)
Se a resposta for (3), a pontuação será (3,5)
Se a resposta for (4), a pontuação será (2,25)
Se a resposta for (5), a pontuação será (1,0)</td></tr>
<tr><td>09</td><td colspan="2">Nesta questão, a pontuação para os itens a, d, e ,h, deverá seguir a seguinte orientação:
Se a resposta for 1, o valor será (6)
Se a resposta for 2, o valor será (5)
Se a resposta for 3, o valor será (4)
Se a resposta for 4, o valor será (3)
Se a resposta for 5, o valor será (2)
Se a resposta for 6, o valor será (1)
Para os demais itens (b, c,f,g, i), o valor será mantido o mesmo</td></tr>
<tr><td>10</td><td colspan="2">Considerar o mesmo valor.</td></tr>
<tr><td>11</td><td colspan="2">Nesta questão os itens deverão ser somados, porém os itens b e d deverão seguir a seguinte pontuação:
Se a resposta for 1, o valor será (5)
Se a resposta for 2, o valor será (4)
Se a resposta for 3, o valor será (3)
Se a resposta for 4, o valor será (2)
Se a resposta for 5, o valor será (1)</td></tr>
</table>

Fase 2: Cálculo do Raw Scale

Nesta fase você irá transformar o valor das questões anteriores em notas de 8 domínios que variam de 0 (zero) a 100 (cem), onde 0 = pior e 100 = melhor para cada domínio. É chamado de raw scale porque o valor final não apresenta nenhuma unidade de medida.
Domínio:

- Capacidade funcional
- Limitação por aspectos físicos
- Dor
- Estado geral de saúde
- Vitalidade
- Aspectos sociais
- Aspectos emocionais

- Saúde mental

Para isso você deverá aplicar a seguinte fórmula para o cálculo de cada domínio:

Domínio:
Valor obtido nas questões correspondentes – Limite inferior x 100
Variação (Score Range)

Na fórmula, os valores de limite inferior e variação (Score Range) são fixos e estão estipulados na tabela abaixo.

Domínio	Pontuação das questões correspondidas	Limite inferior	Variação
Capacidade funcional	03	10	20
Limitação por aspectos físicos	04	4	4
Dor	07 + 08	2	10
Estado geral de saúde	01 + 11	5	20
Vitalidade	09 (somente os itens a + e + g + i)	4	20
Aspectos sociais	06 + 10	2	8
Limitação por aspectos emocionais	05	3	3
Saúde mental	09 (somente os itens b + c + d + f + h)	5	25

Exemplos de cálculos:

- Capacidade funcional: (ver tabela)

Domínio: Valor obtido nas questões correspondentes – limite inferior x 100
Variação (Score Range)

Capacidade funcional: $\frac{21 - 10}{20} \times 100 = 55$

O valor para o domínio capacidade funcional é 55, em uma escala que varia de 0 a 100, onde o zero é o pior estado e cem é o melhor.

- Dor (ver tabela)

- Verificar a pontuação obtida nas 7 e 08; por exemplo: 5,4 e 4, portanto somando-se as duas, teremos: 9,4

- Aplicar fórmula:
Domínio: Valor obtido nas questões correspondentes – limite inferior x 100
Variação (Score Range)

Dor: $\frac{9{,}4 - 2}{10} \times 100 = 74$

O valor obtido para o domínio dor é 74, numa escala que varia de 0 a 100, onde zero é o pior estado e cem é o melhor.

Assim, você deverá fazer o cálculo para os outros domínios, obtendo oito notas no final, que serão mantidas separadamente, não se podendo soma-las e fazer uma média.

Obs.: A questão número 02 não faz parte do cálculo de nenhum domínio, sendo utilizada somente para se avaliar o quanto o indivíduo está melhor ou pior comparado a um ano atrás.

Se algum item não for respondido, você poderá considerar a questão se esta tiver sido respondida em 50% dos seus itens.

ANEXO 4 - *International Physical Activity Questionnaire (IPAQ)*

QUESTIONÁRIO INTERNACIONAL DE ATIVIDADE FÍSICA

Versão 8 (forma longa, semana usual)

Nome:____ Data: ___/___ /___ Idade: ____ anos

Orientações do Entrevistador

Nesta entrevista estou interessado em saber que tipo de atividades físicas o(a) senhor(a) faz em uma semana normal (típica). Suas respostas ajudarão a entender quanto ativos são as pessoas de sua idade.

As perguntas que irei fazer estão relacionadas ao tempo que você gasta fazendo atividades físicas no trabalho, em casa (no lar), nos deslocamentos à pé ou de bicicleta e no seu tempo de lazer (esportes, exercícios, etc.).

Portanto, considere como **atividades físicas** todo movimento corporal que envolve algum esforço físico. Lembre que as atividades VIGOROSAS são aquelas que precisam de um grande esforço físico e que fazem o(a) senhor(a) respirar MUITO mais forte que o normal. As atividades físicas MODERADAS são aquelas que exigem algum esforço físico e que fazem o(a) senhor(a) respirar um pouco mais forte que o normal.

SEÇÃO 1 - ATIVIDADE FÍSICA NO TRABALHO

Esta seção inclui as atividades que você faz no seu trabalho, seja ele remunerado ou voluntário. Inclua as atividades que você faz na universidade, faculdade ou escola. Você não deve incluir as tarefas domésticas, cuidar do jardim e da casa ou tomar conta da sua família. Estas serão incluídas na seção 3.

1 a. Atualmente você tem ocupação remunerada ou faz trabalho voluntário fora de sua casa?

○ SIM ○ NÃO → *Vá para seção 2 - Transporte*

Orientações do Entrevistador

- As próximas questões são em relação ao tempo que você passa no trabalho (fora de casa) seja ele remunerado ou voluntário.
- Por favor, NÃO INCLUA o transporte para o trabalho.
- Pense apenas naquelas atividades que durem pelo menos 10 minutos contínuos.

1b. Em quantos dias de uma semana normal você participa (realiza) atividades físicas vigorosas, de forma contínua por pelo menos 10 minutos (exemplo: trabalho de construção pesada, levantar e transportar objetos pesados, cortar lenha, serrar madeira, cortar grama, pintar casa, cavar valas ou buracos, etc.)?

☐ DIAS por semana ☐ Não faz AF vigorosas → *Vá para questão* ***1c***

Tempo em cada dia?

DIA	Segunda	Terça	Quarta	Quinta	Sexta	Sábado	Domingo
Tempo							

1c. Em quantos dias de uma semana normal você participa (realiza) atividades físicas MODERADAS, de forma contínua por pelo menos 10 minutos (exemplo: levantar e transportar pequenos objetos, limpar vidros, varrer ou limpar o chão, carregar crianças no colo, lavar roupas com as mãos, etc.)?

☐ DIAS por semana ☐ Não faz AF moderadas → *Vá para questão* ***1d***

Tempo em cada dia?

DIA	Segunda	Terça	Quarta	Quinta	Sexta	Sábado	Domingo
Tempo							

1d. Em quantos dias de uma semana normal você realiza caminhadas no seu trabalho, de forma contínua por pelo menos 10 minutos?

Orientações do Entrevistador	▸ Lembre que você não deve incluir a caminhada que você realiza para ir para o trabalho ou para voltar para casa, após o trabalho.

☐ DIAS por semana ☐ Não faz caminhadas → *Vá para seção 2 - Transporte*

Tempo em cada dia?

DIA	Segunda	Terça	Quarta	Quinta	Sexta	Sábado	Domingo
Tempo							

SEÇÃO 2 - ATIVIDADE FÍSICA COMO MEIO DE TRANSPORTE

As perguntas desta seção estão relacionadas às atividades que você realiza para se deslocar de um lugar para outro. Você deve incluir os deslocamentos para o trabalho (se você trabalha), encontro do grupo de terceira idade, cinema, supermercado, lojas ou qualquer outro local.

2a. Em quantos dias de uma semana normal você anda de carro, ônibus, metrô ou trem?

☐ DIAS por semana ☐ Não utiliza veículos a motor → *Vá para a questão 2b*

Tempo em cada dia?

DIA	Segunda	Terça	Quarta	Quinta	Sexta	Sábado	Domingo
Tempo							

Orientações do Entrevistador	▸ Agora pense somente em relação aos deslocamentos que você realiza à pé ou de bicicleta para ir de um lugar para outro! Não inclua as atividades que você faz por diversão ou exercício.

2b. Em quantos dias de uma semana normal você anda de bicicleta, por pelo menos 10 minutos contínuos, para ir de um lugar para outro, ?

☐ DIAS por semana ☐ Não anda de bicicleta → *Vá para a questão 2c*

Tempo em cada dia?

DIA	Segunda	Terça	Quarta	Quinta	Sexta	Sábado	Domingo
Tempo							

2c. Em quantos dias de uma semana normal você caminha por pelo menos 10 minutos contínuos, para ir de um lugar para outro?

☐ DIAS por semana ☐ Não faz caminhadas → *Vá para a Seção 3*

Tempo em cada dia?

DIA	Segunda	Terça	Quarta	Quinta	Sexta	Sábado	Domingo
Tempo							

SEÇÃO 3 - ATIVIDADE FÍSICA EM CASA, TAREFAS DOMÉSTICAS E ATENÇÃO À FAMÍLIA

As perguntas desta seção estão relacionadas às atividades que o(a) senhor(a) realiza na sua casa e ao redor da sua casa. Nestas atividades estão incluídas as tarefas no jardim ou quintal, manutenção da casa e aquelas que você faz para tomar conta da sua família.

3a. Em quantos dias de uma semana normal você faz atividades físicas vigorosas no jardim ou quintal, por pelo menos 10 minutos contínuos? (Exemplo: carpir, cortar lenha, serrar, pintar, levantar e transportar objetos pesados, cortar grama com tesoura, etc.).

☐ DIAS por semana ☐ Não faz AF vigorosas em casa → *Vá para questão 3b*

Tempo em cada dia?

DIA	Segunda	Terça	Quarta	Quinta	Sexta	Sábado	Domingo
Tempo							

3b. Em quantos dias de uma semana normal você faz atividades físicas moderadas no jardim ou quintal, por pelo menos 10 minutos contínuos? (Exemplo: levantar e carregar pequenos objetos, limpar a garagem, jardinagem, caminhar ou brincar com crianças, etc.).

☐ DIAS por semana ☐ Não faz AF moderadas no quintal → *Vá para questão 3c*

Tempo em cada dia?

DIA	Segunda	Terça	Quarta	Quinta	Sexta	Sábado	Domingo
Tempo							

3c. Em quantos dias de uma semana normal você faz atividades físicas moderadas dentro da sua casa, por pelo menos 10 minutos contínuos? (Exemplo: , limpar vidros ou janelas, lavar roupas à mão, limpar banheiro, esfregar o chão, carregar crianças pequenas no colo, etc).

☐ DIAS por semana ☐ Não faz AF moderadas em casa → *Vá para a seção 4*

Tempo em cada dia?

DIA	Segunda	Terça	Quarta	Quinta	Sexta	Sábado	Domingo
Tempo							

SEÇÃO 4 - ATIVIDADE FÍSICA DE RECREAÇÃO, ESPORTE, EXERCÍCIO E LAZER

As perguntas desta seção estão relacionadas às atividades que o(a) senhor(a) realiza em uma semana normal (habitual) unicamente por recreação, esporte, exercício ou lazer. Pense somente nas atividades físicas que você faz por pelo menos 10 minutos contínuos. Por favor NÃO inclua atividades que você já tenha citado.nas seções

4a. No seu tempo livre, sem incluir qualquer caminhada que você já tenha citado nas perguntas anteriores, em quantos dias de uma semana normal você caminha, por pelo menos 10 minutos contínuos?

☐ DIAS por semana ☐ Não faz caminhadas no lazer → *Vá para questão 4b*

Tempo em cada dia?

DIA	Segunda	Terça	Quarta	Quinta	Sexta	Sábado	Domingo
Tempo							

4b. No seu tempo livre, durante uma semana normal em quantos dias você participa de atividades físicas vigorosas, por pelo menos 10 minutos contínuos? (Exemplo: correr, nadar rápido, pedalar rápido, canoagem, remo, musculação, esportes em geral, etc).

☐ DIAS por semana ☐ Não faz AF vigorosas no lazer → *Vá para questão 4c*

Tempo em cada dia?

DIA	Segunda	Terça	Quarta	Quinta	Sexta	Sábado	Domingo
Tempo							

4c. No seu tempo livre, durante uma semana normal em quantos dias você participa de atividades físicas moderadas por pelo menos 10 minutos contínuos? (Exemplo: pedalar em ritmo moderado, voleibol recreativo, natação, hidroginástica, ginástica e dança, etc).

☐ DIAS por semana ☐ Não faz AF moderadas no lazer → *Vá para Seção 5*

Tempo em cada dia?

DIA	Segunda	Terça	Quarta	Quinta	Sexta	Sábado	Domingo
Tempo							

SEÇÃO 5 - TEMPO QUE VOCÊ PASSA SENTADO

Esta é a última pergunta. Preciso saber quanto tempo em média o(a) senhor(a) passa sentado em cada dia da semana. Inclua todo o tempo que você passa sentado em casa, no trabalho, lendo, assistindo TV, visitando amigos, sentado no ônibus, etc.

Tempo em cada dia?

DIA	Segunda	Terça	Quarta	Quinta	Sexta	Sábado	Domingo
Tempo							

ANEXO 5 – Escala de Depressão e Ansiedade

	Quase nunca	Algumas vezes	Boa parte do tempo	A maior parte do tempo
1. Sinto-me abatido e triste.	1	2	3	4
2. De manhã é quando me sinto melhor.	4	3	2	1
3. Tenho crises de choro ou tenho vontade de chorar.	1	2	3	4
4. Tenho dificuldades em dormir à noite.	1	2	3	4
5. Como tanto quanto comia antes.	4	3	2	1
6. Continuo gostando de sexo.	4	3	2	1
7. Percebi que estou perdendo peso.	1	2	3	4
8. Tenho problemas de prisão de ventre.	1	2	3	4
9. Meu coração bate mais depressa do que antes.	1	2	3	4
10. Fico cansado sem nenhum motivo.	1	2	3	4
11. Minha mente está lúcida como antes.	4	3	2	1
12. Acho a mesma facilidade de antes para fazer as coisas.	4	3	2	1
13. Sinto-me inquieto e não consigo ficar parado.	1	2	3	4
14. Tenho esperanças em relação ao futuro.	4	3	2	1
15. Sinto-me mais irritado do que de costume.	1	2	3	4
16. Acho fácil tomar decisões.	4	3	2	1
17. Sinto-me útil e necessário.	4	3	2	1
18. Minha vida é bastante compensadora.	4	3	2	1
19. Acho que seria bom para os outros se eu morresse.	1	2	3	4
20. Continuo gostando das coisas em geral como antes.	4	3	2	1

Anote suas notas na margem direita e some-as. As notas devem ser interpretadas desta forma:

20-22: Você é super saudável (ou está se enganando!).

23-29: Você está sentindo algum estresse.

30-39: Você está enfraquecido por um nível baixo de depressão (ou esgotamento) e precisa de alguma ajuda ou, no caso de esgotamento, de algumas mudanças sérias em sua vida.

40-59: Você está seriamente debilitado pela depressão (ou esgotamento) e precisa de aconselhamento e ajuda espiritual.

60-80: Você está praticamente paralisado pela depressão (ou esgotamento) e precisa de aconselhamento e ajuda espiritual urgente e profunda, provavelmente precisando de terapia profissional e/ou tratamento sério em relação à restauração.

ANEXO 6 –Escala de Estado Funcional Pós-Covid.

**ESCALA DE ESTADO FUNCIONAL PÓS-COVID-19**	
**1.Sobrevivência**	
1.1 O paciente morreu após o diagnóstico de COVID-19?	□ Sim □ Não
**2. Cuidados Constantes**	
2.1 Você precisa de cuidados constantes?	□ Sim □ Não
**3. Atividades Básicas da Vida Diária (AVD)**	
3.1 É fundamental ter assistência para comer?	□ Sim □ Não
3.2 É fundamental ter assistência para usar o banheiro?	□ Sim □ Não
3.3 É fundamental ter assistência para rotina diária de higiene?	□ Sim □ Não
3.4 É fundamental ter assistência para caminhar?	□ Sim □ Não
**4. Atividades Instrumentais da Vida Diária (AIVD)**	
4.1 É fundamental a assistência para realizar tarefas domésticas básicas, importantes para a vida diária?	□ Sim □ Não
4.2 É fundamental assistência para realizar viagens locais?	□ Sim □ Não
4.3 É fundamental assistência para realizar compras locais?	□ Sim □ Não
**5. Participação em papéis sociais usuais**	
5.1 É fundamental a adaptação para realizar tarefas/atividades em casa ou no trabalho/estudo por você ser incapaz de realiza-las sozinho? (ex. resultando em mudança no nível de responsabilidade, mudança no trabalho/estudo de período integral para parcial)	□ Sim □ Não
5.2 Você ocasionalmente precisa evitar ou reduzir tarefas/atividades em casa ou no trabalho/estudo ou precisa distribuí-las ao longo do tempo? (mesmo você sendo capaz de realizar todas essas atividades)?	□ Sim □ Não
5.3 Você não consegue mais cuidar bem dos entes queridos como antes? (crianças, parceiros, pais, netos ou outros dependentes)	□ Sim □ Não
5.4 Desde o diagnóstico da COVID-19, houve problemas nos relacionamentos ou você ficou isolado? (problemas de comunicação, dificuldades no relacionamento com as pessoas em casa ou no trabalho/estudo, perda de amizades no isolamento)	□ Sim □ Não
5.5 Você está restrito a participar de atividades sociais e de lazer? (incluindo passatempos e interesses como ir a um restaurante, bar, cinema, passear, jogar, ler livros)	□ Sim □ Não
**6. CHECKLIST DE SINTOMAS**	
6.1 Você apresenta sintomas durante as tarefas/atividades diárias que precisam ser evitadas, reduzidas ou distribuídas ao longo do tempo?	□ Sim □ Não
6.2 Você apresenta algum sintoma resultante da COVID-19 que não causam limitações funcionais?	□ Sim □ Não
6.3 Você tem dificuldades em relaxar ou percebe a COVID-19 como um trauma?	□ Sim □ Não

APÊNDICES

APÊNDICE A – Termo de Consentimento Livre e Esclarecido (TCLE)

Termo de Consentimento Livre e Esclarecido - TCLE

Esclarecimentos

Este é um convite para você participar da pesquisa: "Eficácia do treinamento muscular inspiratório domiciliar em pacientes pós-covid-19: ensaio clínico randomizado", que tem como pesquisadora responsável a Profª Patrícia Angélica de Miranda Silva Nogueira.

Esta pesquisa pretende avaliar a eficácia de um protocolo de treinamento muscular inspiratório domiciliar na melhora da força muscular respiratória, dispneia e qualidade de vida de pacientes pós-Covid-19.

Caso você decida participar, deverá responder perguntas sobre informações pessoais sem identificá-lo, uso de medicamentos e hábitos de vida. Você irá medir peso, altura; verificar a pressão arterial, as batidas do seu coração e a quantidade de oxigênio no seu corpo e medir também a circunferência da batata de sua perna. Também será realizado um teste para você apertar em uma mola e soprar em um aparelho para verificar a força dos seus músculos e a função do seu pulmão. Você também vai realizar um teste para caminhar durante seis minutos e responder questionários sobre seu nível de atividade física, sua qualidade de vida, sobre ansiedade e depressão e estado funcional Pós-Covid-19. Responderá também um teste para verificar a capacidade da sua memória e investigação sobre falta de ar e cansaço nas pernas. Estes testes não são difíceis e não vão fazer mal à saúde. A realização de todas as medidas será em 1 dia que serão previamente agendados, de forma que você não seja prejudicado(a). Todos os exercícios serão previamente explicados e qualquer instrução quanto à vestimenta ou calçado será dado com antecedência.

Para realização dos exercícios domiciliares, você receberá um aparelho POWERbreathe®, e deverá realizar os exercícios, duas vezes por dia (manhã e tarde), sete dias por semana, durante 6 semanas consecutivas. Em cada sessão você deverá realizar 30 repetições do exercício e você poderá descansar a cada 3 repetições, durante 30 segundos à 1 minuto. Na primeira semana os exercícios serão realizados de duas formas durante 6 semanas: com 30% da máxima força dos músculos inspiratórios ou sem carga, com aumento da carga a cada semana. A forma de realizar o exercício será escolhida através de sorteio para cada voluntário. Os voluntários serão reavaliados após 6 e 24 semanas de treinamento.

As atividades realizadas serão de caráter não invasivo, ou seja, NÃO serão realizados procedimentos que envolvam corte, penetração de instrumentos, coleta de sangue ou que possam gerar dor ou desconforto. No entanto, você poderá cansar durante as avaliações e treinamento, o que será minimizado com intervalos para descanso entre os procedimentos. Esta pesquisa apresenta riscos mínimos aos participantes. Estes podem se sentir constrangidos durante a entrevista, tendo em vista a natureza do conteúdo de alguns questionários, que ele poderá recusar-se a responder, ou pela dificuldade em realizar alguma avaliação específica. Poderão apresentar alguns desconfortos como cefaleia, vertigens, náuseas, oscilação da pressão arterial e até um pequeno desequilíbrio devido a exigência de determinados testes físicos e treinamento, no entanto os avaliadores usarão critérios de contraindicações ou de interrupções dos testes quando necessário, para minimizar os riscos à saúde dos participantes.

Os benefícios ao participar da pesquisa são: conhecimento do resultado de uma avaliação fisioterapêutica para

______________ **(rubrica do Participante/Responsável legal)** __________________ **(rubrica do Pesquisador)**

o seu estado de saúde, maior entendimento sobre os efeitos fisiológicos do treinamento para músculos respiratórios, a fim de que seja realizada uma prescrição mais adequada desse treinamento, assim como a verificação da utilidade desse programa de exercícios para pacientes no período pós-covid-19.

Todas as informações obtidas serão sigilosas e o seu nome não será identificado em nenhum momento. Os dados serão guardados em local seguro e a divulgação dos resultados será feita de forma a não identificar os voluntários.

A pesquisa será custeada pelos pesquisadores e que os aparelhos serão fornecidos de forma gratuita para os participantes do estudo.

Em caso de algum problema que o(a) senhor(a) possa ter relacionado com a pesquisa, vocês terão direito a assistência gratuita que será prestada pelo pesquisador responsável.

Durante todo o período da pesquisa você poderá tirar suas dúvidas ligando para a doutoranda Gabriely Azevêdo Gonçalo Silva, (84)99939-0004.

Esses dados serão guardados pelo pesquisador responsável por essa pesquisa em local seguro e por um período de 5 anos.

Se você tiver algum gasto pela sua participação nessa pesquisa, eles serão assumidos pelo pesquisador e reembolsado para você.

Se você sofrer qualquer dano decorrente desta pesquisa, sendo ele imediato ou tardio, previsto ou não, você será indenizado.

Qualquer dúvida sobre a ética dessa pesquisa você deverá ligar para o Comitê de Ética em Pesquisa – instituição que avalia a ética das pesquisas antes que elas comecem e fornece proteção aos participantes das mesmas – da Universidade Federal do Rio Grande do Norte, nos telefones (84) 3215-3135 / (84) 9.9193.6266, através do e-mail cepufrn@reitoria.ufrn.br ou pelo formulário de contato do site <www.cep.propesq.ufrn.br> . Você ainda pode ir pessoalmente à sede do CEP, de segunda a sexta, das 08:00h às 12:00h e das 14:00h às 18:00h, na Universidade Federal do Rio Grande do Norte, Campus Central. Lagoa Nova. Natal/RN.CEP 59078-970.

Este documento foi impresso em duas vias. Uma ficará com você e a outra com o pesquisador responsável Profª Patrícia Angélica de Miranda Silva Nogueira.

Consentimento Livre e Esclarecido

Após ter sido esclarecido sobre os objetivos, importância e o modo como os dados serão coletados nessa pesquisa, além de conhecer os riscos, desconfortos e benefícios que ela trará para mim e ter ficado ciente de todos os meus direitos, concordo em participar da pesquisa "Eficácia do treinamento muscular inspiratória domiciliar em pacientes pós-covid-19: ensaio clínico randomizado", e autorizo a divulgação das informações por mim fornecidas em congressos e/ou publicações científicas desde que nenhum dado possa me identificar.

Natal, ____/_____/________.

Assinatura do participante da pesquisa

____

Impressão datiloscópica do participante

Declaração do pesquisador responsável “Eficácia do treinamento muscular inspiratória domiciliar em pacientes pós-covid-19: ensaio clínico randomizado” declaro que assumo a inteira responsabilidade de cumprir fielmente os

_____________ (rubrica do Participante/Responsável legal) _________________ (rubrica do Pesquisador)

procedimentos metodologicamente e direitos que foram esclarecidos e assegurados ao participante desse estudo, assim como manter sigilo e confidencialidade sobre a identidade dele.

Declaro ainda estar ciente que na inobservância do compromisso ora assumido infringirei as normas e diretrizes propostas pela Resolução 466/12 do Conselho Nacional de Saúde – CNS, que regulamenta as pesquisas envolvendo o ser humano.

Natal, ___/___/____.

Assinatura do pesquisador responsável

____

APÊNDICE B - Controle do treinamento

CONTROLE DO TMI

Nome:____

Avaliação inicial: ___/____/____

**1ª semana**	Dia 2	Dia 3	Dia 4	Dia 5	Dia 6	**Descanso**
Dia 1 ___/___/__	___/___/__	___/___/__	___/___/__	___/___/__	___/___/__	
1ª sessão						
2ª sessão						

**2ª semana**	Dia 2	Dia 3	Dia 4	Dia 5	Dia 6	**Descanso**
Dia 1 ___/___/__	___/___/__	___/___/__	___/___/__	___/___/__	___/___/__	
1ª sessão						
2ª sessão						

**3ª semana**	Dia 2	Dia 3	Dia 4	Dia 5	Dia 6	**Descanso**
Dia 1 ___/___/__	___/___/__	___/___/__	___/___/__	___/___/__	___/___/__	
1ª sessão						
2ª sessão						

**4ª semana**	Dia 2	Dia 3	Dia 4	Dia 5	Dia 6	**Descanso**
Dia 1 ___/___/__	___/___/__	___/___/__	___/___/__	___/___/__	___/___/__	
1ª sessão						
2ª sessão						

**5ª semana**	Dia 2	Dia 3	Dia 4	Dia 5	Dia 6	**Descanso**
Dia 1 ___/___/__	___/___/__	___/___/__	___/___/__	___/___/__	___/___/__	
1ª sessão						
2ª sessão						

**6ª semana** Dia 1 ___/___/__	Dia 2 ___/___/__	Dia 3 ___/___/__	Dia 4 ___/___/__	Dia 5 ___/___/__	Dia 6 ___/___/__	**Descanso**
1ª sessão						
2ª sessão						

APÊNDICE C – Ficha de avaliação clínica

FICHA DE AVALIAÇÃO CLÍNICA
PROJETO PÓS-COVID-19

**DADOS PESSOAIS**		
Nome:	RG:	Gênero: □ M □ F
Naturalidade:	Data de nascimento:	CPF:
Endereço:		
Telefones para contato:	E-mail:	
Profissão:	□ Aposentado	
Escolaridade (anos):		
Raça / cor da pele: □ branca □ preta □ amarela	□ parda	□ indígena □ não declarar
Renda familiar: □ menos que 1 salário mínimo □ entre 1 e 3 salários mínimos □entre 4 e 9	salários mínimos	□ 10 ou mais salários mín
Mora com: □Família □Sozinho □ Outro		
**ANTECEDENTES PESSOAIS**		
□ HAS □ DM □ Obesidade □ Sedentarismo □ IAM □ IC	□ AVE	□ DPOC □Asma
□ IRC □ Outros:		
**HÁBITOS DE VIDA**		
□ Consumo de bebida alcóolica: □ sim □ não □ Menos de uma vez por mês □ Uma vez ou mais por mês<br>□ Consumo de tabaco: □ sim □ não		
□ Consumo de tabaco: □ sim □ não □ diariamente □ menos que diariamente<br>SE fuma, há quanto tempo? Quantos maços por dia?<br>E no passado, o(a) sr(a) fumou? □ sim, diariamente □ sim, menos que diariamente □ não, nunca fumei		
**COVID-19**		
□ Data do diagnóstico: □ Data da internação hospitalar:<br>□Sintomas na internação hospitalar:		
□ Número total de dias de hospitalização: □ Número de dias de UTI:<br>Data da alta hospitalar:		
O(A) sr(a) foi □ sim □ não □ Se sim, qual foi o número de dias intubado(a)?<br>Oxigenoterapia: □ sim □ não □ Tempo:<br>Fez/faz uso de corticóide: □ sim □ não □Tempo:<br>Faz uso de oxigênio atualmente: □ sim □ não<br>□ Sintomas atuais:		
**EXAME CLÍNICO**		
Peso: _______ Altura: ________ IMC: _______ PA: ______________ FC: _________ FR: ______ CP:_________ CA: _____		
Saúde autorreferida (SAR)	Em geral, como o(a) sr(a) avalia a sua saúde?	□ muito boa □ boa □ regular □ ruim □ muito ruim
Tosse*	O(A) sr(a) está tossindo?	□ não estou tossindo<br>□ sim, tosse seca, irritativa<br>□ sim, tosse com catarro (Qual é a coloração? _________________________________)
Expansibilidade torácica**	O(A) sr(a) consegue respirar profundamente?	□ não<br>□ sim, mas com dificuldade<br>□ sim, sem nenhuma dificuldade
Dispneia (Borg modificada)	Como o(a) sr(a) avalia a sua sensação de falta de ar agora? Em uma escala de zero a dez, onde (leia as alternativas)	□ 0 nenhuma sensação de falta de ar<br>□ 0,5 muito, muito leve<br>□ 1 muito leve<br>□ 2 leve<br>□ 3 moderada<br>□ 4 pouco intensa<br>□ 5 intensa<br>□ 6<br>□ 7 muito intensa<br>□ 8<br>□ 9 muito, muito intensa

		☐ 10 máxima
Dispneia (MRC - Medical Research Council)	Como o(a) sr(a) avalia a sua sensação de falta de ar? (escolha uma alternativa)	☐ 1) só sofre de falta de ar durante exercícios intensos.<br>☐ 2) sofre de falta de ar quando andando apressadamente ou subindo uma rampa leve.<br>☐ 3) anda mais devagar do que pessoas da mesma idade por causa de falta de ar ou tem que parar para respirar mesmo quando andando devagar.<br>☐ 4) para para respirar depois de andar menos de 100 metros ou após alguns minutos.<br>☐ 5) sente tanta falta de ar que não sai mais de casa, ou quando está se vestindo.

FORÇA DE PREENSÃO PALMAR

	Data	1ª	2ª	3ª	4ª	5ª
Avaliação 1						
Avaliação 2						
Avaliação 3						
Avaliação 4						

FUNÇÃO PULMONAR

**Avaliação**	**Espirometria**	1° teste	2° teste	3° teste	% previsto	Valor previsto
Avaliação 1<br>Data:	CVF					
	CI					
	VEF1					
	PFE					
	VEF1/CVF%					
Avaliação 2<br>Data:	CVF					
	CI					
	VEF1					
	PFE					
	VEF1/CVF%					
Avaliação 3<br>Data:	CVF					
	CI					
	VEF1					
	PFE					
	VEF1/CVF%					
Avaliação 4<br>Data:	CVF					
	CI					
	VEF1					
	PFE					
	VEF1/CVF%					

FORÇA MUSCULAR RESPIRATÓRIA

		1º valor	2º valor	3º valor	% previsto	Valor previsto
Avaliação 1<br>Data:	PImáx.					
	PEmáx.					
Avaliação 2<br>Data:	PImáx.					
	PEmáx.					
Avaliação 3<br>Data:	PImáx.					
	PEmáx.					
Avaliação 4<br>Data:	PImáx.					
	PEmáx.					

Teste da Caminhada de 6 minutos (TC6')

**Avaliação**		Repouso	6 minutos
Avaliação 1<br>Data:	PA		
	FC		
	SPO2		
	FR		
	BORG-D		
	BORG-F		
Avaliação 2<br>Data:	PA		
	FC		
	SPO2		
	FR		
	BORG-D		
	BORG-F		
Avaliação 3<br>Data:	PA		
	FC		
	SPO2		
	FR		
	BORG-D		
	BORG-F		
Avaliação 4<br>Data:	PA		
	FC		
	SPO2		
	FR		
	BORG-D		
	BORG-F		

**TREINAMENTO MUSCULAR INSPIRATÓRIO**						
	**Semana 1 (30% PImáx)**	**Semana 2 (40% PImáx.)**	**Semana 3 (50% Pimáx.)**	**Semana 4 (60% Pimáx.)**	**Semana 5 (60% Pimáx)**	**Semana 6 (60% Pimáx.)**
**Carga de treino**						
