# Supplementary material for: Efficacy of home-based inspiratory muscle training in patients post-covid-19: Protocol for a randomized clinical trial
Source: PLoS One. 2023 May 4;18(5):e0279310. doi: 10.1371/journal.pone.0279310 (PMC10159136; doi:10.1371/journal.pone.0279310)
Supplement: S4 File — (DOCX) [file pone.0279310.s005.docx]

**FEDERAL UNIVERSITY OF RIO GRANDE DO NORTE**

**CENTER FOR HEALTH SCIENCES**

**GRADUATE PROGRAM IN PHYSIOTHERAPY**

**EFFICACY OF HOME INSPIRATORY MUSCLE TRAINING IN POST-COVID-19 PATIENTS: RANDOMIZED CLINICAL TRIAL.**

**GABRIELY AZEVÊDO GONÇALO SILVA**

**NATAL/RN**

**2021**

**GABRIELY AZEVÊDO GONÇALO SILVA**

**EFFICACY OF HOME INSPIRATORY MUSCLE TRAINING IN POST-COVID-19 PATIENTS: RANDOMIZED CLINICAL TRIAL.**

Research project presented to the Graduate Program in Physiotherapy of the Federal University of Rio Grande do Norte for consideration by the Ethics Committee in research with human beings.

Area of concentration: Evaluation and intervention in Physiotherapy in the cardiovascular and respiratory systems.

Advisor: Profa. Dr. Patrícia Angélica de Miranda Silva Nogueira.

**NATAL/RN**

**2021**

**SUMMARY**

**INTRODUCTION:** Coronavirus disease 2019 (COVID-19), caused by Coronavirus Severe Acute Respiratory Syndrome (SARS) 2 (SARS-CoV-2) exceeded the global number of 119,603,761 cases, with more than 2,649,722 deaths reported. There is sufficient evidence for a possible post-covid-19 syndrome, designating sequelae with persistent symptoms. Respiratory muscle training improves respiratory muscle strength, exercise capacity, diaphragm muscle thickness, and dyspnea in several patient populations, especially those with greater reduction in basal respiratory muscle strength.  **OBJECTIVE:** To evaluate the efficacy of a home inspiratory muscle training protocol in improving respiratory muscle strength, dyspnea and quality of life of post-Covid-19 patients.  **MATERIAS AND METHODS:** This is a clinical trial, controlled, randomized and blind, which will be conducted at the Institute of Tropical Medicine of the Federal University of Rio Grande do Norte. The sample size will be performed using the GPower software version 3.1.9.2 (Kiel, Germany) for Windows and will be established after a pilot study with 5 participants in each group (total of 10 subjects) for a hypothetical two-way ANOVA test, using the main variable the Maximum Inspiratory Pressure (Max). The subjects included in the research will go through three evaluation moments: Pre-training (Initial), Post-Training (6 weeks) and Retention Test (24 weeks) for clinical evaluation form, anthropometric measurements, respiratory muscle strength, lung volumes and capacities, dyspnea, perceived effort and fatigue, hand grip strength, six-minute walk test, anxiety and depression, post-covid functional status. After the initial evaluation, all volunteers will receive a POWERbreathe device® (POWERbreathe®, HaB Ltd, Southam, UK) for training.

**Keywords:** spirometry, covid-19, respiratory muscle strength.

**SUMMARY**

**1INTRODUCTION5**

1.1JUSTIFICATION8

1.2OBJECTIVES9

**1.2.1Primary** objective9

**1.2.2Secondary endpoints** 9

1.3HIPÓTESE10

**2MATERIALS AND** METHODS10

2.1TYPE OF RESEARCH10

2.2SEARCH POPULATION10

2.3SAMPLE SIZE10

2.4 INCLUSION AND EXCLUSION CRITERIA10

2.5 PROCEDURES FOR THE SELECTION OF SUBJECTS11

2.6 ETHICAL ASPECTS11

2.7 STUDY DESIGN11

2.8 DATA COLLECTION  **PROCEDURE12**

**2.8.1 Clinical evaluation**  **form12**

**2.8.2 Anthropometric measurements13**

**2.8.3 Respiratory muscle strength13**

**2.8.4 Lung volumes and capacities13**

**2.8.5 Dyspnea**  **assessment15**

**2.8.6 Perceived effort and**  **fatigue15**

**2.8.7 Handgrip**  **strength15**

**2.8.9 Six-minute walk**  **test16**

**2.8.10 Medical Outcomes Study 36-Item Short Health Form Research (SF-36) 17**

**2.8.11 International Physical Activity Questionnaire 17**

**2.8.12 Anxiety and Depression Scale18**

**2.8.13 Post-COVID-1918 functional status**  **scale**

**2.8.14 Evaluation of adverse effects and adhering18**

2.9 RESEARCH INTERVENTION PROTOCOL....................... ................... 19th

2.10 RISKS*.......................*  *............................................................................*  *...............*  *.* 19th

2.11 BENEFITS*.....................*  *...................................................*  *............*  *..*  *..*  *.* 20th

2.12 DATA ANALYSIS.20

**3 OUTCOMES ................................................................................... ..............**  **...... 20th**

3.1 PRIMARY OUTCOME .................................................................... ............. .. 20

3.2 SECONDARY OUTCOMES ................................................................. ............. .... 20th

**4 SCHEDULE21**

**5 BUDGET22**

**REFERENCES23**

ANEXOS28

APPENDIXS45

**1 INTRODUCTION**

Coronavirus disease 2019 (COVID-19), caused by coronavirus 2 Aguda Grave Respiratory Syndrome (SRAG) (SARS-CoV-2) exceeded the global number of 394,381. 395 confirmed cases by February 7, 2022, with more than 5. Seventy-five. 170 reported deaths (WHO, 2022). Researchers estimated that about 81% of confirmed cases of COVID-19 are mild, with a usual recovery period of 2 weeks, 14% progressing to severe pneumonia and 5% developing SRHS, sepsis, and/or multisystemic organ failure (WU; MCGOOGAN, 2020), with the elderly being most severely affected and presenting a lethality of 8-15% (ZHOU et al., 2020).

The lungs of an infected person are the most affected organs because the virus accesses host cells through Enzima Conversora de Angiotine 2 (ACE2), which is most abundant in type II alveolar cells. after fever (HUANG et al., 2020). Nevertheless, extrarespiratory manifestations such as cardiovascular, gastrointestinal, neurological, hepatic, renal, cutaneous and hematological manifestations can also be observed in patients, contributing to complications and various repercussions after the acute phase of the disease (LAI et al., 2020).

The evaluation of patients with an average of 60 days after the onset of first symptoms showed that only 12.6% were completely free of any covid-19-related symptoms, while 32% had 1 or 2 symptoms and 55% had 3 or more. Piora in quality of life was observed in 44.1% of patients. Most individuals also reported fatigue (53.1%), dyspnea (43.4%), joint pain (27.3%) and chest pain (21.7%). This study showed that of the patients who recovered from COVID-19, 87.4% reported persistence of at least 1 symptom, with fatigue and dyspnea being the main ones (CARFI et al., 2020).

A systematic review that sought to improve physical function and fitness results in people infected with Coronavirus related to SARS-CoV noted that physical function and physical strength are impaired after infection, and deficiencies can persist for up to 1 to 2 years. Considering the similarities in the pathology and clinical presentation of SARS-CoV and COVID-19, it is likely that patients with COVID-19 have similar deficiencies in physical function (ROONEY; Webster; PAUL, 2020). These findings may serve to understand the potential deficiencies and rehabilitation needs of people recovering from the current COVID-19 outbreak.

A 1-year follow-up study of 96 patients after COVID-19 who had outpatient care or hospitalization showed that at 12 months the most frequently reported symptoms were reduced exercise capacity (56.3%), fatigue (53.1%), dyspnea (37.5%), concentration difficulties (39.6%), memory (32.3%) and sleep (26%) (SEEBLE et al., 2021).

Post-COVID-19 syndrome may be associated with chronic subclinical systemic inflammation, as can be observed in the aging process. This "inflammation" may have the potential to worsen existing comorbidities and exacerbate age-related problems. Studies evaluating tomographic follow-up of patients who survived SARS and MERS show persistence of pulmonary sequelchanges in a large proportion of cases. An analysis with the follow-up of patients for 15 years showed that 38% of patients persisted with frosted glass opacities or linear consolidations at follow-up (ZHANG et al., 2020). In the evaluation of patients one year after MERS-CoV infection, sequel tomographic alterations were observed in 63% of cases of mild pneumonia and 95% of cases of severe pneumonia (PARK et al., 2018). In the case of SARS, lasting consequences in the form of restrictive ventilatory disorder or lung disease should be expected in at least 25% of survivors (BURNHAM et al., 2013).

Patients with SARs-OV presented a mild or moderate restrictive pattern consistent with muscle weakness in 6% to 20% of individuals in the pulmonary function test performed 6 to 8 weeks after hospital discharge, with persistent impairment of pulmonary function in about one third of patients within 1 year of follow-up. The health status of these SARS survivors was also significantly worse compared to the healthy population, abnormal findings on chest X-ray, as well as persistent reductions in exercise capacity in 12 months and deficit in musculoskeletal performance and QOL. Considering this information, respiratory complications should be considered in patients after COVID-19, as patients may present some degree of impairment and functional limitation due to decreased respiratory function (BARKER-DAVIES et al., 2020).

Initial data from a tomographic follow-up of 59 patients after COVID-19 one month after hospital discharge showed that 39% had signs of residual fibrosis, defined as parenchyma bands, irregular interfaces and bronchiectasis. These were older age, greater extent of involvement in initial tomography, longer hospital stay and higher proportion of hospitalization in an intensive care unit (WEI et al., 2020).

A recent follow-up study of sars surviving patients showed that, three years after infection, 21.74% of the patients evaluated exhibited restrictive ventilatory disorder and 34.78% reduced diffusion capacity to carbon monoxide (DLCO). Fifteen years later, none had restrictive disorder, but 38.36% had a reduction in DLCO15. Similar data are observed in patients who have been affected by MERS-CoV; one year after infection, 37% of patients had a reduction in DLCO and 8% in forced vital capacity (FVC). The evaluation of pulmonary function in patients with COVID-19 at discharge revealed that 47.2% of patients had a reduction in DLCO, 25% reduction in total lung capacity (TLC), 13.6% reduction in expired volume in the first second (FVC1) and 9.1% in FVC. The reduction of CHD was more common in patients with severe pneumonia (OM et al., 2020).

One of the main respiratory symptoms presented in the post-COVID-19 is dyspnea, which may be related to the reduction of respiratory muscle strength. The performance of respiratory muscles can be affected by several factors, such as aging, obesity, sedentary lifestyle, smoking and chronic diseases. In patients with chronic lung diseases, in addition to the reduction of respiratory muscle strength, the demand imposed on the respiratory muscle also increases due to changes in airway resistance and chest wall mechanics. An analysis of patients post-COVI-19 at hospital discharge revealed that more than one third of the recovered patients develop fibrotic abnormalities, which increases the pressure needed to breathe. Long-term pulmonary involvement may develop after the elimination of the virus and, in particular, fibrotic interstitial lung disease, which may be related, in addition to other causes, to chronic inflammation caused by COVID-19 (VASARMIDI et al., 2020). Therefore, patients suffering from acute viral infection may present additional imbalances between the ability to generate respiratory muscle strength and the demands imposed for breathing, increasing the risk of respiratory failure (SEVERIN et al., 2020).

Viral respiratory diseases are also associated with acute and long-term psychopathological consequences in survivors (BOHMWALD et al., 2018). Patients with COVID-19 may present with delirium, anxiety, depression and insomnia, induced by psychopathological sequelae through direct viral infection of the central nervous system (CNS) by coronavirus or indirectly through an immune response (WU et al., 2020).

A survey that tracked psychiatric symptoms in 402 adult covid-19 survivors showed that 28% of patients self-rated for Post Traumatic Stress Disorder, 31% for depression, 42% for anxiety, 20% for obsessive compulsive disorder symptoms and 40% for insomnia. In addition, the Basal Systemic Immune Inflammation Index was positively associated with depression and anxiety scores in the follow-up of these patients (MAZZA et al., 2020). Therefore, it is recommended to evaluate the psychopathology of patients surviving COVID-19 to refer when necessary for diagnosis and treatment of emerging psychiatric conditions. The Stanfort Consensus recommends the commending of these patients to identify adverse psychological effects as a result of COVID-19 and referral to psychological services (BARKER-DAVIES et al., 2020).

- 1. JUSTIFICATION

There is sufficient evidence for a possible post-COVID-19 syndrome, designating sequelae with persistent symptoms. The ATS recommends that patients after COVID-19 should undergo an assessment of physical and emotional function, respiratory function, exercise capacity 6 to 8 weeks after hospital discharge and those in need of interventions should receive a comprehensive rehabilitation program according to their initial assessment (SPRUIT et al., 2020).

Respiratory muscle training improves respiratory muscle strength, exercise capacity, diaphragm muscle thickness, and dyspnea in several patient populations, especially those with greater reduction in basal respiratory muscle strength. The effects of respiratory muscle training were observed in protocols lasting only 4 weeks, facilitating training adhering (SEVERIN et al., 2020).

In view of this information, we can see that research in this context is necessary to measure the extent of functional impairments in patients post-COVID-19. In addition, research should evaluate whether rehabilitation interventions can promote the improvement of post-infection symptoms (SPRUIT et al., 2020).

As an essential component of post-acute care, rehabilitation aims to reduce long-term disability and allows patients to live in the community and return to their previous level of social participation. Ter a well-designed post-COVID-19 rehabilitation program to respond to the need for care and ensure the effectiveness, efficiency and adequacy of rehabilitation treatment represents an appropriate strategy (VOURGANAS; STANKOVIC; 2021).

Socioeconomic and health reasons in the post-COVID-19 world require home rehabilitation without direct assistance from a professional (VOURGANAS; STANKOVIC, 2021). The need for social distancing, the vulnerability of some groups and the restructuring of face-to-face activities required a rapid readaptation in the scope of care for patients in need of rehabilitation, such as unsupervised home care and tele rehabilitation. In addition, easy accessibility to treatment anywhere and the low costs of a home rehabilitation program are factors that can become an interesting and viable strategy to monitor patients in the post-COVID-19 phase.

Previous studies conducted with patients in home training produced short-term clinical results that were equivalent to outpatient pulmonary rehabilitation COPD, also showing efficacy in reducing the risk of acute EXACERBATION of COPD and hospitalizations (VASILOPOULOU et al., 2017), besides being a low-cost alternative for patients (BURGE et al., 2020).

1.2 OBJECTIVES

**1.2.1 Primary endpoint**

Evaluate the efficacy of a home inspiratory muscle training protocol in improving respiratory muscle strength, dyspnea and quality of life of patients after COVID-19.

**1.2.2 Secondary objectives**

Evaluate after IMT the influence of:

- Pulmonary function;
- Perception of exertion and dyspnea;
- Exercise tolerance;
- Quality of life;
- Peripheral muscle strength;
- Anxiety and depression;
- Functional status.

1.3 HYPOTHESIS

The hypothesis arises that the home inspiratory muscle training protocol can be effective and safe to improve respiratory muscle strength, dyspnea, quality of life, pulmonary function, perceived effort, exercise tolerance, peripheral muscle strength, anxiety and depression and functional status of patients post-covid-19, generating a good support of the participants to treatment.

**2 MATERIALS AND METHODS**

2.1 Search Type

This is an experimental trial of the randomized, controlled and blind clinical trial that will be conducted at the Institute of Tropical Medicine of the Federal University of Rio Grande do Norte (UFRN).

2.2 Research population

Subjects of both genders, aged over 18 years, with a confirmed diagnosis of COVID-19, will be recruited by the Infectious Diseases Outpatient Clinic of the Giselda Trigueiro Hospital of the State Department of the Municipality of Natal- Rio Grande do Norte (HGT/SESAP-RN).

2.3 Sample size

The sample size will be performed using the GPower software version 3.1.9.2 (Kiel, Germany) for Windows and will be established after a pilot study with 5 participants in each group (total of 10 subjects) for a hypothetical two-way ANOVA test, using the main variable the Maximum Inspiratory Pressure (Max), in which the mean and standard deviation will be used to estimate the size of the effect, being adopted an alpha error of 0.05 with bilateral distribution and a test power of 80%. A larger sampling N will be established considering a loss of 20%.

2.4 Inclusion and exclusion criteria

Subjects of both sexes, sedentary, with a diagnosis of COVID-19 confirmed by RT-PCR, aged over 18 years and without any basic respiratory disease, with adequate cognitive status defined through the Mini Mental State Examination (EMMS) (APPENDIX 1) and reduction of respiratory muscle strength, defined through the assessment of the MIP. according to the following prediction equations: homens (153.3 - 0.8 x age) and women (110.4 - 0.49 x age) and considering the lower threshold of normality with standard estimation error (PEE) for men of 17.3 and women of 9.1 (NEDER et al., 1999).

Subjects who present some condition that make it impossible to perform the evaluations and protocols, complications that justify the interruption of data collection, such as syncope, severe chest pain, coughing with blood, those who request departure from the study and that present adverse effects such as hospitalization for sharpening the clinical picture, will be excluded from the study.

2.5 Procedures for selecting subjects

For patient recruitment, researchers will contact the HGT team. All patients contacted will be informed about the research and its importance. Participation in the research will be conditional on the signing of the Free and Informed Consent Form (TCLE) (APPENDIX A).

The sample will be randomized automatically and allocated in two distinct groups through the www.randomization.com website, determined as Experimental Group (EG): inspiratory muscle training and Control Group (G2): placebo IMT without any load. The groups will be encoded and the allocation will be transferred to a series of sealed and sequentially numbered opaque envelopes.

Three evaluators will participate in the research: researcher 1 will be responsible only for the evaluations, researcher 2, load adjustment and researcher 3, for randomization. The study will be double-blind, because researcher 1 and participants will not be aware of the allocation of the subjects in the groups, nor of the effects of the intervention.

2.6 Ethical Aspects

For its implementation, this project was submitted to the Research Ethics Committee (CEP) of the Federal University of Rio Grande do Norte (CAAE) (CAAE**:** 45555421.7.0000.5537) and submitted to clinical trials (NCT05077241) . The autonomy and guarantee of anonymity of the participants will be respected, ensuring their privacy regarding confidential data, as governed by Resolution 510/16 of the National Health Council and the Helsinki declaration for research with human beings. Before admission to the study, all volunteers will sign the Informed Consent Form.

2.7 Study Design

The subjects included in the research will go through three evaluation moments: Pre-training (Initial), Post-Training (6 weeks) and Retention Test (24 weeks).

After recruitment, participants will be invited to attend the Institute of Tropical Medicine to perform an initial evaluation by a previously trained and blind evaluator for the intervention allocation group and will include anamnesis and physical examination, with measurement of vital signs, anthropometric measurements, evaluation of lung volumes, respiratory muscle strength, peripheral muscle strength, quality of life, anxiety and depression, functional status and 6-minute walk test.

After the initial evaluation, all volunteers will receive a POWERbreathe device® (POWERbreathe®, HaB Ltd, Southam, UK) for training, and will be guided individually on how to use it and how to carry out the protocol. They will conduct an experimental session for familiarization with the device that will not be considered for analysis. Every three days the volunteers will receive a telephone call from researcher 2 who will not participate in the evaluation to confirm whether the exercise with POWERbreathe® was being performed properly in the frequency and load oriented and if there was any doubt regarding the protocol. At the end of each week participants will receive a video call from researcher 2 to perform the adjustment on the device according to the weekly load progression of G1.

All evaluation moments (pre-training, post-training and retention test) will be performed by a single evaluator (Evaluator 1) – who will not know in which group the subject will be allocated – and recorded in the evaluation form developed for the project. A second researcher (Evaluator 2) will be responsible for applying the training protocols to the subjects.

- 1. Data collection procedure

*2.8.1 Clinical Evaluation Form*

After signing the TCLE, an evaluation of the research volunteers will be carried out by means of an evaluation form (APPENDIX B). This form will have the purpose of directing the collection of personal data, vital signs, anthropometric measurements (weight, height, body mass index), personal and pathological history, life habits, physical examination (inspection and palpation) and complementary tests.

*2.8. 2 Anthropometric measurements*

For the evaluation of anthropometric profile will be considered the following variables: Body Mass (MC), height, Body Mass Index (BMI) and Calf Perimeter (PP).

Participants will be weighed on a *filizola®* model 31 digital scale (Filizola®, São Paulo - SP, Brazil), with accuracy of 100 grams, measured and certified by the National Institute of Metrology, Standardization and Industrial Quality (INMETRO), with light clothes, barefoot, upright, with feet together, arms extended along the body, positioned, backto-back to measure the scale, remaining still with a stare in front of the reading (BRASIL, 2004).

They will have their height measured by a statometer integrated into the scale, graduated in centimeter and accuracy of 1 millimeter, during maximum inspiration and should be in orthostatism, with arms extended along the body, cervical spine aligned, looking at a fixed point at eye height, with the heels, shoulders and buttocks in contact with the wall, the internal maleolos touching each other, as well as the inner part of both knees (BRASIL, 2004).

Body Mass Index (BMI) will be calculated considering the ratio between body mass and height square (kg/m²), being classified by a graph according to age in low weight (BMI ≦ 22); eutrophic (BMI > 22 and < 27), overweight (BMI ≧ 27), according to the Ministry of Health (2017).

*2.8.3 Respiratory* *muscle força*

The evaluation of respiratory muscle strength will be performed by measuring maximum respiratory pressures (Maximal Inspiratory Pressure: _PImax_ and Maximum Expiratory Pressure: _PEMAX_) through the use of the analog manovacuometer (GERAR^®^, São Paulo, Brazil) with an operational interval of ± 300 cmH2 The and equipped with oral adapter, containing an exhaust valve through a hole approximately 2mm in diameter to prevent increased intraoral pressure.

Participants should use a nasal clip to avoid air outing, sitting in a chair, with the back supported, hip flexion at 90 degrees, upper limbs resting on the thighs and feet supported on the floor, so they feel comfortable and relaxed.

To measure the _PImax,_ the participant must perform the expiration until reaching his Residual Volume (VR), immediately connecting the oral part in the oral route and performing a maximal inspiratory effort against the occluded airway, sustaining for at least one second. To measure the _PEmax,_ the participant must inspire until reaching his Total Lung Capacity (TLC) and then inserting the oral part into the oral cavity, he/she will perform a maximum expiratory effort against the occluded airway, and should be maintained for at least one second (ATS, 2002). During the expiratory effort of the patient, the evaluator will maintain a digital pressure around the lips, in order to prevent air escape. Participants will receive constant encouragement from the evaluator during the test.

For data analysis, at least three reproducible maneuvers will be used, with variability less than 20%, and the highest value will be recorded. The references of normality for the calculation of maximal respiratory pressures according to age and gender will respect the description of the method used by Neder et al. (1999) for the Brazilian population.

*2.8.4 Lung Volumes and Capacities*

All participants will be evaluated by a *Koko Digidoser model spirometer* (*Spide*, *Longmont*, USA) to check lung volumes and capacities. All tests will be performed by the same evaluator using the previously mentioned spirometer, previously calibrated and in an air-conditioned environment.

Individuals were instructed to avoid bulky meals one hour before the test, not to consume foods or beverages containing caffeine for at least six hours before, and not to drink alcoholic beverages on the day of the test (ATS, 2019).

The test consisted of an inspiration albeit maneuver up to total lung capacity (TLC), followed by a maximum forced expiration to the residual volume (RV), performed through the device. The tests were performed in the sitting position with flexion of hips and knees at 90°, according to the criteria previously established by *the American Thoracic Society* (ATS, 2019). At least three tests will be performed, with a variation of less than 5% and the highest value obtained in one of the tests will be compared with the predicted values of pulmonary function parameters for the Brazilian population. Vef1 and FVC _will_ be evaluated. The predicted values will be calculated using the reference values (PEREIRA et al., 2007).

*2.8.5 Dyspnea Assessment*

Dyspnea will be evaluated using the scale adapted to Portuguese of Medical Research Council (APPENDIX 2). The scale will allow the volunteer to indicate the extent to which his shortness of breath affected his mobility in daily activities. The scores range from 1 to 5, whose higher values refer to greater dyspnea, and will be elected by the participant (BESTALL et al., 1999).

*2.8.6 Value* *of Perception of Effort and Fatigue*

Before and after training individuals will be questioned separately as to their subjective perception of exertion during breathing and fatigue in the lower limbs. To quantify was will be the modified Borg scale (ANNEX 3). The scale presented numerical values from 0 to 10 and expressions that classify the degree of difficulty, whose higher values reflect a greater sensation of shortness of breath. This scale is commonly used to measure discomfort, exertion, fatigue at rest and during exercise, since the measurement is done directly at the moment the individual is experiencing the sensation (BORG, 2007).

*2.8.*  *7 Handgrip strength assessment*

Handgrip strength will be measured with manual hydraulic dynamometer (Saehan®). It will be quantified by measuring the amount of maximum isometric force that the dominant hand can tighten the dynamometer. The participant will be comfortably seated in a chair, knees fletidos to 90º shoulder of the dominant arm adduced and neutrally rotated, elbow flegated at 90º and next to the trunk, forearm in neutral position and wrist between 0º and 30º of extension and 0º to 15º of ulnar deviation. Participants will be asked to tighten the dynamometer with maximum isometric force, without any other body movement, for 5 seconds (NOVAES et al., 2009). This measurement will be performed three times, with one minute interval between them, considering the mean for analysis (PEREIRA et al., 2009). Those whose mean of the three measurements is among the 20% lowest values of the distribution, with adjustment by gender and body mass index (BMI - weight/^height2^), according to the ranges suggested by the WHO (men: 0 < BMI ≤ 23, cut-off point (CP) ≤ 27.00kgf; 23 < BMI < 28, PC ≤ 28.67kgf, 28 ≤ BMI < 30, PC ≤ 29.50; BMI ≥ 30, PC ≤ 28.67; women: 0 < BMI ≤ 23, PC ≤ 16.33; 23 < BMI < 28, PC ≤ 16.67; 28 ≤ BMI < 30, PC ≤ 17.33; BMI ≥ 30, PC ≤ 16.67) (MARUCCI; BARBOSA, 2003).

*2.8.8 Six-Minute Walk Test (6MWT)*

The 6MWT evaluates the submaximal level of functional capacity during exercise, measuring the distance that a patient can walk on a flat surface of 30 meters for six minutes. For the realization, the participant will be instructed to walk at maximum sustained speed, but without running, being possible to stop to rest and return to the test, when necessary. The evaluator will monitor HR, _SpO2_, perception of dyspnea (Borg-D) and fatigue (Borg-F) of lower limbs (BORG, 1982) at each turn and participants will be verbally stimulated every minute according to the phrases standardized by the test recommendations. At the end of the six minutes the number of laps will be recorded and a marker placed on the ground next to the subject (ATS, 2002).

The distance traveled on the last lap will be measured by a measuring tape and the total distance obtained by multiplying the number of laps by the measure of space and adding the distance traveled from the last lap (ATS, 2002). The method to evaluate the predicted distance will follow the equations proposed in the literature, based on gender, weight, age and height of the participants (ENRIGHT; SHERRILL, 1998).

BP, HR, SatO2, Borg-D and Borg-F will also be evaluated at the beginning and end of the test.  If participants present with chest pain, intolerable dyspnea, spo2 fall below 85%, muscle cramps, sweating, pallor and/or vertigo, the test will be interrupted (ATS, 2002).

*2.8.*  *9 Evaluation of cognition*

The Mini Mental State Examination (MMSE) will be used for cognitive screening in the elderly. It consists of two sections that assess cognitive functions. The first section contains items that assess orientation, memory and attention, totaling 21 points; the second is the ability to appoint, obey a verbal command and a writing, free writing of a sentence and copying a complex drawing (polygons), totaling 9 points. The final score is 30 points. The highest values of the score indicate higher cognitive performance (FOLSTEIN et al., 1975). The proposed cut-off scores will be used to minimize the influence of education level on total scores (BERTOLUCCI et al., 1994).

The participant should be left at ease, and should not feel judged. Any mistakes made by him during the application should not be corrected, so as not to inhibit them. A point will be added for each correct and zero answer for the wrong or unanswered answers.

*2.8.10* *Study of Medical Outcomes 36-Item Short Health Form Research (SF-36)*

The *Medical Outcomes Study 36-Item Short Health Form Survey (SF-36) (APPENDIX 4) is* a multidimensional questionnaire translated and validated for the Brazilian reality (CICONELLI et al., 1999; LAGUARDIA et al., 2011) that was developed to assess health-related quality of life. The instrument consists of 36 items, distributed in 8 domains that encompass two major components: physical aspects (physical aspects, body pain, general state of health and physical function) and mental (emotional aspects, social function, mental aspects and vitality). Scores range from 0 to 100 for each subscale, with the highest scores indicative of a better health-related quality of life (WARE; SHERBOURNE, 1992).

*2.8.11 International Physical Activity Questionnaire (IPAQ)*

The long version of IPAQ (ANNEX 5), adapted (MAZO; BENEDETTI, 2001) and validated for the Brazilian population (BENEDETTI et al., 2008) is composed of 5 domains and 15 questions and was developed to evaluate the weekly energy expenditure of physical activities related to work, transportation, household chores and leisure. The instrument considers the activities practiced for at least 10 continuous minutes, with moderate and vigorous intensity, performed in the previous week.

It will be applied as an interview by previously trained evaluators and the elderly will be instructed to answer the questionnaire based on a usual week. At the end, the interviewer should add the times (minutes and hours/day) and write down in each question the total value in minutes and hours and days of the week. You should then sum up the totals of each domain and thus calculate the total of all physical activity in minutes per week. Energy expenditure should be calculated considering the minutes per week for each activity estimated in METs (BENEDETTI et al., 2008), using the compendium of Ainsworth et al. Participants will be classified as very active, active, irregularly active and sedentary (MATSUDO et al., 2002).

*2.8.12 Scale of depression and anxiety*

Depression self-assessment (SDS) and anxiety self-assessment scale (SAS) (APPENDIX 6) will be explored to assess depression and anxiety in the groups. Both the SDS and the SAS have 20 items, each of which will be scored on a scale of 1–4, and the higher the score, the more severe the degree of depression and anxiety (ZUNG, 1991).

*2.8.13 Post-Covid-19 functional status scale*

The post-covid-19 functional status scale (APPENDIX 7) assesses relevant aspects of daily life during follow-up after infection. The scale is intended to help users become aware of current functional limitations in patients with Covid-19 and to objectively determine the degrees of functional limitations. The scale contains six items ranging from zero to five and comprises the full range of functional outcomes focusing on task limitations and activities of daily living (household, work/study and lifestyle changes). The general classification corresponds to the worst functional status indicated by the patient responses (the highest degree corresponding to the highest limitations) (KLOK et al.,2020).

*2.8.14 Assessment of adverse effects and*

For the assessment of adverse effects, a training journal (APPENDIX C) will be provided for all study volunteers, in which they will be willing every training day they must do and a blank space for positive and/or negative observations and to record any interrunence during and/or after training sessions. For analysis of training adhering will be considered every time they score in the tables signaling the session. The sum of all sessions will be held and esthes will be divided by the total number of sessions that participants must do.

The analysis of adverse effects will be performed considering all the complications recorded in the journal or commented during the final evaluations.

- 1. RESEARCH INTERVENTION PROTOCOL

When randomized, the subjects will be allocated into two groups (GE and GC). The following training protocols will be adopted for the respective groups:

GE Protocol: TMI with 30% of Pimmax. with a weekly load increase of 10% of the MIP value. initial. The sessions will consist of 30 repetitions, 2 times a day, once in the morning and once in the afternoon, 7 consecutive days a week, for 6 weeks. Individuals will be instructed to perform a rapid contraction of the inspiratory muscles and sustain it for 2 seconds, in each maneuver, and will have the possibility to rest every 3 repetitions of the IMT for 30 seconds to avoid muscle fatigue or any other interfacing.

GC Protocol: Individuals will use a TMI device without any charge and will receive the same guidance as G1. At the end of the research, the control group will be entitled to experimental treatment with the IMT protocol, if it is effective.

The research will be funded by the researchers and that the devices will be provided free of charge to the study participants.

- 1. RISKS

This research presents minimal risks to participants. They may feel constrained during the interview, considering the nature of the content of some questionnaires, which they may refuse to answer, or because of the difficulty in performing any specific evaluation. They may present some discomforts such as headache, vertigo, nausea, blood pressure oscillation and even a small imbalance due to the requirement of certain physical tests and training, however the evaluators will use contraindication criteria or interruptions of the tests when necessary, to minimize the risks to the health of the participants. The participant may refuse to perform any procedure at any time, without prejudice to him.

In addition, they may also present concerns about the confidentiality of the data collected and the signature they must make in the TCLE. The researcher will assist them by clarifying all existing doubts and inform the participants that the collected data will be used only for academic purposes, and that there will be no disclosure of them.

- 1. BENEFITS

The results obtained with the development of this research can guarantee the possibility of expanding interventions related to pulmonary rehabilitation in post-covid-19 patients, through effective, accessible, safe and low-cost training. The information collected may also contribute to the screening of patients after the period of acute infection, contributing to the understanding of about this new theme, the development and evaluation of the effectiveness of interventions aimed at improving the health of this population.

- 1. DATA ANALYSIS

For data analysis, the SPSS (*Statistical Package for the Social Sciences)*  version 22.0 software for Windows will be *used*. The normality test will be performed according to the number of volunteer participants of the research. Therefore, the Shapiro-Wilk normality test or the Kolmorov-Smirnov normality test for the desired variables may be used.

The variables that present non-parametric distribution will be compared through the Wilcoxon (intragroup analysis) and Mann-Whitney (intergroup analysis) tests and when the distribution occurs parametricly, the two-way ANOVA test will be used. When there is significant difference, Dunn's *post hoc* test will be applied in order to locate the differences.

In the descriptive analysis, a characterization of the studied population will be performed, by obtaining the means and standard deviations for variables of normal or median distribution and interquartile interval (25%-75%) for variables of asymmetric distribution. To minimize a possible type I error, the significance level of 5% will be established. The power of the study and the size of the effect will be exposed in the main results of the study.

**3 OUTCOMES**

- 1. PRIMARY OUTCOMES

Respiratory muscle strength, dyspnea and quality of life will be considered as primary outcomes.

3.2 SECONDARY OUTCOMES

Secondary outcomes will be considered: pulmonary function, exercise tolerance, functional status, anxiety and depression, peripheral muscle strength, adverse effects and ades.

**4 SCHEDULE FOR THE EXECUTION OF ACTIVITIES**

| **Activities 2021** | **Jan** | **Feb** | **March** | **Apr** | **May** | **Jun** | **Jul** | **Behind** | **Put** | **Outside** | **November** | **Ten** |
| --- | --- | --- | --- | --- | --- | --- | --- | --- | --- | --- | --- | --- |
| **Literature review** |  |  |  |  |  |  |  |  |  |  |  |  |
| **Design of the project** |  |  |  |  |  |  |  |  |  |  |  |  |
| **Submission of the project to the Brazil Platform** |  |  |  |  |  |  |  |  |  |  |  |  |
| **Data collection** |  |  |  |  |  |  |  |  |  |  |  |  |

| **Activities 2022** | **Jan** | **Feb** | **March** | **Apr** | **May** | **Jun** | **Jul** | **Behind** | **Put** | **Outside** | **November** | **Ten** |
| --- | --- | --- | --- | --- | --- | --- | --- | --- | --- | --- | --- | --- |
| **Data collection** |  |  |  |  |  |  |  |  |  |  |  |  |

| **Activities 2023** | **Jan** | **Feb** | **March** | **Apr** | **May** | **Jun** | **Jul** | **Behind** | **Put** | **Outside** | **November** | **Ten** |
| --- | --- | --- | --- | --- | --- | --- | --- | --- | --- | --- | --- | --- |
| **Data collection** |  |  |  |  |  |  |  |  |  |  |  |  |
| **Data analysis** |  |  |  |  |  |  |  |  |  |  |  |  |
| **Writing of manuscripts** |  |  |  |  |  |  |  |  |  |  |  |  |
| **Defense** |  |  |  |  |  |  |  |  |  |  |  |  |

**5 BUDGET**

| **Consumption material (Costing)** | **Value (R$)**  **Total** |
| --- | --- |
| Printing service | 500,00 |
| Translation services | 400,00 |
| Transport | 500,00 |
| Pens  Espirômetro (1)  Manovacuômetro (1)  Dynamometer (1)  Energy breathing (10)  Scale (1) | 30,00  27.000,00  1.700,00  5.000,00  5.000,00  50,00 |
| **Total Expenses** | **40.180,00** |

**REFERENCES**

AINSWORTH, Barbara E. et al. Compendium of physical activities: an update of activity codes and met intensities.  **Medicine and science in sport and exercise**, v. 32, n. 9; SUPP/1, p. S498-S504, 2000.

BARKER-DAVIES, Robert M. et al. Stanford Hall's consensus statement for post-COVID-19 rehabilitation. **British Journal of Sports Medicine**, v. 54, n. 16, p. 949-959, 2020.

BARKER-DAVIES, Robert M. et al. Stanford Hall's consensus statement for post-COVID-19 rehabilitation. **British Journal of Sports Medicine**, v. 54, n. 16, p. 949-959, 2020.

BENEDETTI, Tania B.; MAZO, Giovana Z.; DE BARROS, Mauro VG. Application of the international physical activities questionnaire to assess the level of physical activity of older women: Concurrent validity and test-retest reproducibility.  **Revista Brasileira de ciência e movimento**, v. 12, n. 1, p. 25-34, 2008.

BERTOLUCCI, Paulo HF et al. The mini-examination of mental status in a general population: impact of schooling. **Archives of Neuropsychiatry**, v. 52, n. 1, p. 01-07, 1994.

BESTALL, J.C. et al. Usefulness of the medical research council(MRC) dyspnoea scale as a measure of disability in patients with chronic obstructive pulmonary disease. **Thorax**, v. 54, n. 7, p. 581-586, 1999.

BOHMWALD, Karen et al. Neurological changes due to respiratory virus infections. **Frontiers in cellular neuroscience**, v. 12, p. 386, 2018.

BORG E. About perceived effort and its measurement. **Psychology, psychology**. 2007. 1927-34.

BURGE, Angela T. et al. Home pulmonary rehabilitation for COPD using minimal resources: An economic analysis. **Respirology**, v. 25, n. 2, p. 183-190, 2020.

BURNHAM, Ellen L. et al. The characteristics of computed tomography of the breast are associated with worse quality of life in survivors of acute lung lesions. **Critical care medicine**, v. 41, n. 2, p. 445, 2013.

CARFÌ, Angelo et al. Persistent symptoms in patients after acute COVID-19. **Jama**, v. 324, n. 6, p. 603-605, 2020.

CICONELLI, Rozana Mesquita et al. Translation into Portuguese and validation of the generic quality of life assessment questionnaire SF-36 (Brasil SF-36). **Rev bras rheumatol**, v. 39, n. 3, p. 143-50, 1999.

ENRIGHT, L. Paul; SHERRILL, Duane L. Reference equations for six-minute walk in healthy adults. **American Journal of Respiratory and Critical Medicine**, v. 158, n. 5, p. 1384-1387, 1998.

FOLSTEIN, Marshal F.; FOLSTEIN, E. Susan; MCHUGH, Paul R. "Mini-mental state": a practical method to classify the cognitive state of patients to the doctor. **Psychiatric Research Journal**, v. 12, n. 3, p. 189-198, 1975.

GRAHAM, Brian L. et al. Standardization of the update of spirometry 2019. An official American thoracic society and a technical declaration of the European respiratory society. **American Journal of Respiratory and Critical Medicine**, v. 200, n. 8, p. e70-e88, 2019.

HOLLAND, Anne E. et al. An official technical standard of the European Respiratory Society/American Thoracic Society: field walking tests on chronic respiratory diseases. **European Respiratory Journal**, v. 44, n. 6, p. 1428-1446, 2014.

HUANG, you-Chering; LEE, Ping-Ing; HSUEH, Po-Ren. Evolving COVID-19 reporting criteria in Taiwan during the epidemic. **Journal of Microbiology, Immunology and Infection**, v. 53, n. 3, p. 413-418, 2020.

KLOK, Frederikus A. et al. The post-COVID-19 functional status scale: a tool for measuring functional status over time after COVID-19. **European Respiratory Journal**, v. 56, n. 1, 2020.

LAGUARDIA, Joshua et al. Psychometric evaluation of the SF-36 questionnaire (v. 2) in a probability sample of Brazilian households: results of the Survey Survey Social Dimensions of Inequalities (PDSD), Brazil, 2008. **Health and Quality of Life Results,** v. 9, n. 1, p. 61, 2011.

LAI, Chih-Cheng et al. Extrarespiratory manifestations of COVID-19. International journal of antimicrobialagents, v. 56, n. 2, p. 106024, 2020.

MARUCCI, Maria de Fátima Nunes et al. Nutritional status and physical capacity. **The SABE Project in the city of São Paulo: an initial approach. Brasilia: PAHO/MS**, p. 95-117, 2003.

MATSUDO, Sandra Mahecha et al. Level of physical activity of the population of the State of São Paulo: analysis according to gender, age, socioeconomic level, geographic distribution and knowledge. **Brazilian journal of science and movement**, v. 10, n. 4, 2008.

MAZO, Giovana Zarpellon et al. Concurrent validity and reproducibility: test-retest of the modified Baecke Questionnaire for the elderly. **Revista Brasileira de Atividade Física & Saúde**, v. 6, n. 1, p. 5-11, 2001.

Mazza, Mario Gennaro et al. Anxiety and depression in SURVIVORS OF COVID-19: Role of inflammatory and clinical predictors. **Brain, behavior and immunity**, v. 89, p. 594-600, 2020.

MINISTRY OF HEALTH (Brazil). Food and Nutrition Surveillance System. Basic operations for the collection, processing, analysis of data and information in health services. Brasília, DF: Ministry of Health; 2004.

MINISTRY OF HEALTH. **Evaluation of BMI weight in the elderly.** May 30, 2017.

MO, Xiaoneng et al. Abnormal pulmonary function in COVID-19 patients at hospital discharge. **European Respiratory Journal**, v. 55, n. 6, 2020.

NEDER, José Alberto et al. Reference values for pulmonary function tests: II. Maximal respiratory pressures and voluntary ventilation. **Brazilian Journal of Medical and Biological Research**, v. 32, n. 6, p. 719-727, 1999.

NOVAES, Rômulo Dias et al. Reference equations for the prediction of handgrip strength in middle-aged Brazilians and the elderly. **Physiotherapy and** Research, v. 16, n. 3, p. 217-222, 2009.

ORGANIZATION OF HEALTH. WHO Coronavirus Panel (COVID-19). 2022. Removed from: https://covid19.who.int/.

PARK, Wan Beom et al. Correlation between pneumonia severity and pulmonary complications in Middle Eastern respiratory syndrome. **Journal of Korean medical science**, v. 33, n. 24, 2018.

PEREIRA, Carlos Alberto de Castro; SATO, Taeko; RODRIGUES, Sílvia Carla. New reference values for forced spirometry in white adults in Brazil. **Jornal Brasileiro de Pulmonology**, v. 33, p. 397-406, 2007.

ROONEY, Scott; WEBSTER, Amy; PAUL, Lorna. Systematic review of Changes and Recovery in Physical Function and Fitness After Severe Acute Coronavirus Infection : Implications for REHABILITATION COVID-19. **Physiotherapy**, v. 100, n. 10, p. 1717-1729, 2020.

SEEßLE, Jessica et al. Persistent symptoms in adult patients 1 year after coronavirus disease 2019 (COVID-19): a prospective cohort study. **Clinical infectious diseases: an official publication of the Society of Infectious Diseases of America,** 2021.

SEVERIN, Richard et al. Screening of respiratory muscle performance for the management of infectious diseases after COVID-19: a highly pressurized situation. **The American Journal of Medicine**, 2020.

SPRUIT, Martijn A. et al. COVID-19: provisional guidance on rehabilitation in the hospital and post-hospital phase of an international task force coordinated by the European Respiratory Society and the American Thoracic Society. **European Respiratory Journal**, v. 56, n. 6, 2020.

VASARMIDI, Eirini et al. Pulmonary fibrosis after the COVID-19 era. **Experimental and therapeutic medicine**, v. 20, n. 3, p. 2557-2560, 2020.

VASILOPOULOU, Maroula et al. Telerehabilitation of home maintenance reduces the risk of acute exacerbations of COPD, hospitalizations and emergency consultations. **European Respiratory Journal**, v. 49, n. 5, 2017.

VOURGANAS, Ioannis; STANKOVIC, Vladimir; STANKOVIC, Lina. Individualized Artificial Intelligence responsible for home rehabilitation. **Sensors**, v. 21, n. 1, p. 2, 2021.

JR WARE, John E.; SHERBOURNE, Cathy Donald. The short-form health research MOS 36 items (SF-36): I. Conceptual structure and selection of items. **Medical care**, p. 473-483, 1992.

WEI, Jiangping et al. Analysis of fine-section tomography in patients with coronavirus disease (COVID-19) after hospital discharge. **Journal of X-ray Science and Technology**, n. Preprint, p. 1-7, 2020.

WU, Yeshun et al. Involvement of the nervous system after infection with COVID-19 and other coronaviruses. **Brain, behavior and immunity**, v. 87, p. 18-22, 2020.

WU, Zunyou; MCGOOGAN, Jennifer M. Important features and lessons from the 2019 coronavirus disease outbreak (COVID-19) in China: a report summary of 72,314 cases from the Chinese Centers for Disease Control and Prevention. **Jama**, v. 323, n. 13, p. 1239-1242, 2020.

ZHANG, Peixun et al. Long-term bone and pulmonary consequences associated with acute acute respiratory syndrome acquired by the hospital: a 15-year follow-up of a prospective cohort study. **Bone research**, v. 8, n. 1, p. 1-8, 2020.

ZHOU, Fei et al. Clinical course and risk factors for mortality of adult hospitalization patients with COVID-19 in Wuhan, China: a retrospective cohort study. **The lancet**, v. 395, n. 10229, p. 1054-1062, 2020.

ZUNG, William W. A classification instrument for anxiety disorders. **Psychosomatic: Journal of Consultation and Liaison Psychiatry**, 1971.

**ANNEXES**

**ANNEX 1 -** Mini Mental State Examination (MMSE)

**ANNEX 2 -**  MRC Dyspnea Scale

| **ENGLISH VERSION OF MEDICAL RESEARCH COUNCIL (MRC) *DISPNEIA*** SCALE | |
| --- | --- |
| **Gray 1** | He only suffers shortness of breath during intense exercise. |
| **Gray 2** | He suffers from shortness of breath when he walks hastily or climbs a light ramp. |
| **Gray 3** | Walk slower than people of the same age because of shortness of breath or have to stop to breathe even when walking slowly. |
| **Gray 4** | Para to breathe after walking less than 100 meters or after a few minutes. |
| **Gray 5** | He's so short of breath, he doesn't leave the house anymore, or when he's getting dressed. |

**ANNEX C -** Borg scale amended

| **MODIFIED BORG SCALE** | |
| --- | --- |
| **0** | No |
| **0,5** | Very, very light |
| **1** | Very light |
| **2** | Lightweight |
| **3** | Moderate |
| **4** | Little intense |
| **5** | Intense |
| **6** |  |
| **7** | Very intense |
| **8** |  |
| **9** | Very, very intense |
| **10** | Maxim |

**ANNEX 3 -** Study of Medical Outcomes 36-Item Short Health Form Research (SF-36)


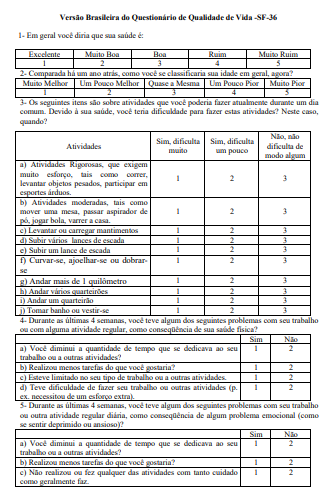


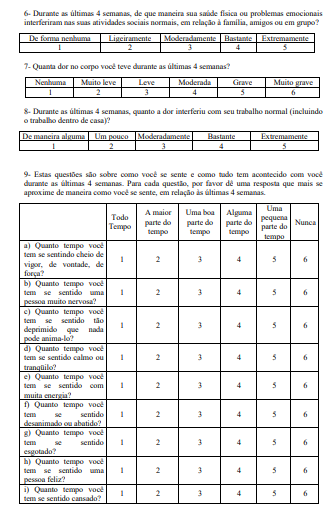


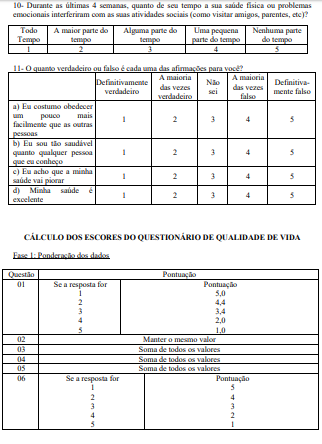


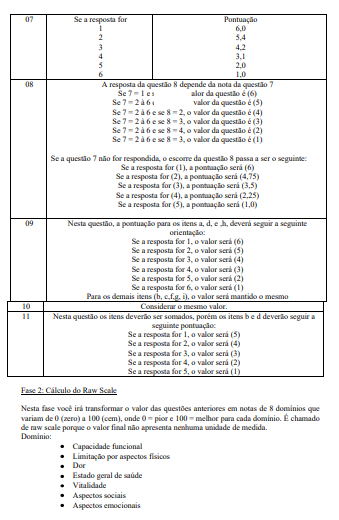


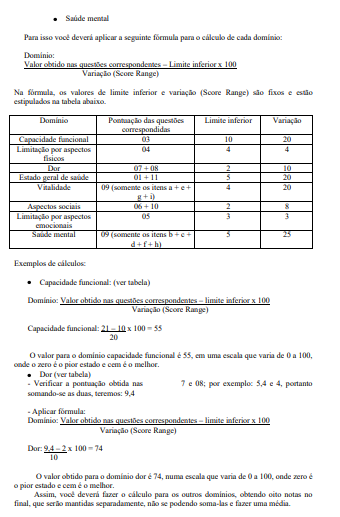


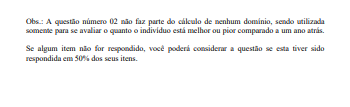


**ANNEX 4 -** *International Physical Activity Questionnaire (IPAQ)*

**ANNEX 5 -** Depression and Anxiety Scale


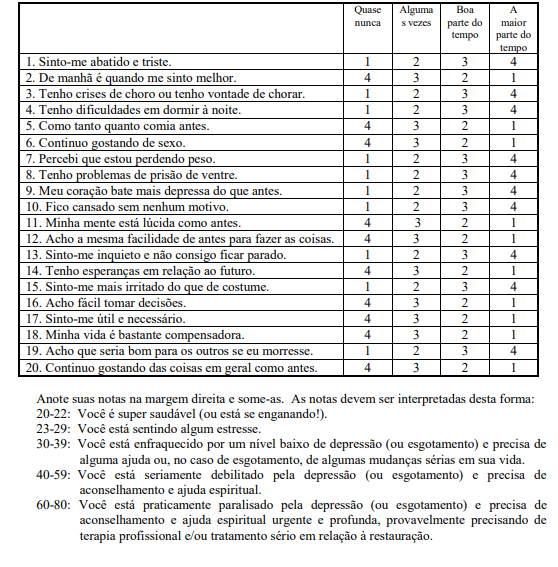


**ANNEX 6 -** Post-Covid Functional Status Scale.

| **POST-COVID-19 FUNCTIONAL STATUS SCALE** | |
| --- | --- |
| **1.Sobrevivência** |  |
| 1.1 Did the patient die after the diagnosis of COVID-19? | □ Yes □ No |
| **2. Constant Care** |  |
| 2.1 Do you need constant care? | □ Yes □ No |
| **3. Basic Activities of Daily Living (ADL)** |  |
| 3.1 Is it essential to have assistance to eat? | □ Yes □ No |
| 3.2 Is it essential to have assistance in using the bathroom? | □ Yes □ No |
| 3.3 Is it essential to have assistance for daily hygiene routine? | □ Yes □ No |
| 3.4 Is it essential to have walking assistance? | □ Yes □ No |
| **4. Instrumental Activities of Daily Living (IAVC)** |  |
| 4.1 Is assistance in performing basic household tasks important for daily life essential? | □ Yes □ No |
| 4.2 Is assistance essential to make local trips? | □ Yes □ No |
| 4.3 Is assistance essential to make local purchases? | □ Yes □ No |
| **5. Participation in the social roles of the** |  |
| 5.1 Is it essential to adapt to perform tasks/activities at home or at work/study because you are unable to perform them alone? (e.g. resulting in a change in the level of responsibility, change in work/study from full-time to part-time) | □ Yes □ No |
| 5.2 Do you occasionally need to avoid or reduce tasks/activities at home or at work/study or do you need to distribute them over time? (even though you are able to perform all these activities)? | □ Yes □ No |
| 5.3 Can't you take good care of loved ones like before? (children, partners, parents, grandchildren or other dependents) | □ Yes □ No |
| 5.4 Since the diagnosis of COVID-19, have there been problems in relationships or have you been isolated? (communication problems, difficulties in the relationship with people at home or at work/study, loss of friendships in isolation) | □ Yes □ No |
| 5.5 Are you restricted to participating in social and leisure activities? (including hobbies and interests such as going to a restaurant, bar, cinema, sightseeing, playing games, reading books) | □ Yes □ No |
| **6. SYMPTOMCHECKLIST** |  |
| 6.1 Do you experience symptoms during daily tasks/activities that need to be avoided, reduced, or distributed over time? | □ Yes □ No |
| 6.2 Do you have any symptoms resulting from COVID-19 that do not cause functional limitations? | □ Yes □ No |
| 6.3 Do you have difficulty relaxing or do you perceive COVID-19 as a trauma? | □ Yes □ No |

**APPENDAGES**

**APPENDIX**  A - Free and Informed Consent Form (TCLE)

**Free and Informed Consent Form - TCLE**

*Clarification*

This is an invitation for you to participate in the research: "Efficacy of home inspiratory muscle training in post-covid-19 patients: randomized clinical trial", which has as responsible researcher Prof. Patrícia Angélica de Miranda Silva Nogueira.

This research aims to evaluate the effectiveness of a home inspiratory muscle training protocol in improving respiratory muscle strength, dyspnea and quality of life of post-Covid-19 patients.

If you decide to participate, you should answer questions about personal information without identifying it, medication use, and life habits. You will measure weight, height; Check your blood pressure, heart beats and the amount of oxygen in your body and also measure the circumference of the potato on your leg. A test will also be performed for you to squeeze into a spring and blow into a device to check the strength of your muscles and the function of your lung. You will also perform a test to walk for six minutes and answer questionnaires about your level of physical activity, your quality of life, anxiety and depression, and post-Covid-19 functional status. You will also answer a test to check your memory capacity and research into shortness of breath and tiredness in your legs. These tests are not difficult and will not harm health. All measures will be carried out in 1 day that will be previously scheduled, so that you are not harmed. All exercises will be explained in advance and any instruction regarding clothing or footwear will be given in advance.

To perform the home exercises, you will receive a POWERbreathe device®, and you must perform the exercises, twice a day (morning and afternoon),sete days a week, for 6 consecutive weeks. In each session you must perform 30 repetitions of the exercise and you can rest every 3 repetitions for 30 seconds to 1 minute. In the first week the exercises will be performed in two ways for 6 weeks: with 30% of the maximum strength of the inspiratory muscles or without load, with increased load every week. The way to perform the exercise will be chosen by raffle for each volunteer. Volunteers will be reevaluated after 6 and 24 weeks of training.

The activities performed will be non-invasive, that is, procedures involving cutting, penetration of instruments, blood collection or that may generate pain or discomfort will NOT be performed. However, you may tire during evaluations and training, which will be minimized with rest intervals between procedures. This research presents minimal risks to participants. They may feel constrained during the interview, considering the nature of the content of some questionnaires, which they may refuse to answer, or because of the difficulty in performing any specific evaluation. They may present some discomforts such as headache, vertigo, nausea, blood pressure oscillation and even a small imbalance due to the requirement of certain physical tests and training, however the evaluators will use contraindication criteria or interruptions of the tests when necessary, to minimize the risks to the health of the participants.

The benefits of participating in the research are: knowledge of the result of a therapeutic evaluation for

**_________**

their state of health, a greater understanding of the physiological effects of training for respiratory muscles, in order to make a more adequate prescription of this training, as well as the verification of the usefulness of this exercise program for patients in the post-covid-19 period.

All information obtained will be confidential and your name will not be identified at any time. The data will be stored in a safe place and the dissemination of the results will be made in such a way as not to identify the volunteers.

The research will be funded by the researchers and that the devices will be provided free of charge to the study participants.

In case of any problem that you may have related to the research, you will be entitled to free assistance that will be provided by the responsible researcher.

Throughout the research period you can answer your questions by calling the doctoral doctor Gabriely Azevêdo Gonçalo Silva, (84)99939-0004.

This data will be stored by the researcher responsible for this research in a safe place and for a period of 5 years.

If you have any expenses for your participation in this research, they will be assumed by the researcher and reimbursed to you.

If you suffer any damage arising from this research, whether it is immediate or late, anticipated or not, you will be indemnified.

Any questions about the ethics of this research you should call the Research Ethics Committee – an institution that evaluates the ethics of research before they begin and provides protection to its participants – from the Federal University of Rio Grande do Norte, at (84) 3215-3135 / (84) 9.9193.6266, through [the e-mail cepufrn@reitoria.ufrn.br or through the contact](mailto:cepufrn@reitoria.ufrn.br) form of the < website [www.cep.propesq.ufrn.br](file:///D:\Documentos\UFRN\Doutorado\Projeto%20-%20ATAQ\www.cep.propesq.ufrn.br) > . You can also go in person to the cep headquarters, Monday to Friday, from 08:00 to 12:00 and from 14:00 to 18:00, at the Federal University of Rio Grande do Norte, Central Campus. New Lagoon. Natal/RN. ZIP Code 59078-970.

This document was printed in two ways. One will stay with you and the other with the responsible researcher Prof. Patrícia Angélica de Miranda Silva Nogueira.

*Free and Informed Consent*

After being informed about the objectives, importance and how the data will be collected in this research, in addition to knowing the risks, discomforts and benefits that it will bring to me and having become aware of all my rights, I agree to participate in the research "Efficacy of home inspiratory muscle training in patients post-covid-19: randomized clinical trial" , and authorize the disclosure of information provided by me in congresses and/or scientific publications as long as no data can identify me.

Datiloscopic impression of the participant

Christmas, ____/____

**Subscription of the survey participant**

___________________________________________

*Statement by the researcher* in charge "Efficacy of home inspiratory muscle training in post-covid-19 patients: randomized clinical trial" declare that I take full responsibility for faithfully fulfilling the

**_________**

methodological procedures and rights that were clarified and guaranteed to the participant of this study, as well as maintain secrecy and confidentiality about his identity.

I also declare to be aware that in failure to comply with the commitment made here in violation of the norms and guidelines proposed by Resolution 466/12 of the National Health Council - CNS, which regulates research involving human beings.

Christmas, ___/____

**Signature of the responsible researcher**

___________________________________________________________________________

**APPENDIX B** - Training control

**TMI CONTROL**

**Name:____**

**Initial rating: ___/_____**

| **1st week** Day 1  ___/___/__ | Day 2  ___/___/__ | Day 3  ___/___/__ | Day 4  ___/___/__ | Day 5  ___/___/__ | Day 6  ___/___/__ | **Rest** |
| --- | --- | --- | --- | --- | --- | --- |
| 1st session | | | | | | |
| 2nd session | | | | | | |

| **2nd week** Day 1  ___/___/__ | Day 2  ___/___/__ | Day 3  ___/___/__ | Day 4  ___/___/__ | Day 5  ___/___/__ | Day 6  ___/___/__ | **Rest** |
| --- | --- | --- | --- | --- | --- | --- |
| 1st session | | | | | | |
| 2nd session | | | | | | |

| **3rd week** Day 1  ___/___/__ | Day 2  ___/___/__ | Day 3  ___/___/__ | Day 4  ___/___/__ | Day 5  ___/___/__ | Day 6  ___/___/__ | **Rest** |
| --- | --- | --- | --- | --- | --- | --- |
| 1st session | | | | | | |
| 2nd session | | | | | | |

| **4th week** Day 1  ___/___/__ | Day 2  ___/___/__ | Day 3  ___/___/__ | Day 4  ___/___/__ | Day 5  ___/___/__ | Day 6  ___/___/__ | **Rest** |
| --- | --- | --- | --- | --- | --- | --- |
| 1st session | | | | | | |
| 2nd session | | | | | | |

| **5th week** Day 1  ___/___/__ | Day 2  ___/___/__ | Day 3  ___/___/__ | Day 4  ___/___/__ | Day 5  ___/___/__ | Day 6  ___/___/__ | **Rest** |
| --- | --- | --- | --- | --- | --- | --- |
| 1st session | | | | | | |
| 2nd session | | | | | | |

| **6th week** Day 1  ___/___/__ | Day 2  ___/___/__ | Day 3  ___/___/__ | Day 4  ___/___/__ | Day 5  ___/___/__ | Day 6  ___/___/__ | **Rest** |
| --- | --- | --- | --- | --- | --- | --- |
| 1st session | | | | | | |
| 2nd session | | | | | | |

**APPENDIX C** - Clinical evaluation sheet

**CLINICAL EVALUATION FORM**

**POST-COVID-19 PROJECT**

| **PERSONAL DATA** | | | | |
| --- | --- | --- | --- | --- |
| Name: | | RG: | Genus: □ M □ F | |
| Naturalness: | | Date of birth: | CPF: | |
| Address: | |  |  | |
| Contact phones: | | Email: |  | |
| Profession: | | □ Retiree |  | |
| Education (years): | |  |  | |
| Race / skin color: □ white □ black □ amarela | | □ brown | □ indian □ not declare | |
| Family income: □ less than 1 minimum wage □ between 1 and 3 minimum wages □between 4 and 9 | | minimum wages | □ 10 or more min salaries | |
| Lives with: □Family □Alone □ Other | |  |  | |
| **PERSONAL HISTORY** | | | | |
| □ SAH □ DM □ Obesity □ □ amientarism □ CI | | □ OFF | □ COPD □Asthma | |
| □ IRC □ Others: | |  |  | |
| **LIFE HABITS** | | | | |
| □ Alcohol Consumption: □ yes □ not □ less than once a month □ Once or more per month  □ Tobacco consumption: □ yes □ no | | | | |
| □ Tobacco consumption: □ yes □ not □ daily □ less than daily  IF you smoke, how long? How many packs a day?  And in the past, have you smoked? □ yes, □ yes, less than daily □ no, Never smoked | | | | |
| **COVID-19** | | | | |
| □ Date of diagnosis: □ Date of hospitalization:  □Symptoms in hospital admission: | | |  | |
| □ Total number of days of hospitalization: □ Number of ICU days:  Date of hospital discharge: | | | | |
| The sr was □ yes □ no □ If yes, what was the number of days intubated?  Oxygen therapy: □ yes □ no □ Time:  Made/uses corticosteroid: □ yes □ not □Time:  It currently uses oxygen: □ yes □ no  □ Current symptoms: | | | | |
| **CLINICAL EXAMINATION** | | | | |
|  | Weight: _____ | | |  |
| Self-reported health (SAR) | | In general, how do you assess your health? | □ very good □ good □ regular □ bad □ bad | |
| Cough* | | Are you coughing? | □ 'm not coughing  □ yes, dry cough, irritating  □ yes, cough with phlegm (What is the crolla? __________________________________) | |
| Thoracic expansion** | | Can you breathe deeply? | □ 't  □ yes, but with difficulty  □ yes, without any difficulty | |
| Dispneia (Modified Borg) | | How do you assess your feeling of shortness of breath now? On a scale from zero to ten, where (read the alternatives) | □ 0 no feeling of shortness of breath  □ 0.5 very, very light  □ 1 very light  □ 2 light  □ 3 moderate  □ 4 little intense  □ 5 intense  □ 6  □ 7 very intense  □ 8  □ 9 very, very intense  □ 10 not maximum | |
| Dyspnea (MRC - Medical Research Council) | | How does your condition assess your feeling of shortness of breath? (choose an alternative) | □ 1) only suffers from shortness of breath during intense exercises.  □ 2) suffers from shortness of breath when walking hastily or climbing a light ramp.  □ 3) walks slower than people of the same age because of shortness of breath or have to stop to breathe even when walking slowly.  □ 4) to breathe after walking less than 100 meters or after a few minutes.  □ 5) feels so short of breath that he no longer leaves the house, or when he is getting dressed. | |

| **PALMAR GRIP STRENGTH** | | | | | | |
| --- | --- | --- | --- | --- | --- | --- |
|  | Data | First | Second | Third | 4th | 5th |
| Rating 1 |  |  |  |  |  |  |
| Rating 2 |  |  |  |  |  |  |
| Rating 3 |  |  |  |  |  |  |
| Rating 4 |  |  |  |  |  |  |

| **LUNG FUNCTION** | | | | | | |
| --- | --- | --- | --- | --- | --- | --- |
| **Evaluation** | **Spirometry** | 1st test | 2nd test | 3rd test | % expected | Expected value |
| Rating 1  Data: | CVF |  |  |  |  |  |
|  | ALI |  |  |  |  |  |
|  | VEF1 |  |  |  |  |  |
|  | PFE |  |  |  |  |  |
|  | VEF1/CVF% |  |  |  |  |  |
| Rating 2  Data: | CVF |  |  |  |  |  |
|  | ALI |  |  |  |  |  |
|  | VEF1 |  |  |  |  |  |
|  | PFE |  |  |  |  |  |
|  | VEF1/CVF% |  |  |  |  |  |
| Rating 3  Data: | CVF |  |  |  |  |  |
|  | ALI |  |  |  |  |  |
|  | VEF1 |  |  |  |  |  |
|  | PFE |  |  |  |  |  |
|  | VEF1/CVF% |  |  |  |  |  |
| Rating 4  Data: | CVF |  |  |  |  |  |
|  | ALI |  |  |  |  |  |
|  | VEF1 |  |  |  |  |  |
|  | PFE |  |  |  |  |  |
|  | VEF1/CVF% |  |  |  |  |  |

| **RESPIRATORY MUSCLE STRENGTH** | | | | | | |
| --- | --- | --- | --- | --- | --- | --- |
|  |  | 1st value | 2nd value | 3rd value | % expected | Expected value |
| Rating 1  Data: | The MON. |  |  |  |  |  |
|  | The Mepmax. |  |  |  |  |  |
| Rating 2  Data: | The MON. |  |  |  |  |  |
|  | The Mepmax. |  |  |  |  |  |
| Rating 3  Data: | The MON. |  |  |  |  |  |
|  | The Mepmax. |  |  |  |  |  |
| Rating 4  Data: | The MON. |  |  |  |  |  |
|  | The Mepmax. |  |  |  |  |  |

**6-Minute Walk Test (6MWT)**

| **Evaluation** |  | Rest | 6 minutes |
| --- | --- | --- | --- |
| Rating 1  Data: | And O |  |  |
|  | FC |  |  |
|  | SPO2 |  |  |
|  | SEX |  |  |
|  | BORG-D |  |  |
|  | BORG-F |  |  |
| Rating 2  Data: | And O |  |  |
|  | FC |  |  |
|  | SPO2 |  |  |
|  | SEX |  |  |
|  | BORG-D |  |  |
|  | BORG-F |  |  |
| Rating 3  Data: | And O |  |  |
|  | FC |  |  |
|  | SPO2 |  |  |
|  | SEX |  |  |
|  | BORG-D |  |  |
|  | BORG-F |  |  |
| Rating 4  Data: | And O |  |  |
|  | FC |  |  |
|  | SPO2 |  |  |
|  | SEX |  |  |
|  | BORG-D |  |  |
|  | BORG-F |  |  |

| **INSPIRATORY MUSCLE TRAINING** | | | | | | |
| --- | --- | --- | --- | --- | --- | --- |
|  | **Week 1 (30% PImax)** | **Week 2 (40% PImax.)** | **Week 3 (50% Pimax.)** | **Week 4 (60% Pimax.)** | **Week 5 (60% Pimax)** | **Week 6 (60% Pimax.)** |
| **Training load** |  |  |  |  |  |  |
